# Supplementary material for: Attrition and associated factors among children living with HIV at a tertiary hospital in Eritrea: a retrospective cohort analysis
Source: BMJ Paediatr Open. 2022 Jul 1;6(1):e001414. doi: 10.1136/bmjpo-2022-001414 (PMC9252199; doi:10.1136/bmjpo-2022-001414)
Supplement: Author's manuscript [file bmjpo-2022-001414.draft_revisions.pdf]

BMJ Paediatrics Open

BMJ Paediatrics Open is committed to open peer review. As part of this commitment we make the peer review history of every article we publish publicly available.

When an article is published we post the peer reviewers' comments and the authors' responses online. We also post the versions of the paper that were used during peer review. These are the versions that the peer review comments apply to.

The versions of the paper that follow are the versions that were submitted during the peer review process. They are not the versions of record or the final published versions. They should not be cited or distributed as the published version of this manuscript.

BMJ Paediatrics Open is an open access journal and the full, final, typeset and author-corrected version of record of the manuscript is available on our site with no access controls, subscription charges or pay-per-view fees (http://bmjpaedsopen.bmj.com).

If you have any questions on BMJ Paediatrics Open's open peer review process please email info.bmjpo@bmj.com

BMJ Paediatrics Open

Attrition and associated factors among HIV-infected children at Orotta National Referral and Teaching Hospital, Asmara, Eritrea: A 15-year cohort analysis

Journal:	*BMJ Paediatrics Open*
Manuscript ID	bmjpo-2022-001414
Article Type:	Original research
Date Submitted by the Author:	23-Jan-2022

Complete List of Authors:	Mengistu, Samuel; Nakfa Hospital, General Medicine; Orota School of Medicine and Dentistry, Medicine<br>Ghebremeskel, Ghirmay; Nakfa Hospital, General Medicine<br>Rezene, Aron; Edaga Hamus Hospital, Ministry of health Maekel branch, Asmara, Eritrea<br>Idris, Mahmud ; Department of pediatrics and child health, Orotta College of Medicine and Health Sciences, Asmara, Eritrea<br>Tikue, Tsegereda; Department of pediatrics and child health, Orotta College of Medicine and Health Sciences, Asmara, Eritrea<br>Hamida, Mohammed ; Unit of Medical Microbiology, Orotta College of Medicine and Health Sciences (OCMHS).<br>Achila, Oliver ; Unit of Clinical Laboratory Science, Orotta College of Medicine and Health Sciences (OCMHS).

Keywords:	Virology, HIV, Mortality, Epidemiology

SCHOLARONE™
Manuscripts

I, the Submitting Author has the right to grant and does grant on behalf of all authors of the Work (as defined in the below author licence), an exclusive licence and/or a non-exclusive licence for contributions from authors who are: i) UK Crown employees; ii) where BMJ has agreed a CC-BY licence shall apply, and/or iii) in accordance with the terms applicable for US Federal Government officers or employees acting as part of their official duties; on a worldwide, perpetual, irrevocable, royalty-free basis to BMJ Publishing Group Ltd ("BMJ") its licensees and where the relevant Journal is co-owned by BMJ to the co-owners of the Journal, to publish the Work in this journal and any other BMJ products and to exploit all rights, as set out in our licence.

The Submitting Author accepts and understands that any supply made under these terms is made by BMJ to the Submitting Author unless you are acting as an employee on behalf of your employer or a postgraduate student of an affiliated institution which is paying any applicable article publishing charge ("APC") for Open Access articles. Where the Submitting Author wishes to make the Work available on an Open Access basis (and intends to pay the relevant APC), the terms of reuse of such Open Access shall be governed by a Creative Commons licence – details of these licences and which Creative Commons licence will apply to this Work are set out in our licence referred to above.

Other than as permitted in any relevant BMJ Author's Self Archiving Policies, I confirm this Work has not been accepted for publication elsewhere, is not being considered for publication elsewhere and does not duplicate material already published. I confirm all authors consent to publication of this Work and authorise the granting of this licence.

Confidential: For Review Only

Attrition and associated factors among HIV-infected children at Orotta National Referral and Teaching Hospital, Asmara, Eritrea: A 15-year cohort analysis

Samuel Tekle Mengistu[1*], Ghirmay Ghebrekidan Ghebremeskel[2], Aron Rezene[3], Mahmud Mohammed Idris[4], Tsegereda Gebrehiwot Tikue[5], Mohammed Elfatih Hamida[6], Oliver Okoth Achila[7]

[1]**Samuel Tekle Mengistu**. Nakfa Hospital, Ministry of health Northern Red Sea branch, Nakfa, Eritrea. Email: teklesam7@gmail.com

[2]**Ghirmay Ghebrekidan**. Nakfa Hospital, Ministry of health Northern Red Sea branch, Nakfa, Eritrea. Email: GhirmayGGG@gmail.com

[3]**Aron Rezene**. Edaga Hamus Hospital, Ministry of health Maekel branch, Asmara, Eritrea

Email: aronrezene19@gmail.com

[4]**Mahmud Mohammed Idris.** Department of pediatrics and child health, Orotta College of Medicine and Health Sciences, Asmara, Eritrea. Email: mahmudfil@yahoo.com

[5]**Tsegereda Gebrehiwot Tikue.** Department of pediatrics and child health, Orotta College of Medicine and Health Sciences, Asmara, Eritrea. Email: proftsigeghebrehiwet@gmail.com

[6]**Mohammed Elfatih Hamida.** Unit of Medical Microbiology, Orotta College of Medicine and Health Sciences (OCMHS). Email: mohelfatih77@gmail.com

[7]**Oliver Okoth Achila.** Unit of Clinical Laboratory Science, Orotta College of Medicine and Health Sciences (OCMHS). Email: oliverachila@gmail.com

*Corresponding author: **Samuel Tekle Mengistu**, email: teklesam7@gmail.com, Nakfa Hospital, Northern Red Sea Ministry of Health, Asmara, Eritrea.

Word count: 5,285

Confidential: For Review Only

What's Known about the subject?

Studies in low- and medium-income countries (LMICs) of SSA and Asia have demonstrated that retention of HIV infected children in care is a major challenge. Multiple evaluations have suggested that in the context of the rapid scale-up of cART, reducing attrition (Lost to follow-up (LTFU) and mortality) rate is a key programmatic priority.

What the study adds?

- Steep decline in enrolment potentially highlight the fact that effective PMTCT services in prenatal clinics reduced MTCT thereby reducing incident pediatric HIV infection.
- A quarter of the patients weren't retained in care.
- Attrition in the first 2 years after enrollment was largely due to death.
- Higher incidence of attrition was recorded among those enrolled in recent years; malnutrition; advanced HIV disease (III or IV) at enrollment; initiation of backbones other than AZT+3TC, and residence outside Zoba Maekel.

Confidential: For Review Only

Abstract

Background: Reducing attrition in pediatric HIV positive patients in combined antiretroviral therapy (cART) programs in sub–Saharan Africa (SSA) is a challenge in the region. This study explored the rates and predictors of attrition in children initiated on cART at the principal follow-up facility in Asmara, Eritrea.

Methods: This was a retrospective cohort study using routinely collected programmatic data among all pediatric patients on cART between 2005 and 2020 conducted at Orotta National Referral and Teaching Hospital. Kaplan–Meier estimates of the likelihood of attrition and multivariate Cox proportional hazard models were used to assess factors associated with attrition. All *p*-values were 2-sided and the level of significance was set at $p < 0.05$ for all analyses.

Results: Among the 710 participants were included in our analysis. After 5364 person-years follow-up, attrition occurred in 172 (24.2%) patients. The crude incidence rate of attrition was 3.2 events /100 person-years (PY), mortality rate and lost to follow-up (LTFU) were 2.7/100 PY and 1.2/100 PY, respectively. In the adjusted Cox proportional hazards model, independent predictors of attrition were male gender (AHR=1.6, 95% CI 1–2.4), residence outside Zoba Maekel (AHR=1.5, 95% CI, 1–2.3); Later enrollment years (2010-2015: AHR=3.2, 95% CI, 1.9-5.3) (>2015: AHR=6.1, 95% CI, 3-12.2); WHO BAZ < -2 (AHR = 1.4, 95% CI, 0.9-2.1); advanced HIV disease (WHO III or IV) at enrollment (AHR = 2.2, 95% CI, 1.2-3.9), and Initiation on AZT+3TC or other cART backbones (UHR = 2, 95% CI, 1.2-3.2). In contrast, a reduced likelihood of attrition was observed in children with a record of cART changes (UHR=0.2, 95% CI, 0.15-0.4).

Conclusion: Low incidence of attrition was recorded in this program. However, the high mortality rate in the first 24 months of treatment and late presentation are worrisome. Therefore, the need for data-driven interventions designed to improve program quality and outcomes are of high priority.

Key words: Risk Factors, Pediatrics, HIV/AIDS, Attrition, lost to follow-up, Eritrea

Confidential: For Review Only

Introduction

Despite progress in HIV prevention, care, and treatment, HIV in children remains a major public health burden. A recent Joint United Nations Programme on HIV/AIDS (UNAIDS) report suggested that of the 37.7 million [30.2–45.1 million], people living with HIV (PLWH) in 2020; Children < 14 years' account for ~1.8 million [1.3–2.2 million] [1]. In the same period, 180,000 children were newly infected and 110,000 children died of HIV/AIDS. Importantly, a disproportionate number of these children were from sub-Saharan Africa (SSA) where resources are limited and where infectious disease burden is high.

In many ways, the prevailing situation undermines the UNAIDS 90-90-90 target to end HIV/AIDS epidemic by 2030, [2]. Multiple studies in low- and medium-income countries (LMICs) in SSA and Asia have demonstrated that a large proportion of HIV-infected children continue to present to care centers with advanced HIV disease, although cART coverage is rapidly expanding, and retention is a major challenge [3 4]. Altogether, cART enables PLWH to live long and healthy lives and marked improvements have been registered in almost all principal landmarks in the HIV care cascade globally, empirical data suggests that children living with HIV (CLHIV), particularly young adolescents, are still experiencing an upswing in HIV/AIDS-related mortality [5].

Although investigators questioned, and with good reason, the practicality of the UNAIDS 90-90-90 target timeline [2]; consensus on the underlying "diagnose (early) - treat (promptly) –retain (to ensure viral suppression) paradigm" is broad and unwavering. While it's true that diagnosis and cART coverage in pediatrics has increased [1]; multiple evaluations have suggested that in the context of the rapid scale-up of cART, reducing attrition (Lost to follow-up (LTFU) and mortality) rate is a key programmatic priority. This concern emanates from data showing that attrition is high among younger (children < 14 years) – approximately 14% at 12 months follow-up and 22% by 36 months [6 7]. To emphasize this concern, they have argued that retention is not only necessary to ensure ongoing

Confidential: For Review Only

receipt of cART; but it also allows clinicians to evaluate the emergence of medication toxicity; the opportunity to prevent, manage or treat opportunistic infections (IOs), detect treatment failure; and initiate prompt switching of cART. Additional benefits include ancillary services such as social support and counseling. In this regard, attrition is not the only a nuisance encountered in the analysis of longitudinal or retrospective data; but it's an important study outcome itself. Therefore, understanding attrition rates and associated factors among HIV-infected children in jurisdictions across SSA will remain a principal research concern in the foreseeable future. Data gathered from such studies can be used as a benchmark for gauging programmatic successes and weaknesses' [6] and to identify critical gaps and opportunities for context-specific interventions aimed at minimizing attrition and maximizing retention.

Therefore, the paucity of empirical data on the determinants of attrition and program retention of children starting cART not only impedes research efforts; it also undermines context-based planning of interventions designed to reduce attrition in treatment centers. This, we have to concede, is the prevailing situation in Eritrea – a country with one of the lowest HIV prevalence in SSA [8]. Therefore, the primary aim of this study was to describe the incidence of attrition and associated factors among HIV-infected children in Asmara, Eritrea. Secondarily, we explored multiple temporal trends associated with program performance, mortality, and enrolment rates.

Methodology

Study design and setting

A retrospective cohort study was conducted in the pediatric HIV/AIDs follow-up clinic in National Pediatric Referral Hospital (NPRH). HIV/AIDs follow-up clinic in NPRH was commissioned in 2005, making it the first institution in Eritrea to offer cART to CLHIV. Before the decentralization of services to other zones (2010), NPRH (in the Maekel zone) was the only institution offering cART

Confidential: For Review Only

to CLHIV in the country. In total, 822 children under the age of 15 years received service and /or have been enrolled at the clinic since its inception.

Data collection and approach

We used the data collected in the ONRTH pediatric cART and follow-up program electronic database (ART DHIS 2). The retrieved data was robustly reviewed and then cross-referenced with patients' clinical chart registries. Data retrieved from this repository was exported to Microsoft (MS) excel platform. Baseline data collected included: date of birth/age at enrolment, gender, address, and cART initiation date, initial cART regimen, presence of tuberculosis (TB), baseline WHO clinical stage, baseline CD4 count, weight, and hemoglobin (Hg) concentration, and CBC. Height is not available in the electronic medical records, thus being retrieved from clinical chart registries systematically. WHO standard deviations for weight for age,, height for age and Body mass index (BMI) for age parameters were computed using the WHO Standard deviation for emergency nutritional assessment software (www.nutrisurvey.net/ena/ena.html). Recruitment details are illustrated in Figure 1.

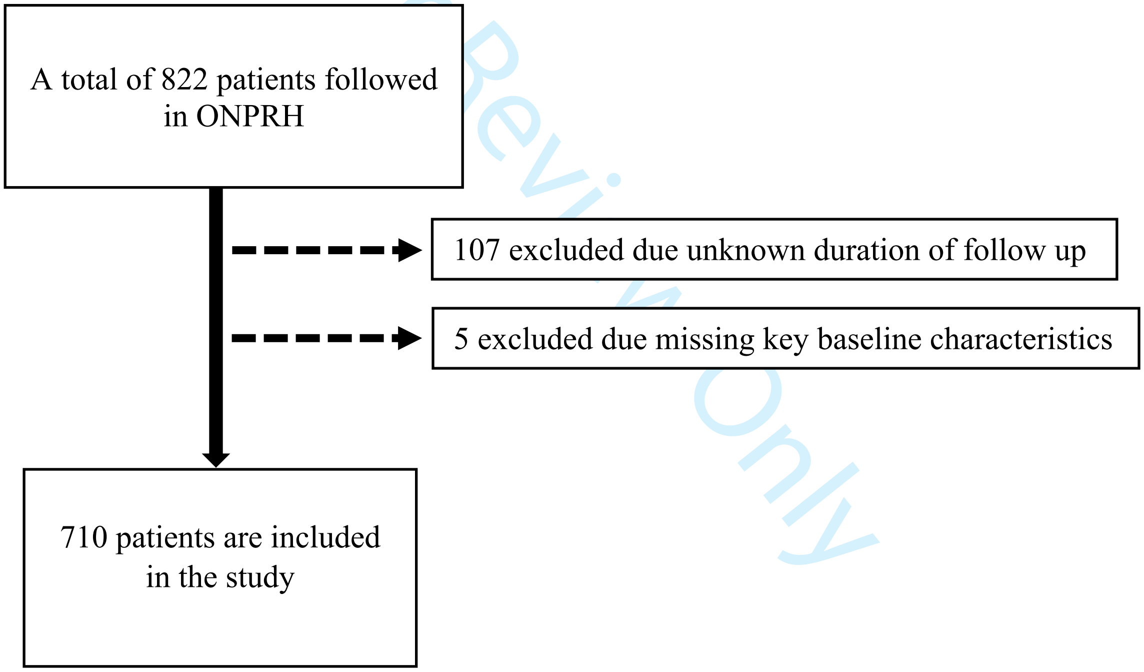

Figure 1: Flow chart for study participant recruitment.

Confidential: For Review Only

Operational Definitions

- Death event was defined as deaths occurring after enrollment in follow-up, but before December 31st, 2020 [9-11].
- Patients were considered LTFU if they had missed a scheduled visits for ≥ 3 consecutive months [9].
- Attrition was defined as children who were LTFU or died during follow-up in clinic [6 12].
- Retention – children who were alive and on follow-up in the clinic at the end of the study period and children who were transferred out at any time during study period [12].
- Advanced HIV disease was defined as CD4 cell count <200 cells/mm^3 and/or WHO stage 3 or 4 events for patients > 5 years of age [13].
- Multiple measures were used to measure nutritional status. These included weight-for-age Z-score (WAZ), height-for-age (HAZ), and BMI-for -age Z-score (BAZ). Under nutrition underscores BAZ < − 2 SD; WAZ ≤−2 z-score, and/or HAZ ≤−2 z-score.

Data analysis

The retrieved data was exported to IBM SPSS (version 26) and STATA version 12.0 (STATA Corporation, College Station, TX), where it was organized and cleaned for analysis. Descriptive statistics for categorical variables were analyzed using chi-square/Fisher's exact test and summarized using frequency and percentage. Depending on data distribution, quantitative data was summarized using mean (±SD) or median (IQR). Suitable parametric or nonparametric statistics were subsequently employed to evaluate differences. Furthermore, Kaplan-Meier curves were used to estimate retention rates at different intervals of follow-up by gender, address, cohort year, adjusted hazard ratio (AHD), initial cART backbone, and record of cART change. All LTFU and transfer-outs were censored on the date of their last visit. Univariate and multivariate Cox regression model was

Confidential: For Review Only

implemented for assessing the variables that best predict attrition. Finally, a multivariate Cox regression model was used to factor in time-to-event and control confounders. The final results are presented as AHR with 95% confidence interval (CI) and P value less than 0.05 is considered significant.

Ethical consideration

Ethical approval was obtained from the Ministry of Health (MOH) research Ethics and Protocol review committee with a letter of reference: 02/05/21. All data collected from the records of respondents will be kept confidential at all times. Consent to participate was not obtained from the patients or guardians because the study was based on anonymized patient records and this was waived by the ethical committee. All procedures of the study also followed the recommendation of the Declaration of Helsinki Convention.

Patient and public involvement

Patients or the public WERE NOT involved in the design, or conduct, or reporting, or dissemination plans of our research.

Results

From September 2005- December 2020, a total of 822 children living with HIV (CLHIV) were enrolled in ONPRH pediatric cART treatment and follow-up program. After eligibility evaluation, 710 (86.3%) participants were included in our analysis. Stratification of enrollment with respect to specific time frames indicated that 480(67.6%), 180(25.4%), and 50(7.0%) were enrolled into care between 2005-2010; 2011-2015; and 2016-2020, respectively. (Table 1). Eligible participants were followed for a total of 5,364 person-years follow-up (PYFU). In this period, 65 (9.2%) died; 107 (15.1%) were LTFU; 153 (21.5%) were transferred out and 385 (54.2%) are currently on treatment.

Confidential: For Review Only

Table 1. Baseline characteristics of HIV-infected children and adolescents in the ONPRH cART treatment center, Asmara, Eritrea (2005–2020).

Patients' characteristics	Total N (%)	2005-2010	2011-2015	2016-2020	P-value
**Number enrolled**	710	480(67.6)	180(25.4)	50(7.0)	
**Gender**					
Male	374 (52.7)	237 (63.4)	111 (29.7)	26 (7)	0.01 (7.9)
Female	336 (47.3)	243 (72.3)	69 (20.5)	24 (7.1)	
**Age on enrollment in years, median (IQR)**	6 (3-9)	5 (3-8)	7 (4-10)	5 (2-11)	
<=5 years	274 (38.6)	198 (72.3)	52 (19)	24 (8.8)	<0.001
6-10 years	284 (40)	202 (71.1)	72 (25.4)	10 (3.5)	(27.5)
>10 years	152 (21.4)	80 (52.6)	56 (36.8)	16 (10.5)	
**Address**					
Maekel	516 (72.7)	358 (69.4)	124 (24)	34 (6.6)	0.25 (2.7)
Outside Maekel	194 (27.3)	122 (62.9)	56 (28.9)	16 (8.2)	
**Disease stage**					
Early	191(26.9)	134 (70.2)	46 (24.1)	11 (5.8)	0.5 (1)
Advanced	519 (73.1)	346 (66.7)	134 (25.8)	39 (7.5)	
**TB- status**					
Symptomatic and under treatment	16 (2.2)	7 (43.8)	6 (37.5)	3 (18.8)	0.5 (5.9)
Not symptomatic	625 (88)	443 (70.9)	135 (21.6)	47 (7.5)	
**CD4 count, Median (IQR)**	274 (150-442)	265 (151-392.7)	307 (137-555)	340 (200-888)	0.012
**CD4 percentage, Median (IQR)**	12 (7.4-17)	11.1 (7.1-16.5	13.6 (9-21)	14.3 (7.5-21.9)	0.007
**Hemoglobin, mean(±SD)**	11.8(±13.5)	12.5 (±17.2)	10.6 (±2.1)	11 (±1.6)	0.060
Anemia	264 (37.2)	155 (58.7)	89 (33.1)	20 (7.6)	0.3 (2.2)
No Anemia	313 (44.1)	197 (62.9)	88 (28.1)	28 (8.9)	
**WAZ, median (IQR)**	-2.6 (-3.6--1.7)	-2.5 (-3.5--1.7)	-2.8 (-3.8--1.7)	-2.9 (-3.8-1.6)	0.263
WAZ=>-2	302 (42.5)	98 (69.5)	32 (22.7)	11 (7.8)	0.7 (0.5)
WAZ <-2	141 (19.9)	202 (66.9)	78 (25.8)	22 (7.3)	
**HAZ, median (IQR)**	-2.75 (-3.8--1.7)	-2.6 (-3.7---1.5)	-2.9 (-4--1.8)	-3(-3.9--1.8)	0.063
HAZ=>-2	461 (64.9)	161 (72.2)	49 (22)	13 (5.8)	0.1 (4.3)
HAZ <-2	223 (31.4)	296 (64.2)	129 (28)	36 (7.8)	
**BAZ, median (IQR)**	-1.58 (-2.8--0.69)	-1.5 (-2.9--0.5)	-1.6 (-2.7--1)	-1.7 (-3--0.6)	0.385
BAZ=>-2	378 (53)	257 (68)	97 (25.7)	24 (6.3)	
BAZ <-2	247 (34.9)	162 (65.6)	64 (25.9)	21 (8.5)	0.5 (1)
**cART backbone**					
AZT+3TC	508 (71.7)	347 (68.3)	148 (29.1)	13 (2.6)	
Other backbones[a]	201 (28.3)	133 (66.2)	31 (15.4)	37 (18.4)	<0.001 (62)
**NNRTI**					
EFV	366 (51.5)	262 (71.6)	90 (24.6)	14 (3.8)	<0.001 (13)
NVP	340 (48.4)	216 (63.5)	14 (3.8)	36 (10.6)	
**Current cART regimen**					
First line cART	633 (89.2)	429 (67.8)	157 (24.8)	47 (7.4)	0.3 (1.9)
Second line cART	77 (10.8)	51 (66.2)	23 (29.9)	3 (3.9)	
**Adherence**					
Suboptimal Adherence	91 (12.3)	46 (50.5)	39 (42.9)	6 (6.6)	<0.001 (17)
No record	619 (87.2)	434 (70)	141 (22.8)	44 (7.1)	
**cART change**	516 (72.1)	**340 (65.9)**	**148 (28.7)**	**28 (5.4)**	**<0.001 (1.2)**
No cART change	172 (24.2)	120 (69.8)	30 (17.4)	22 (12.8)	
Duration of follow up in years, Median (IQR)	8 (4-11)	10 (7-12)	6 (4-8)	2 (1-3.25)	

cART; combined antiretroviral therapy, NNRTI; non-nucleoside reverse transcriptase inhibitor, WAZ; weight for age standard deviations, HAZ; height for age standard deviations, BAZ; Body mass index for age standard deviations, a; Mann-Whitney u test, b; independent samples t-test. Other back bones refer to Tenofovir disoproxil fumarate (TDF) + Emtricitabine (FTC), Abacavir (ABC) + 3TC or Stavudine (D4T) + 3TC. Anemia was defined as hemoglobin (Hg) level is < 11 g/dl for children <5 years old, <11.5 g/dl for children 5–11.9 years old, and <12 g/dl for children >12 years old.

For peer review only

Sociodemographic characteristics of HIV/AIDS-infected children

The median (IQR) age at enrollment to the clinic was 8 years (IQR: 5 -11 years). The median (IQR) of age from 2005 – 2010; >2010-2015, > 2016-2020 were 7 years (IQR: 3-8 years); 8 years (IQR:

6-11 years); and 10 years (IQR: 6.25- 12.75 ears), respectively. In terms of sex, the number of males and females was relatively similar (374 (52.7%) vs. 336 (47.3%)). Majority of participants were from Maekel Zone (72.7%).

Clinical and disease-related characteristics of HIV/AIDS infected children at baseline

Assessment of clinic-level factors indicated that the median CD4 count and percentage at treatment initiation were 274 cells/µl (IQR, 150-442 cells/µl) and 12% (7.4-17%), respectively. In the relevant time frames, the median (IQR) values were 265 cells/µl (151-392.7) cells/µl in 2005 – 2010; 307 cells/µl (137-555) cells/µl in >2010-2015; and 340 cells/µl (200-888) cells/µl in 2016-2020, (p value = 0.012). Similarly, the median (IQR) for CD4 percentages in 2005-2010; >2010-2015; and 2016-2020 were 11.1% (7.1-16.5%); 13.6% (9-21%) and 14.3% (7.5-21.9%), respectively (p-value = 0.007). Overall, and in the following order, 519(73.1%); 264 (37.2%); 16(2.2%) had advanced (STAGE III or IV) disease, anemia or TB at baseline. The proportion of children with WAZ z-score <-2 SD (underweight), HAZ z-score <-2 SD (stunted), and BAZ z-score <-2 SD (undernourished) were 141 (19.9%); 223 (31.4%) and 247 (34.9%), respectively. Notably, and despite the difference in enrollment in the successive time frames, no improvements were observed for these growth parameters (anemia, WAZ, HAZ, and BAZ) (Table 1).

Treatment-related characteristics and outcomes

The preferred cART backbone was AZT+3TC (71.7%). Suboptimal adherence was recorded in 12.3% of patients while the majority (72%) of participants had a history of cART change at least once.

Prevalence of retention and attrition

The overall attrition (death + LTFU), deaths, and LTFU are presented in figure 2. According to this analysis, the overall death rate and LTFU in person years were 2.7 [95% CI 2.1-3.5] /100 PY and 1.2 [95% CI 1-1.5] /100 PY. Attrition at (0 to ≤6 months, >6 to ≤12 months, >12 to ≤24 months, >24 to

Confidential: For Review Only

≤36 months, >36 to ≤48 months, and >48 to ≤60 months of follow-up were 3.6%, 2%, 2.9%, 1.3%, 1.4% and 1.2%, respectively.

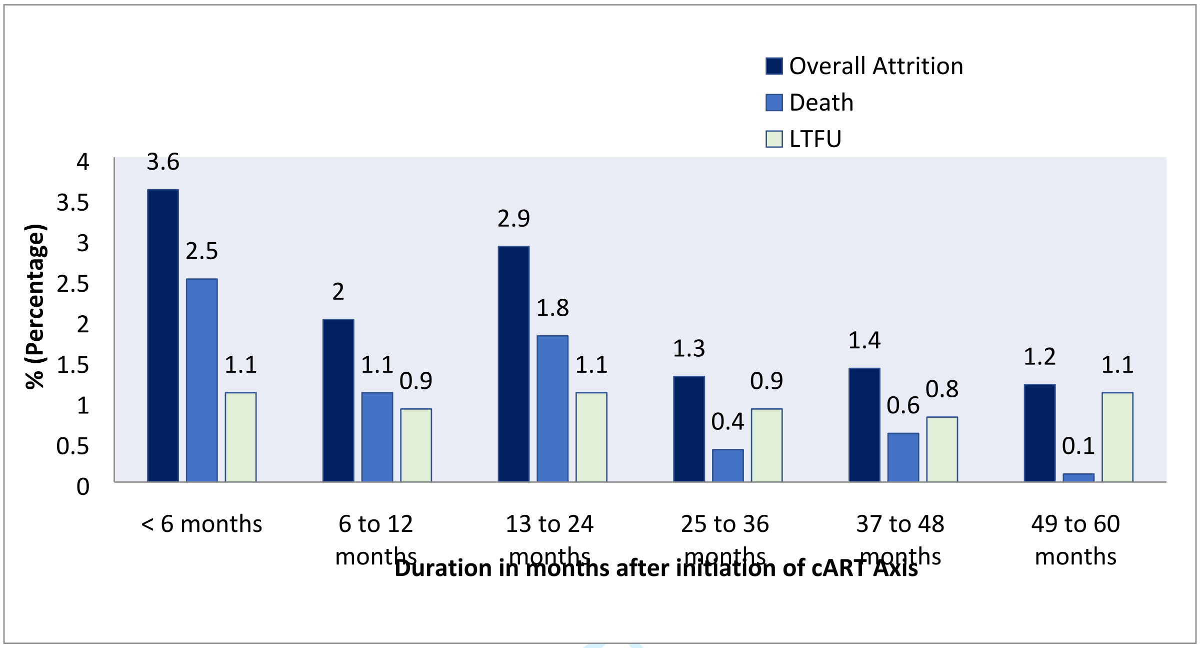

Figure 2. Frequency of attrition, deaths, and LTFU at specified time points within the first 60 months after initiation of cART

Factors Associated with retention and attrition

Out of 710 children included in the study, attrition occurred in 172 (24.2% [95% CI 20-26.3]) patients. The crude incidence and overall survival duration were 3.2 events per 100- person-years (PY) (95% CI 2.7-3.7) and 14 years (95% CI, 13.4-14.8). Duration of follow-up in years for attrition and retention were 4.5 years (IQR: 1-9 years) and 9 years (IQR: 6-11 years), respectively. In terms of proportions, attrition rates were higher in males (104(27.8%) vs 68(20.2%) in females; residence outside Maekel (57(29.4%) vs 115(22.3%); advanced HIV (WHO stage III and IV) 149(28.7) vs 23(12%) for early presentation; presence of anemia (72(27.3%) vs 54(17.3%) in no anemia; WAZ z-score <-2 (underweight) 83(27.5%) vs 27(19.1%) in WAZ z-score >-2; HAZ z-score <-2 (stunting) 122 (26.5%)

For Review Only

vs 42(18.8%) in HAZ z-score > -2; BAZ score <-2 (Under nutrition) (73(29.6%) vs 76(20.1%) in BAZ z-score >-2; AZT+3TC, Else cART backbone 71(35.3%) vs 101(19.9%) in AZT + 3TC; No record of cART change or switching(77(44.8%) vs 92(17.8%). (Table 2)

Cohort Characteristics	Total n (%)	Attrition n (%)	Retention n (%)	P-value ()
**Gender**				
Male	374 (52.7)	104 (27.8)	270 (72.2)	**0.01 (5.5)**
Female	336 (47.3)	68 (20.2)	268 (79.8)	
**Age on enrollment in years, median (IQR)**	6 (3-9)	6 (3-10)	6 (3-9)	0.38[a]
<=5 years	274 (38.6)	62 (22.6)	212 (77.4)	0.09 (4.7)
6-10 years	284 (40)	63 (22.2)	221 (77.8)	
>10 years	152 (21.4)	47 (30.9)	105 (69.10	
**Enrollment Year, Median (IQR)**				0.24[a]
2005-2010	265 (37.3)	111 (23.1)	369 (76.9)	0.23 (2.9)
2011-2015	289 (40.7)	44 (24.4)	136 (75.6)	
2016-2020	156 (22)	17 (34)	33 (66)	
**Address**				
Maekel	516 (72.7)	115 (22.3)	401 (77.7)	**0.049 (3.8)**
Outside Maekel	194 (27.3)	57 (29.4)	137 (70.6)	
**Disease stage**				
Early	191(26.9)	23 (12)	168 (88)	**<0.001 (21)**
Advanced	519 (73.1)	149 (28.7)	370 (71.3)	
**TB- status**				
Symptomatic and under treatment	16 (2.2)	5 (31.3)	11 (68.8)	0.5 (0.4)
Not symptomatic	625 (88)	151 (24.2)	474 (75.8)	
**CD4 count, Median (IQR)**	274 (150-442)	216 (100-371)	287 (163-452)	**<0.001[a]**
**CD4 percentage, Median (IQR)**	12 (7.4-17)	9.9 (6-14.5)	12.5 (8.2-19)	**<0.001[a]**
**Hemoglobin, Mean (±SD)**	11.8(±13.5)	10.7 (9.2-11.8)	11.2 (9.8-12.3)	**<0.001[b]**
Anemia	264 (37.2)	72 (27.3)	192 (72.5)	**0.004 (8.4)**
No Anemia	313 (44.1)	54 (17.3)	259 (82.7)	
**WAZ, median (IQR)**	-2.6 (-3.6--1.7)	-3 (-4.3- -2)	-2.5 (-3.5- --1.6)	**0.001[a]**
WAZ=>-2	302 (42.5)	27 (19.1)	114 (80.9)	**0.05 (3.5)**
WAZ <-2	141 (19.9)	83 (27.5)	219 (72.5)	
**HAZ, median (IQR)**	-2.75 (-3.8--1.7)	-3.2 (-4- -1.9)	-2.5 (3.7- --1.5)	**0.001[a]**
HAZ=>-2	461 (64.9)	42 (18.8)	181 (81.2)	**0.028 (4.8)**
HAZ <-2	223 (31.4)	122 (26.5)	339 (73.5)	
**BAZ, median (IQR)**	-1.58 (-2.8--0.69)	-1.9 (-3.6- -0.7)	-1.5 (-2.6- -0.6)	**0.012[a]**
BAZ=>-2	378 (53)	76 (20.1)	302 (79.9)	**0.007 (7.3)**
BAZ <-2	247 (34.9)	73 (29.6)	174 (70.4)	
**cART backbone**				
AZT+3TC	508 (71.7)	101 (19.9)	407 (80.1)	**<0.001 (18.6)**
Other backbones	201 (28.3)	71 (35.3)	130 (64.7)	
**NNRTI**				
EFV	366 (51.5)	91 (24.9)	275 (75.1)	0.67 (0.1)
NVP	340 (48.4)	80 (23.5)	260 (76.5)	
**Current cART regimen**				
First line cART	633 (89.2)	156 (24.6)	477 (75.4)	0.45 (0.5)
Second line cART	77 (10.8)	16 (20.8)	61 (79.2)	
**Adherence**				
Suboptimal Adherence	91 (12.3)	24 (26.4)	67 (73.6)	0.6 (0.2)
No record	619 (87.2)	148 (23.9)	471 (76.1)	

**cART change**	516 (72.1)	92 (17.8)	424 (82.2)	**<0.001 (50.5)**
No cART change	172 (24.2)	77 (44.8)	95 (55.2)	
Duration of follow up in years, Median (IQR)	8 (4-11)	4.5 (1-9)	9 (6-11)	**<0.001[a]**

Table 2. Characteristics of the study participants stratified by survival outcome in the ONPRH cART treatment center, Asmara, Eritrea (2005–2020).

cART; combined antiretroviral therapy, NNRTI; nonnucleoside reverse transcriptase inhibitor, WAZ; weight for age standard deviations, HAZ; height for age standard deviations, BAZ; Body mass index for age standard deviations, a; Mann-Whitney u test**,** b; independent samples t-test. Other backbones: Abacavir + Lamivudine [ABC/3TC] Stavudine + lamivudine [d4T+3TC] Tenofovir + Emtricitabine [TDF+FTC]
Anemia was defined as hemoglobin (Hg) level is < 11 g/dl for children <5 years old, <11.5 g/dl for children 5–11.9 years old, and <12 g/dl for children >12 years old.

Confidential: For Review Only

Kaplan-Meier analysis for Attrition incidence

Kaplan-Meier curves were constructed to compare the cumulative incidence of attrition by gender, address, cohort year, AHD, initial cART backbone, and record of cART change (See Figure 3 and Table 3). Overall, being male was associated with shorter survival (13 (95% CI, 2-14 years) vs 13.8(95% CI, 13–14 years)) (log rank test, p = 0.008) (figure 2a). Significantly shorter mean survival duration was observed with increasing age at baseline: <=5 years: 15.1 (14.2-16) years' vs 6-10 years: 11.7(11.1-12.2 years) and >10 years: 8.4(7.43-12.2 years) years (log rank test p < 0.001). Children from outside Maekel were also found to have a significantly shorter duration of survival in the clinic mean (11 years (95% CI, 10-11.7 years)) compared to those from Maekel (14.5 years (95% CI, 13.8-15.4 years)) (log rank test p = 0.002 (Figure 2b). Late enrollment calendar years were also associated with shorter mean survival duration (2005-2009: 14.8 years (95% CI, 14–15.5 years) vs 2010-2014: 7.5 years (95% CI, 7-8 years) vs 2015-2020: 3.1 years (95% CI, 2.7-3.5 years) (log rank test p = 0.008 (figure 2c). Children with AHD at presentation had a significantly shorter mean survival time in ONPRH (13 years (95% CI 12.3-14)) compared to 15 years (95% CI, 14.2-15.7 years) for those without. The survival time for AZT+3TC, else group 11.3 years (95%CI, 9.8–12.8 years) was significantly shorter compared to the AZT+3TC group 14 years (95%CI, 13.4–14.5 years) (log rank test p < 0.001). (Figure 2d). Moreover; children with a record of cART change had significantly longer mean survival time in the clinic 13 years (95% CI 13-14 years) as compared (7.5 years (95%

CI, 6.6-8.4 years) (log rank test $p < 0.001$). Others associated with shorter survival were: anemia at ART initiation (Hemoglobin < 8 g/dl) ($p = 0.032$), WAZ score <-2 12 years (11.4-12.8 years); HAZ score <-2 (12.7 years (12-13.4 years); BAZ score <-2 (12 years (11-13 years).

Confidential: For Review Only

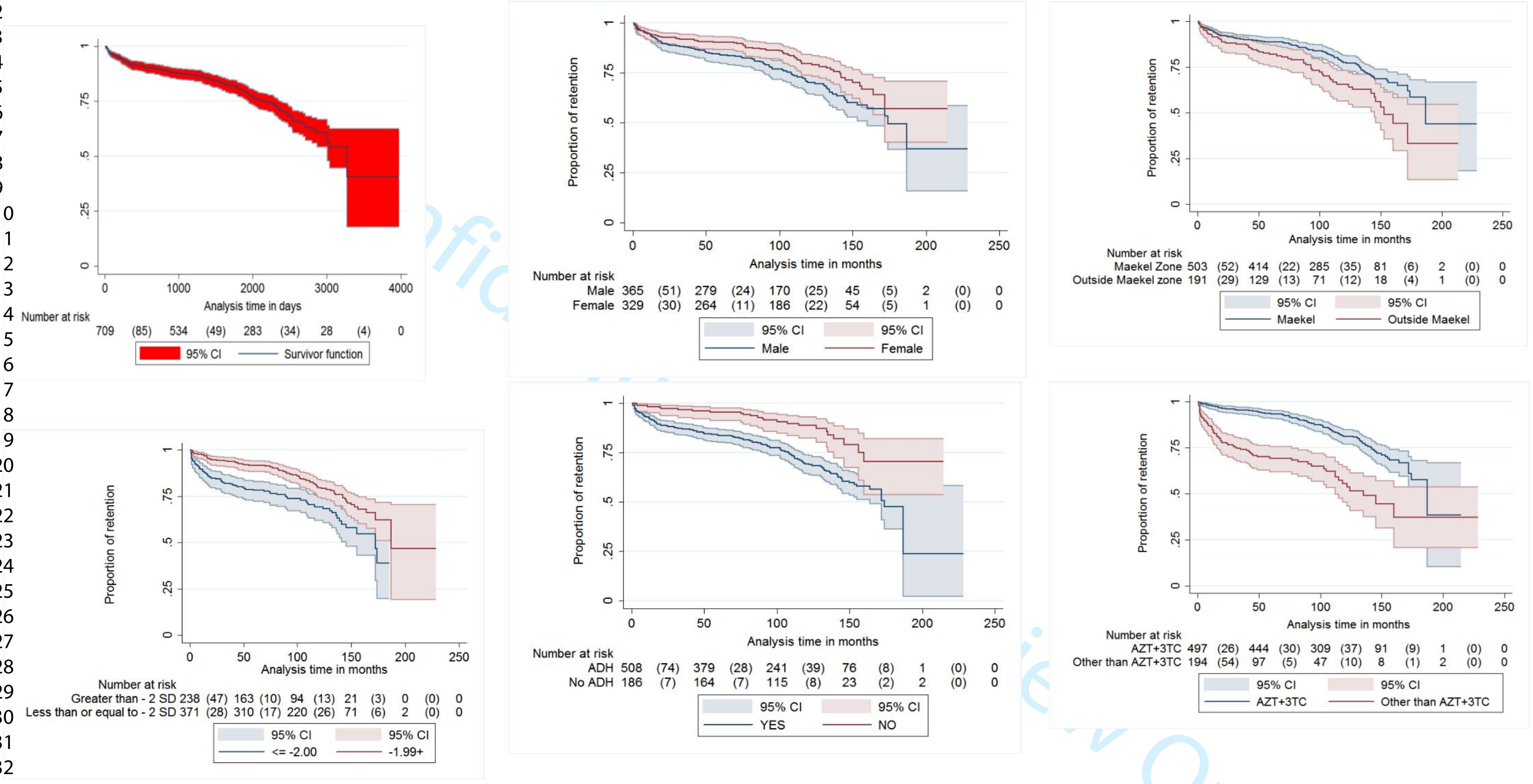

Figure 3. Kaplan-Meier cumulative incidence of attrition unadjusted curves for children followed in ONPRH from 2005- 2020

Table 3. Crude incidence, rate of attrition, and Kaplan-Meier survival estimates of children followed in the ONPRH cART treatment center, Asmara, Eritrea (2005–2020).

Cohort Characteristics	Incidence of attrition per 100 person years (95% CI)	Mean survival duration in years, 95% CI	P-value (log-rank)
**Total**	3.2 (2.7-3.7)	14.1 (13.4-14.8)	
**Gender**			
Male	3.8 (31.-4.6)	13.2 (2.1-14.3)	**0.008 (6.9)**
Female	2.5 (2-3.2)	13.8 (13.1-14.4)	
**Age in years at enrollment**			
<=5 years	2.5 (1.9-3.2)	15.1 (14.2-16)	**<0.001 (36.8)**
6-10 years	2.8 (2.2-3.6)	11.7 (11.1-12.2)	
>10 years	6.7 (5-8.9)	8.4 (7.43-9)	
**Address**			**0.002 (9.8)**
Maekel	2.7 (2.3-3.3)	14.6 (13.8-15.4)	
Outside Maekel	4.6 (3.5-5.9)	10.9 (10-11.7)	
**Disease Stage at presentation**			**<0.001 (22)**
Early	1.4 (0.9-2.1)	15 (14.2-15.7)	
Advanced	3.9 (3.3-4.6)	13.2 (12.3-14)	
**TB-Status**			0.2 (1.5)
Symptomatic and under treatment	3.1 (2.6-3.7)	9.9 (6.9-12.8)	
Not symptomatic	6.5 (2.7-15.8)	14 (13.3-14.8)	
**Hemoglobin**			**0.004 (8.3)**
Anemia	2.2 (1.7-2.9)	11.5 (11-12)	
Normal	3.7 (2.9-4.7)	12.8 (12-13)	
**WAZ**			**0.014 (6)**
WAZ=>-2	2.1 (1.48-3.1)	13 (12.4-13.7)	
WAZ <-2	3.5 (2.8-4.4)	12 (11.4-12.8)	
**HAZ**			**0.019 (5.4)**
HAZ=>-2	2.3 (1.7-3.2)	14.9 (13.7-16)	
HAZ <-2	3.5 (3-4.2)	12.7 (12-13.4)	
**BAZ**			**<0.001 (12.4)**
BAZ=>-2	2.4 (1.9-3)	15 (14-15.9)	
BAZ <-2	4.4 (3.5-5.5)	12 (11-13)	
**Initial cART backbone**			**<0.001 (54.5)**
AZT+3TC	2.3 (1.9-2.8)	13.9 (13.4-14.5)	
Other backbones	7.1 (5.6-9)	11.3 (9.8-12.8)	
**NNRTI**			0.9 (0.015)
EFV	3.2 (2.6-3.9)	13 (12.3-13.7)	
NVP	3 (2.5-3.9)	14.2 (13.3-15.2)	
**Current cART regimen**			
First line cART	3.34 (2.8-3.9)	12.9 (12.3-13.4)	0.15 (2)
Second line cART	2.2 (1.3-3.7)	15.7 (14.3-17)	
**Adherence**			0.68 (0.16)
Suboptimal Adherence	2.9 (1.9-4.3)	12.6 (11.8-13.4)	
No record	3.2 (2.7-3.7)	14.3 (13.6-15)	
**cART Change**			**<0.001 (136)**
cART change	1 (0.84-1.3)	13.6 (13-14)	
No cART change	2 (1.7-2.5)	7.5 (6.6-8.4)	

cART; combined antiretroviral therapy, NNRTI; non-nucleoside reverse transcriptase inhibitor, WAZ; weight for age standard deviations, HAZ; height for age standard deviations, BAZ; Body mass index for age standard deviations. Other backbones: Abacavir + Lamivudine [ABC/3TC] Stavudine + lamivudine [d4T+3TC] Tenofovir + Emtricitabine [TDF+FTC]. Anemia was defined as hemoglobin (Hg) level is < 11 g/dl for children <5 years old, <11.5 g/dl for children 5–11.9 years old, and <12 g/dl for children >12 years old.

Confidential: For Review Only

Independent predictors of attrition rate in children enrolled in ONPRH cART treatment and follow-up clinic

The unadjusted and adjusted hazard ratios for variables associated with attrition in 701 patients are given in Table 4. In the univariate Cox proportional hazards model, increased risk of attrition was associated with being male (UHR=1.4, 95% CI 1–2, p=0.01); Residence outside Zoba Maekel (UHR=1.6, 95% CI 1.2-2.2, p=0.002); WHO BAZ score <-2 (UHR=1.8, 95% CI 1.3-2.5, p=0.001); WHO HAZ score (UHR=1.5, 95% CI 1-2.1, p=0.02); presence of anemia (UHR=1.7, 95% CI 1.7 1.2-2.4, p=0.005); advanced disease (WHO III or IV) at presentation (UHR=2.9, 95% CI 1.8-4.3, p<0.001); Initiation on AZT+3TC or other cART backbones (UHR=3, 95% CI 2.2-4.2, p<0.001). In contrast, reduced risk of attrition was associated with cART changes (UHR=0.16, 95% CI 0.12-0.2, p<0.001).

In the adjusted Cox proportional hazards model, independent predictors of attrition were male gender (AHR=1.6, 95% CI 1–2.4, p=0.01), Residence outside Zoba Maekel (AHR=1.5, 95% CI 1–2.3, p=0.02); Later enrollment years (2010-2015: AHR=3.2, 95% CI 1.9-5.3, p<0.001) (>2015: UHR=6.1, 95% CI 3-12.2, p<0.001); BAZ < -2 (AHR= 1.4, 95% CI 0.9-2.1, p=0.05); advanced HIV disease (WHO III or IV) at enrollment (AHR= 2.2, 95% CI 1.2-3.9, p=0.004); Initiation of AZT+3TC or other cART backbones (UHR=2, 95% CI 1.2-3.2, p=0.005). In contrast, a reduced likelihood of attrition was observed in children with a record of cART changes (UHR=0.2, 95% CI 0.15-0.4, p<0.001). Table 4.

https://mc.manuscriptcentral.com/bmjpo

Confidential: For Review Only

Table 4, Cox proportional hazards of attrition among HIV-infected children and adolescents at ONPRH cART treatment center, Asmara, Eritrea (2005–2020).

Cohort Characteristics	Unadjusted HR(95%CI)	p-value	adjusted HR(95%CI)	p-value
**Gender**				
Female	1	0.01	1	0.01
Male	1.4 (1-2)		1.6 (1-2.4)	
**Age at enrollment**				
<= 5	1		1	
6-10	1.3 (0.9-1.8)		1.1 (0.7-1.6)	
>10	3.3 (2.2-5)		1.5 (0.9-2.5)	
**Address**				
Maekel	1	0.002	1	0.02
Outside Maekel	1.6 (1.2-2.2)		1.5 (1-2.3)	
**Year of Enrollment**				
< 2010	1		1	
2010-2015	1.9 (1.3-2.7)		3.2 (1.9-5.3)	<0.001
>2015	4.2 (2.5-7.2)		6.1 (3-12.2)	<0.001
**BMI for age, z-score**				
**>= 2**	1	0.001	1	0.03
**< -2**	1.8 (1.3-2.5)		1.4 (0.9-2.1)	
**Height for age, z-score**				
**>= 2**	1	0.02		
**< -2**	1.5 (1-2.1)			
**Hemoglobin**				
Normal	1	0.005		
Anemia	1.7 (1.2-2.4)			
**Advanced disease at presentation**				
No	1	<0.001	1	0.004
Yes	2.9 (1.8-4.3)		2.2 (1.2-3.9)	
**TB status**				
Not symptomatic	1	0.23		
Took Anti-TB	1.8 (0.8-5)			
**cART Backbone**				
AZT+3TC	1	<0.001	1	0.005
Other backbones	3 (2.2-4.2)		2 (1.2-3.2)	
**NNRTI**				
EFV	1	0.9		
NVP	0.95 (0.7-1.2)			
**cART changes**				
No	1	<0.001	1	<0.001
Yes	0.16 (0.12-0.2)		0.2 (0.15-0.4)	
**Current cART regimen**				
**First line cART**	1	0.16		
**Second line cART**	0.68 (0.4-1.1)			
**Suboptimal adherence**				
No	1	0.2		
Yes	1.1 (0.7-1.7)			

cART; combined antiretroviral therapy, NNRTI; non-nucleoside reverse transcriptase inhibitor, WAZ; weight for age standard deviations, HAZ; height for age standard deviations, BAZ; Body mass index for age standard deviations. Only factors significant at univariate or borderline were considered into multivariate. Other backbones: Abacavir + Lamivudine [ABC/3TC] Stavudine + lamivudine [d4T+3TC] Tenofovir + Emtricitabine [TDF+FTC].

Confidential: For Review Only

Discussion

In general terms, this study, the first of its kind in Eritrea, uncovered a number of interesting results. One of the most striking results was the steep decline in enrollment – 2005-2010 (480(67.6%); 2011-2015 (180(25.4%); 2016-2020 (50(7.0%). Multiple interpretations can be invoked to explain the observed results. On the one hand, the average enrollment of 10 children per year to HIV care services in a city with a population > 500 000 creates the impression that our study has underestimated the absolute number of HIV-positive children who are eligible for treatment. Ample data support the idea that pediatric enrollment of adolescents and young adults (AYA) living with HIV is suboptimal in most countries in SSA [14]. However, in Eritrea, the utilization of prenatal services by pregnant mothers has progressively improved in recent years and is now better overall than those of most countries in SSA[8] and linkage to care (post-test counseling of HIV infected mothers, linkage to HIV/AIDS care points, initial clinical and laboratory evaluation, and strict adherence to a "Test and Start" treatment paradigm[15]) is prompt. Therefore, it's our belief that the number of unenrolled infants is marginal.

Altogether, available evidence supports the idea that effective PMTCT services can reduce mother-to-child transmission (MTCT), thereby reducing incident pediatric HIV infection. An alternative explanation of the observed outcome may be connected to the dramatic reduction in HIV prevalence in the general population - ~ 2.4% in 2003 to 0.36% (~14 000) at the end of 2019 (Antenatal Care 2019 Preliminary Report). Whatever the case, we can cautiously conclude that the data highlights the success of HIV programs in Asmara, Eritrea – particularly the idea that effective PMCTC programs can drastically reduce HIV infection in newborns.

Positives aside, our data highlighted specific concerns: attrition in the first 24 months following cART initiation was largely due to premature death; late presentation is frequent; the median age

Confidential: For Review Only

at cART initiation has increased; and attrition rate was constant in the 2005-2010; 2011-2015; 2016-2020 period. The fact that the lower threshold for child and infant cART eligibility in 2011, guidelines have failed to lower the median age at initiation was an unexpected [16 17]. Putting adequate and holistic interventions in place requires good data. Future intervention research is urgently needed to improve. More research is needed to understand how to best integrate clinic and community-based responses to discrimination, internalized stigma, and ART no adherence among adolescents living with HIV. Perhaps the most worrisome finding in this study is that attrition hasn't declined over the years, in fact patients enrolled after 2010 were three times more likely to experience attrition while those enrolled after 2015 were six times as likely. This is an alarming finding despite the decline in the number of patients enrolled across the three-year categories. The issue has to be addressed seriously and further probes should be conducted to identify the difficulties encountered by patients to stay in care to bolster retention rates in the clinic.

These explanations, while plausible; are entirely speculative. Therefore, a solution to this quandary requires data on multiple domains in the HIV care cascade – children/infants diagnosed with HIV in PMCTs, hospitals/clinics, or VCTs across Asmara within the study period; the proportion linked to HIV care and initiated on cART; and the proportion retained in HIV care programs. Data on the proportion of patients who were eligible for cART (particularly adolescents and young adults (AYA) living with HIV) based on previous guidelines; but were not placed on treatment is equally important. Unfortunately, this information is hard to access owing to a variety of complex reasons - lack of linked data across HIV points of service in Asmara (or across Eritrea); poor patient tracking/tracing systems within the country; and possible inconsistencies in appointment schedules. Therefore, at the very least, our findings provide further support for the need for linked databases across HIV/AIDS care points within Eritrea. The importance of enhanced outreach and

https://mc.manuscriptcentral.com/bmjpo

Confidential: For Review Only

intensive case-finding for LTFU; as standard components of HIV care programs in Eritrea is equally evident.

Despite the broad scale-up of HIV services and expanded cART coverage in SSA, the proportion of children receiving treatment remains low. More importantly, attrition rate among those receiving care were disproportionately high 6. In this study, overall attrition (LTFU + death) rate was 24.2% [95% CI 20-26.3]). The overall attrition rate at <6, 6-, 12-, 25-, 36- and 48 months were 3.6%, 2%, 2.9%, 1.3%, 1.4%, and 1.2%, respectively. These findings demonstrate a much lower prevalence of attrition compared to similar studies from SSA. For instance, a study conducted in Nigeria reported a 6-, 12-, 24-, and 36-months attrition rate of 20.4, 27.7, 34.3, and 37.5%, respectively [18]. Similarly, a meta-analytical study by Carlucci reported a 6- and 36 months attrition of 19% (95% CI: 16–22%) and 23% (95% CI: 18–28%), respectively [6]. However, our finding of high attrition rate in the first 6 months of treatment has been reported by multiple investigators [6 19].

Similar to our study, the preponderance of attrition in the first 6 months was associated with high mortality rates - 11.9 (7.6–16.3) deaths/100 PY at 3 months and 8.7 (6.0–11.41) deaths/100 PY at 6 months in Zambia [19]; 10.3 (95% CI, 6.9–15.2) deaths/100 PY) at 6 months and 0.58(95% CI, 0.32–1.05) deaths/100 PY thereafter, in Thailand [20]. In the multicountry treated Asia Pediatric HIV Observational Database; mortality decreased from 10.2 (95% CI: 7.5-13.7) deaths/100 PY in the first 3 months to 4.2 (95% CI, 2.6-6.8) deaths/100 PY) in the 4th and 6th months [19]. In other words, high mortality in the first six months is common in LMICs. The high mortality in the first 6 months of enrollment in care has largely been attributed to late presentation – WHO stage III or IV; chronic diarrhea and weight for height z-score, < -2 (severe wasting), among others. Observational studies suggest that the first 6 months after treatment initiation is the most vulnerable period for HIV positive children in SSA [6]. Studies repeatedly cite the need for context-specific mitigation

Confidential: For Review Only

(phone/Short messaging services (SMS)-based messaging/reminder; improved linkage of child and maternal HIV/AIDS services; and enhanced community outreach and patient tracing; intensive case management, drug adherence counseling, among others)[6] directed at maximization of retention at this stage.

Furthermore, analysis of incidence of attrition demonstrated a rate of 3.2 (95% CI 2.7-3.7)/100 PY. This incidence estimate is lower when compared to studies conducted in Ethiopia (8.36[95% CI: 7.12−9.80]/100 PY) [21]; Zimbabwe (11.8, [95% CI:11.0–12.7]/100 PY [22]; Nigeria (10.8/100 PY) [18]; 3.4[95% CI: 2.9-4] /100 PY [19]; Myanmar (4 [95% CI: 3 – 4]/100 PY) [17] and 6.4/100 PY [12]; Kenya (23.1/100 PY) [23]; and South Africa (26.4/100 PY) [Bock]. In contrast, the incidence of attrition was higher than what was reported in Mozambique (2.2/100 PY) [24]; Thailand (2.9 (95% CI, 2.7-3.2)/100 PY [25] and in a Dutch cohort 0.3/100 PY [26]. The variation/discrepancy in incidence between countries in SSA is generally attributed to variations in access and quality of healthcare services; health seeking behaviors between populations [27]; heterogeneity in methodology and definition of outcomes, among others. Finally, we have to highlight the possibility that the LTFUs registered in our study may actually be attributable in large part to undocumented mortality and not undocumented transfers, death, or disease of the caregiver or disengagement from care. Therefore, the possibility that our studies underestimated mortality should not be overlooked.

In many respects, the predictors of attrition identified in this study are largely similar to those uncovered in other studies in the region. For instance, evidence from multiple studies in the region have indicated that WHO BAZ < -2 (under-nutrition) and BMI for age z-score < -2 are baseline hazards for cART attrition[9 11 22]. Although the presence of anemia at baseline was associated with an increased hazard of attrition (1.7(1.2-2.4)), the association was attenuated in the adjusted multivariable model. This suggests the presence of confounders. Nevertheless, the conjunction between these parameters should not be ignored. Mechanistically, data suggest that the co-

Confidential: For Review Only

presence of severe malnutrition (SAM) + HIV is associated with perturbations in the gut microbiome, accelerated depletion of gut-resident $CD4^+$ T lymphocyte cell populations and aberrant mucosal immune responses that can accelerate microbial translocation. In turn, this can trigger systemic immune activation, immune exhaustion, impaired response to cART, and a concomitant increase in morbidity and mortality due to increased incidence and severity of concurrent infection [28]. Indeed, data suggest that morbidity and mortality in children on cART is largely due to tuberculosis (TB), pneumocystis pneumonia (PCP), severe bacterial infections, malnutrition/wasting, hematological disturbances, and immune reconstitution inflammatory syndrome (IRIS) [29].

Elsewhere, others have argued that HIV+SAMS represents a distinct disease stratum/ phenotype with worse prognosis and impaired immune recovery [30]. Importantly, the clinical management of this class of patients is extremely challenging [13]. Highlighting this point, some investigators have suggested that rapid initiation (within 1 week) of cART in children with SAM or WHO stage III or IV disease may be counterproductive and that treatment in these patients should be delayed until the patient is stabilized [29]. Therefore, in addition to the previously highlighted interventions, additional efforts must be made to provide enhanced nutritional monitoring, and, where applicable, support; to new enrollees.

Based on the foregoing, the observed connection between advanced HIV disease (III or IV) at enrollment and attrition rate was anticipated (aHR = 2.2 (95% CI: 1.2-3.9)). The evidence is incontrovertible - late presentation remains a challenge for pediatric HIV/AIDS programs in SSA. For example, Mutanga et al., demonstrated an aHR of 4.8 (95% CI: 2.3–10) times in stage 4 patients compared to WHO stage 1 and 2 [19]. Reports from Zimbabwe [22], Nigeria [18], and Myanmar [17] have largely corroborated these findings. The possible explanations behind this outcome relates

Confidential: For Review Only

to the possibility that late-stage disease is associated with SAM and debilitating co-infections that may compromise treatment – diarrhea and may compromise cART bioavailability, among others.

Additional factors, unique to this setting, which were associated with attrition, included male gender and residence outside Asmara. In general, data on the relationship between sex and attrition is mixed, with most studies reporting a null relationship while others skew towards either sex. For example, Zanoni *et al.* noted that female sex was associated with high mortality in their cohort [9]. However, a systematic review of eight studies from SSA and two studies from Asia by Mugglin et al. reported a null relationship [31]. Others have highlighted the paucity of gender-disaggregated retention/attrition data in ALHIV for adolescents in SSA [32]. Overall, empirical data on the relationship between sex and attrition are notable for their inconsistency. We believe that these inconsistencies and paradoxical results in cohorts across SSA mirrors the disparate context-specific influences at play.

In Asmara, Eritrea, the observed association may be linked to stigma, multi-faceted discrimination, and the possibility that males and females (particularly ALHIV) experience these factors differently. Previous studies have demonstrated that stigma, discrimination, and ridicule from friends/peers may compromise regular follow-up in ALHIV. Stigma may also explain why parents or guardians may prefer to enroll children in distant cART facilities and not in facilities nearest to them. In turn, this practice can impose unnecessary out-of-pocket costs (transport). Indeed, empirical data suggests that geographical access to the clinic and transportation costs are major barriers to retention in cART clinics in LMIC. Altogether, assessing and addressing context-specific reasons for possible sex disparities in attrition; stigma or preference for distant cART facilities is an area for future research. Adoption of innovative models/packages of care to address unique ALHIV needs is equally evident.

https://mc.manuscriptcentral.com/bmjpo

Confidential: For Review Only

Lastly, in the multivariable proportional hazard's regression, the adjusted hazards of attrition were highest in children without a record of cART changes or on other cART backbones - ABC/3TC; d4T+3TC or TDF+FTC. The associations highlight a major systematic weakness in cART clinics, quality across SSA – the limited diversity of cART options and delays in switching to more potent second line regimens after detection of therapy failure – virological failure. Retention of patients on a failing regimen is known to augment mortality hazards or emergence of drug resistance. Therapy failure, particularly in the recent calendar, may actually account for the observed increase in attrition rates. Silent transfer to nearby health facilities – a consequence of enhanced decentralization of cART services in recent years; this is another plausible explanation of the high attrition in recent calendar years. More plausible still is the possibility that HIV-positive children recruited in the test-and-treat era are generally less sick: this outcome has been associated with low-risk perception and increased likelihood of attrition [33].

Strength and limitations of the study

This study, the first of its kind in Eritrea, has several strengths. First, the use of routinely collected information from the largest pediatric cART clinic in Eritrea, rather than data collected in a research setting; this can be a useful gauge of real-world program successes and weaknesses. Importantly, the data can identify critical gaps and opportunities for context-specific interventions aimed at improvement of program quality and outcomes. Furthermore, the study duration was sufficiently long (15 years) and the sample size was large. This permitted us to undertake multiple subgroup analysis and to make reasonable conclusions/generalizations about the program. Regardless, our evaluation highlighted a number of limitations. First, retrospective designs suffer from multiple constrains – missing data, among others. Missing or unstructured collection of information ($CD4^+$ cell counts or viral load data) and lack of data on causes of death and outcomes

Confidential: For Review Only

in children characterized as LTFU are particular examples. In the absence of tracing information, mortality estimate in this cohort are unclear.

Conclusion

In general terms, this study, the first of its kind in Eritrea, uncovered a number of interesting results. One of the most striking results was the steep decline in enrollment – 2005-2010 (480(67.6%); 2011-2015 (180(25.4%); 2016-2020 (50(7.0%)). It's our opinion that the steep decline in enrolment may potentially highlight the fact that effective PMTCT services in prenatal clinics can reduce MTCT thereby reducing incident pediatric HIV infection. After 5364 person-years follow-up, attrition occurred in 172 (24.2% [95% CI 20-26.3]) patients. The crude incidence rate of attrition was 3.2 events (95% CI 2.7-3.7)/100 person-years (PY) - mortality rate and lost to follow-up (LTFU) were 2.7 [95% CI 2.1-3.5] /100 PY and 1.2 [95% CI 1-1.5] /100 PY, respectively. In other words, attrition in the first 24 months was largely due to death. Despite the large number of late presentations, the incidence estimate is lower when compared to studies conducted in multiple LMICs. Beyond these encouraging results, multiple concerns were highlighted – late presentation; high mortality and the first 24 months after initiation of cART; and a constant attrition rate in the 2005-2010; 2011-2015; 2016-2020 periods among others. In general, these factors point at possible gaps in program quality. This possibility was reinforced in a separate analysis (Cox proportional hazards model) which demonstrated that independent predictors of attrition were later enrollment years; BAZ < -2; advanced HIV disease (III or IV) at enrollment; initiation of AZT+3TC or other cART backbones, and residence outside Zoba Maekel. The need to focus on the quality and outcomes of cART in Eritrea is adequately highlighted in this study. From a programmatic point of view, our findings provide further support for the need for linked databases across the HIV/AIDS care cascades within Eritrea. The importance of enhanced outreach and intensive case-finding/tracing for LTFU; as standard components of HIV/AIDS care programs

Confidential: For Review Only

in Eritrea is equally evident. Finally, putting comprehensive clinical and programmatic interventions in place will require good data; unfortunately, large data gaps were evident in this program. Therefore, research should be prioritized.

Declaration

Ethical approval and consent to participate

Ethical approval for this study was obtained from the Eritrean Ministry of Health (MOH) Research Ethical Committee. Information on the maintenance of data confidentiality and integrity was also provided. As the study is based on patients' clinical card records, consent for data access was waived by the ethical committee in place of the patients. This study conforms to the principles outlined in the Declaration of Helsinki.

Consent for publication

Consent for publication is also waived by the ethical committee in place of patients as the data is secondary.

Availability of data and materials

The datasets supporting the conclusions of this article are available from the corresponding author on reasonable request.

Conflict of Interest

The authors have no conflict of interest to declare in this study.

Financial support

The study hasn't received any funding.

Confidential: For Review Only

Authors' contributions

Conceptualization: Samuel Tekle Mengistu

Data curation: Samuel Tekle Mengistu, and Ghirmay Ghebrekidan,

Formal analysis: Samuel Tekle Mengistu, Oliver Okoth Achila and Samuel Tekle

Investigation: Samuel Tekle Mengistu, Mohammed Elfatih Hamida and Oliver Okoth Achila.

Methodology: Samuel Tekle Mengistu, Ghirmay Ghebrekidan, and Aron Rezene

Project administration: Samuel Tekle Mengistu, Mahmud Mohammed Idris, Tsegereda Gebrehiwot Tikue, Mohammed Elfatih Hamida, and Oliver Okoth Achila

Writing – original draft: Samuel Tekle Mengistu, and Oliver Achila.

Writing – review& editing: Samuel Tekle Mengistu, Ghirmay Ghebrekidan Ghebremeskel, Oliver Achila, Mahmud Mohammed Idris, Tsegereda Gebrehiwot Tikue, Mohammed Elfatih Hamida, and Oliver Okoth Achila.

Acknowledgment

We are grateful to National Communicable Disease Control Division, Eritrean Ministry of Health, and ART Health Management Information System (HMIS) developers and technicians. Our sincere appreciation goes to Dr. Araia Berhane, Dr. Fanus Yemane, Dr. Simon Tesfay, Dr. Yonathan Tesfalidet, and Mr. Thomas Asfaha.

Reference

Confidential: For Review Only

1. Global H. AIDS statistics–2019 fact sheet. UNAIDS. *URL: http://www. unaids org/sites/default/files/media_asset/UNAIDS_FactSheet_ru pdf* 2020

2. Levi J, Raymond A, Pozniak A, et al. Can the UNAIDS 90-90-90 target be achieved? A systematic analysis of national HIV treatment cascades. *BMJ global health* 2016;1(2):e000010.

3. Ng'eno B, Mwangi A. Burden of HIV infection among children aged 18 months to 14 years in Kenya: results from a nationally representative population-based cross-sectional survey. *Journal of acquired immune deficiency syndromes (1999)* 2014;66(Suppl 1):S82.

4. Desmonde S, Tanser F, Vreeman R, et al. Access to antiretroviral therapy in HIV-infected children aged 0–19 years in the International Epidemiology Databases to Evaluate AIDS (IeDEA) Global Cohort Consortium, 2004–2015: a prospective cohort study. *PLoS medicine* 2018;15(5):e1002565.

5. Bain LE, Nkoke C, Noubiap JJN. UNAIDS 90–90–90 targets to end the AIDS epidemic by 2020 are not realistic: comment on "Can the UNAIDS 90–90–90 target be achieved? A systematic analysis of national HIV treatment cascades". *BMJ global health* 2017;2(2):e000227.

6. Carlucci JG, Liu Y, Clouse K, et al. Attrition of HIV-positive children from HIV services in low-and middle-income countries: a systematic review and meta-analysis. *AIDS (London, England)* 2019;33(15):2375.

7. Phelps BR, Ahmed S, Amzel A, et al. Linkage, initiation and retention of children in the antiretroviral therapy cascade: an overview. *AIDS (London, England)* 2013;27(0 2):S207.

8. Division CDC. ERITREAN ANTIRETROVIRALTHERAPY GUIDELINES. Asmara, Eritrea: Ministry of Health, 2017.

Confidential: For Review Only

9. Zanoni BC, Phungula T, Zanoni HM, et al. Risk factors associated with increased mortality among HIV infected children initiating antiretroviral therapy (ART) in South Africa. *PloS one* 2011;6(7):e22706.

10. Fenner L, Brinkhof MW, Keiser O, et al. Early mortality and loss to follow-up in HIV-infected children starting antiretroviral therapy in Southern Africa. *Journal of acquired immune deficiency syndromes (1999)* 2010;54(5):524.

11. Brophy JC, Hawkes MT, Mwinjiwa E, et al. Survival Outcomes in a Pediatric Antiretroviral Treatment Cohort in Southern Malawi. *PLoS One* 2016;11(11):e0165772. doi: 10.1371/journal.pone.0165772 [published Online First: 2016/11/05]

12. Htun T, Kyaw K, Aung T, et al. Attrition during pre-ART and ART time periods among adolescents enrolled in Integrated HIV Care Programme in Myanmar, 2005–2017. *Epidemiology & Infection* 2019;147

13. Organization WH. Guidelines for managing advanced HIV disease and rapid initiation of antiretroviral therapy, July 2017. 2017

14. Lamb MR, Fayorsey R, Nuwagaba-Biribonwoha H, et al. High attrition before and after ART initiation among youth (15-24 years of age) enrolled in HIV care. *Aids* 2014;28(4):559-68. doi: 10.1097/qad.0000000000000054 [published Online First: 2013/10/01]

15. Rosen S, Fox MP, Larson BA, et al. Accelerating the uptake and timing of antiretroviral therapy initiation in sub-Saharan Africa: an operations research agenda. *PLoS medicine* 2016;13(8):e1002106.

16. Organization WH. Antiretroviral therapy of HIV infection in infants and children: towards universal access: recommendations for a public health approach-2010 revision: World Health Organization 2010.

Confidential: For Review Only

17. Minn AC, Kyaw NTT, Aung TK, et al. Attrition among HIV positive children enrolled under integrated HIV care programme in Myanmar: 12 years cohort analysis. *Global health action* 2018;11(1):1510593.

18. Onubogu CU, Ugochukwu EF. A 17 year experience of attrition from care among HIV infected children in Nnewi South-East Nigeria. *BMC Infectious Diseases* 2021;21(1):1-11.

19. Mutanga JN, Mutembo S, Ezeamama AE, et al. Long-term survival outcomes of HIV infected children receiving antiretroviral therapy: an observational study from Zambia (2003–2015). *BMC public health* 2019;19(1):1-12.

20. Collins IJ, Jourdain G, Hansudewechakul R, et al. Long-term survival of HIV-infected children receiving antiretroviral therapy in Thailand: a 5-year observational cohort study. *Clinical infectious diseases* 2010;51(12):1449-57.

21. Demeke Dejen DJ, Yeshanew F, Fentaw Z, et al. Attrition and Its Predictors Among Adults Receiving First-Line Antiretroviral Therapy in Woldia Town Public Health Facilities, Northeast Ethiopia: A Retrospective Cohort Study. *HIV/AIDS (Auckland, NZ)* 2021;13:445.

22. Makurumidze R, Mutasa-Apollo T, Decroo T, et al. Retention and predictors of attrition among patients who started antiretroviral therapy in Zimbabwe's national antiretroviral therapy programme between 2012 and 2015. *PloS one* 2020;15(1):e0222309.

23. Hassan AS, Mwaringa SM, Ndirangu KK, et al. Incidence and predictors of attrition from antiretroviral care among adults in a rural HIV clinic in Coastal Kenya: a retrospective cohort study. *BMC Public Health* 2015;15(1):1-9.

24. Decroo T, Koole O, Remartinez D, et al. Four-year retention and risk factors for attrition among members of community ART groups in T ete, M ozambique. *Tropical Medicine & International Health* 2014;19(5):514-21.

Confidential: For Review Only

25. Teeraananchai S, Kerr SJ, Puthanakit T, et al. Attrition and mortality of children receiving antiretroviral treatment through the universal coverage health program in Thailand. *The Journal of pediatrics* 2017;188:210-16. e1.

26. Cohen S, Smit C, van Rossum AM, et al. Long-term response to combination antiretroviral therapy in HIV-infected children in the Netherlands registered from 1996 to 2012. *Aids* 2013;27(16):2567-75.

27. Bimer KB, Sebsibe GT, Desta KW, et al. Incidence and predictors of attrition among children attending antiretroviral follow-up in public hospitals, Southern Ethiopia, 2020: a retrospective study. *BMJ paediatrics open* 2021;5(1)

28. Desai SN, Landay AL. HIV and aging: role of the microbiome. *Current Opinion in HIV and AIDS* 2018;13(1):22-27.

29. Frigati L, Archary M, Rabie H, et al. Priorities for decreasing morbidity and mortality in children with advanced HIV disease. *Clinical Infectious Diseases* 2018;66(suppl_2):S147-S51.

30. Fouda GG, De Paris K, Levy O, et al. Immunological mechanisms of inducing HIV immunity in infants. *Vaccine* 2020;38(3):411-15.

31. Mugglin C, Wandeler G, Estill J, et al. Retention in care of HIV-infected children from HIV test to start of antiretroviral therapy: systematic review. *PLoS One* 2013;8(2):e56446. doi: 10.1371/journal.pone.0056446 [published Online First: 2013/02/26]

32. Apondi E, Humphrey JM, Sang E, et al. Trends Over Time for Adolescents Enrolling in HIV Care in Kenya, Tanzania, and Uganda From 2001-2014. *J Acquir Immune Defic Syndr* 2018;79(2):164-72. doi: 10.1097/qai.0000000000001796 [published Online First: 2018/07/10]

https://mc.manuscriptcentral.com/bmjpo

Confidential: For Review Only

33. Bigna JJ, Plottel CS, Koulla-Shiro S. Challenges in initiating antiretroviral therapy for all HIV-infected people regardless of CD4 cell count. *Infect Dis Poverty* 2016;5(1):85. doi: 10.1186/s40249-016-0179-9 [published Online First: 2016/09/07]

Confidential: For Review Only

BMJ Paediatrics Open

Attrition and associated factors among children living with HIV at a tertiary hospital in Eritrea: A retrospective cohort analysis

Journal:	*BMJ Paediatrics Open*
Manuscript ID	bmjpo-2022-001414.R1
Article Type:	Original research
Date Submitted by the Author:	06-May-2022
Complete List of Authors:	Mengistu, Samuel; Nakfa Hospital, General Medicine; Orota School of Medicine and Dentistry, Medicine<br>Ghebremeskel, Ghirmay; Nakfa Hospital, General Medicine<br>Rezene, Aron; Edaga Hamus Hospital, Ministry of health Maekel branch, Asmara, Eritrea<br>Idris, Mahmud ; Department of pediatrics and child health, Orotta College of Medicine and Health Sciences, Asmara, Eritrea<br>Tikue, Tsegereda; Department of pediatrics and child health, Orotta College of Medicine and Health Sciences, Asmara, Eritrea<br>Hamida, Mohammed ; Unit of Medical Microbiology, Orotta College of Medicine and Health Sciences (OCMHS).<br>Achila, Oliver ; Unit of Clinical Laboratory Science, Orotta College of Medicine and Health Sciences (OCMHS).
Keywords:	Virology, HIV, Mortality, Epidemiology

SCHOLARONE™
Manuscripts

I, the Submitting Author has the right to grant and does grant on behalf of all authors of the Work (as defined in the below author licence), an exclusive licence and/or a non-exclusive licence for contributions from authors who are: i) UK Crown employees; ii) where BMJ has agreed a CC-BY licence shall apply, and/or iii) in accordance with the terms applicable for US Federal Government officers or employees acting as part of their official duties; on a worldwide, perpetual, irrevocable, royalty-free basis to BMJ Publishing Group Ltd ("BMJ") its licensees and where the relevant Journal is co-owned by BMJ to the co-owners of the Journal, to publish the Work in this journal and any other BMJ products and to exploit all rights, as set out in our licence.

The Submitting Author accepts and understands that any supply made under these terms is made by BMJ to the Submitting Author unless you are acting as an employee on behalf of your employer or a postgraduate student of an affiliated institution which is paying any applicable article publishing charge ("APC") for Open Access articles. Where the Submitting Author wishes to make the Work available on an Open Access basis (and intends to pay the relevant APC), the terms of reuse of such Open Access shall be governed by a Creative Commons licence – details of these licences and which Creative Commons licence will apply to this Work are set out in our licence referred to above.

Other than as permitted in any relevant BMJ Author's Self Archiving Policies, I confirm this Work has not been accepted for publication elsewhere, is not being considered for publication elsewhere and does not duplicate material already published. I confirm all authors consent to publication of this Work and authorise the granting of this licence.

Confidential: For Review Only

What's Known about the subject?

Attrition and associated factors among children living with HIV at a tertiary hospital in Eritrea: A retrospective cohort analysis

Samuel Tekle Mengistu[1*], Ghirmay Ghebrekidan Ghebremeskel[2], Aron Rezene[3], Mahmud Mohammed Idris[4], Tsegereda Gebrehiwot Tikue[5], Mohammed Elfatih Hamida[6], Oliver Okoth Achila[7]

[1]**Samuel Tekle Mengistu**. Nakfa Hospital, Ministry of health Northern Red Sea branch, Nakfa, Eritrea. Email: teklesam7@gmail.com

[2]**Ghirmay Ghebrekidan Ghebremeskel**. Nakfa Hospital, Ministry of health Northern Red Sea branch, Nakfa, Eritrea. Email: GhirmayGGG@gmail.com

[3]**Aron Rezene**. Edaga Hamus Hospital, Ministry of health Maekel branch, Asmara, Eritrea Email: aronrezene19@gmail.com

[4]**Mahmud Mohammed Idris.** Department of pediatrics and child health, Orotta College of Medicine and Health Sciences, Asmara, Eritrea. Email: mahmudfil@yahoo.com

[5]**Tsegereda Gebrehiwot Tikue.** Department of pediatrics and child health, Orotta College of Medicine and Health Sciences, Asmara, Eritrea. Email: proftsigeghebrehiwet@gmail.com

[6]**Mohammed Elfatih Hamida.** Unit of Medical Microbiology, Orotta College of Medicine and Health Sciences (OCMHS). Email: mohelfatih77@gmail.com

[7]**Oliver Okoth Achila.** Unit of Clinical Laboratory Science, Orotta College of Medicine and Health Sciences (OCMHS). Email: oliverachila@gmail.com

*Corresponding author: **Samuel Tekle Mengistu**, email: teklesam7@gmail.com, Nakfa Hospital, Northern Red Sea Ministry of Health, Asmara, Eritrea.

Word count: 5,285

Confidential: For Review Only

Studies in low- and medium-income countries (LMICs) of SSA and Asia have demonstrated that retention of children living with HIV (CLHIV) in care is a major challenge. Multiple evaluations have suggested that in the context of the rapid scale-up of cART, reducing attrition (Lost to follow-up (LTFU) and mortality) rate is a key programmatic priority.

What the study adds?

- Steep decline in enrolment potentially highlights the fact that effective PMTCT services in prenatal clinics reduced MTCT thereby reducing incident pediatric HIV infection.
- A quarter of the patients weren't retained in care.
- Attrition in the first 2 years after enrollment was largely due to death.
- Attrition was mostly associated with enrollment in recent years; malnutrition; advanced HIV disease (III or IV) at enrollment; initiation of backbones other than AZT+3TC, and residence outside Zoba Maekel.

Confidential: For Review Only

Abstract

Background: Reducing attrition in pediatric HIV positive patients in combined antiretroviral therapy (cART) programs in sub–Saharan Africa (SSA) is a challenge in the region. This study explored the rates and predictors of attrition in children initiated on cART at the principal follow-up facility in Asmara, Eritrea.

Methods: This was a retrospective cohort study using routinely collected programmatic data among all pediatric patients on cART between 2005 and 2020 conducted at Orotta National Referral and Teaching Hospital. Kaplan–Meier estimates of the likelihood of attrition and multivariate Cox proportional hazard models were used to assess factors associated with attrition. All *p*-values were 2-sided and the level of significance was set at $p < 0.05$ for all analyses.

Results: The study included 710 participants (males: 374 (52.7%) vs females: 336 females (47.3%). After 5364 person-years follow-up, attrition occurred in 172 (24.2%) patients. The crude incidence rate of attrition was 3.2 events /100 person-years (PY), mortality rate and lost to follow-up (LTFU) were 2.7/100 PY and 1.2/100 PY, respectively. In the adjusted Cox proportional hazards model, independent predictors of attrition were male gender (AHR=1.6, 95% CI 1–2.4), residence outside Zoba Maekel (AHR=1.5, 95% CI, 1–2.3); Later enrollment years (2010-2015: AHR=3.2, 95% CI, 1.9-5.3) (>2015: AHR=6.1, 95% CI, 3-12.2); WHO BMI for age- Z-score (BAZ) < -2 (AHR = 1.4, 95% CI, 0.9-2.1); advanced HIV disease (WHO III or IV) at enrollment (AHR = 2.2, 95% CI, 1.2-3.9), and Initiation on Zidovudine+Lamuvudine (AZT+3TC) or other cART backbones (UHR = 2, 95% CI, 1.2-3.2). In contrast, a reduced likelihood of attrition was observed in children with a record of cART changes (UHR=0.2, 95% CI, 0.15-0.4).

Conclusion: Low incidence of attrition was recorded in this program. However, the high mortality rate in the first 24 months of treatment and late presentation are worrisome. Therefore, the need for data-driven interventions designed to improve program quality and outcomes are of high priority.

Key words: *Risk Factors, Pediatrics, HIV/AIDS, Attrition, lost to follow-up, Eritrea*

https://mc.manuscriptcentral.com/bmjpo

Introduction

Despite progress in HIV prevention, care, and treatment, HIV in children remains a major public health burden. A recent Joint United Nations Programme on HIV/AIDS (UNAIDS) report suggested that of the 37.7 million [30.2–45.1 million], people living with HIV (PLWH) in 2020; Children < 14 years' account for ~1.8 million [1.3–2.2 million] [1]. In the same period, 180,000 children were newly infected and 110,000 children died of HIV/AIDS. Importantly, a disproportionate number of these children were from sub-Saharan Africa (SSA) where resources are limited and where infectious disease burden is high.

In many ways, the prevailing situation undermines the aspirational UNAIDS 90-90-90 target to end HIV/AIDS epidemic by 2030 (Project 2030) [2]. Although cART enables PLWH to live long and healthy lives and marked improvements have been registered in almost all principal landmarks in the HIV care cascade globally, empirical data from low- and medium-income countries (LMICs) in SSA and Asia suggests that children living with HIV (CLHIV), particularly young adolescents, are still experiencing an upswing in HIV/AIDS-related mortality [3]. Multiple studies in LMIC settings have also demonstrated that a large proportion of CLHIV continue to present to care centers with advanced HIV disease and that retention is a major challenge [3-5]. In this regard, emerging reports from the region point (sensu stricto) at significant problems in the fast-track UNAIDS 90-90-90s diagnose (early) - treat (promptly) – retain (to ensure viral suppression) paradigm, thereby putting targeted timelines off track [2].

Responses towards this problem call, partly to be precise, for rapid scale-up of cART and reduction of attrition (Lost to follow-up (LTFU) and mortality) in HIV/AIDS programs in LMICs. This suggestion emanates from data showing that attrition is high among younger (children < 14 years) – approximately 14% at 12 months follow-up and 22% by 36 months [6 7]. Further, some experts have argued that reducing attrition rates is not only necessary to ensure ongoing receipt of cART; it also allows clinicians to evaluate the emergence of medication toxicity; the opportunity to prevent, manage or treat opportunistic infections (IOs), detect treatment failure; and initiate prompt switching of cART. Additional benefits include ancillary services such as social support and counseling. In this regard, studies on attrition and

Confidential: For Review Only

associated factors among CLHIV in jurisdictions across SSA should be a principal research concern in the foreseeable future.

Therefore, the primary aim of this study was to describe the incidence of attrition and associated factors among CLHIV in Asmara, Eritrea - a country with one of the lowest HIV prevalence in SSA [8]. Data gathered from studies of this kind can be used as a benchmark for gauging programmatic successes and weaknesses [6] and to identify critical gaps and opportunities for context-specific interventions aimed at minimizing attrition and maximizing retention. Secondarily, we explored multiple temporal trends associated with program performance, mortality, and enrolment rates.

Methodology

Study design and setting

A retrospective cohort study was conducted in the pediatric HIV/AIDs follow-up clinic in National Pediatric Referral Hospital (NPRH). HIV/AIDs follow-up clinic in NPRH was commissioned in 2005, making it the first institution in Eritrea to offer cART to CLHIV. Before the decentralization of services to other zones (2010), NPRH (in the Maekel zone) was the only institution offering cART to CLHIV in the country. In total, 822 children under the age of 15 years received service and /or have been enrolled at the clinic since its inception.

Data collection and approach

We used the data collected in the ONRTH pediatric cART and follow-up program electronic database (ART DHIS 2). The retrieved data was robustly reviewed and then cross-referenced with patients' clinical chart registries. Data retrieved from this repository was exported to Microsoft (MS) excel platform. Baseline data collected included: date of birth/age at enrolment, gender, address, and cART initiation date, initial cART regimen, presence of tuberculosis (TB), baseline WHO clinical stage, baseline CD4 count, weight, and hemoglobin (Hg) concentration, and CBC. Height is not available in the electronic medical records, thus being retrieved from clinical chart registries systematically. WHO

https://mc.manuscriptcentral.com/bmjpo

Confidential: For Review Only

standard deviations for weight for age, height for age and Body mass index (BMI) were computed using the WHO Standard deviation for emergency nutritional assessment software (www.nutrisurvey.net/ena/ena.html). Recruitment details are illustrated in Figure 1.

Figure 1: Flow chart for study participant recruitment.

Operational Definitions

- Death event was defined as deaths occurring after enrollment in follow-up, but before December 31st, 2020 [9-11].
- Patients were considered LTFU if they had missed a scheduled visits for ≥ 3 consecutive months [9].
- Attrition was defined as children who were LTFU or died during follow-up in clinic [6 12].
- Retention – children who were alive and on follow-up in the clinic at the end of the study period and children who were transferred out at any time during study period [12].
- Advanced HIV disease was defined as CD4 cell count <200 cells/mm^3 and/or WHO stage 3 or 4 events for patients > 5 years of age [13].
- Late presentation was defined as patients enrolled with advanced WHO HIV stage (III/IV) and/or T-cell CD4+ count less than 200 cells/μL.
- Multiple measures were used to measure nutritional status. These included weight-for-age Z-score (WAZ), height-for-age (HAZ), and BMI-for -age Z-score (BAZ). Under nutrition underscores BAZ < − 2 SD; WAZ ≤−2 z-score, and/or HAZ ≤−2 z-score.
- Residence outside Zoba Maekel – Zoba Maekel is one of the administrative zones in Eritrea. Therefore, residence outside Zoba Maekel refers to children who reside outside this administrative zone.
- Adherence was assessed at each follow-up visit as good, fair, and poor if a child missed <5 %, <10%, and >10% doses respectively of the expected monthly doses.

https://mc.manuscriptcentral.com/bmjpo

Confidential: For Review Only

Data analysis

The retrieved data was exported to IBM SPSS (version 26) and STATA version 12.0 (STATA Corporation, College Station, TX), where it was organized and cleaned for analysis. Descriptive statistics for categorical variables were analyzed using chi-square/Fisher's exact test and summarized using frequency and percentage. Depending on data distribution, quantitative data was summarized using mean (±SD) or median (IQR). Suitable parametric or nonparametric statistics were subsequently employed to evaluate differences. Incidence of attrition was calculated per 100 person years (95% CI). Furthermore, Kaplan-Meier curves describe and compare retention and survival at different intervals of follow-up by gender, address, cohort year, adjusted hazard ratio (AHD), initial cART backbone, and record of cART change. Log rank test was used to evaluate difference in equality of survival functions. All LTFU and transfer-outs were censored on the date of their last visit. Estimates of hazard ratios for attrition, there 95% CI and quantification of associations were evaluated using Cox Proportional Hazards models. Log-log plots and plots of Schoenfeld's residuals were used to evaluate proportional hazards assumption. To adjust for possible confounding, multivariate Cox regression models were ultimately fitted. The final results are presented as AHR with 95% confidence interval (CI). All p-values were 2 tailed and values < 0.05 were regarded as significant.

Ethical consideration

Ethical approval was obtained from the Ministry of Health (MOH) research Ethics and Protocol review committee with a letter of reference: 02/05/21. All data collected from the records of respondents will be kept confidential at all times. Consent to participate was not obtained from the patients or guardians because the study was based on anonymized patient records and this was waived by the ethical committee. All procedures of the study also followed the recommendation of the Declaration of Helsinki Convention.

Patient and public involvement

Confidential: For Review Only

Patients or the public WERE NOT involved in the design, or conduct, or reporting, or dissemination plans of our research.

Results

From September 2005- December 2020, a total of 822 CLHIV were enrolled in ONPRH pediatric cART treatment and follow-up program. After eligibility evaluation, 710 (86.3%) participants were included in our analysis. Stratification of enrollment with respect to specific time frames indicated that 480(67.6%), 180(25.4%), and 50(7.0%) were enrolled into care between 2005-2010; 2011-2015; and 2016-2020, respectively (Table 1). Eligible participants were followed for a total of 5,364 person-years follow-up (PYFU). In this period, 65 (9.2%) died; 107 (15.1%) were LTFU; 153 (21.5%) were transferred out, and 385 (54.2%) are currently on treatment.

Confidential: For Review Only

Table 1. Baseline characteristics of HIV-infected children and adolescents in the ONPRH cART treatment center, Asmara, Eritrea (2005–2020).

Patients' characteristics	Total N (%)	2005-2010	2011-2015	2016-2020	P-value ($\chi 2$)
**Number enrolled**	710	480(67.6)	180(25.4)	50(7.0)	
**Gender**					
Male	374 (52.7)	237 (63.4)	111 (29.7)	26 (7)	**0.01 (7.9)**
Female	336 (47.3)	243 (72.3)	69 (20.5)	24 (7.2)	
**Age on enrollment in years, median (IQR)**	6 (3-9)	5 (3-8)	7 (4-10)	5 (2-11)	**0.001[a]**
<=5 years	274 (38.6)	198 (72.3)	52 (19)	24 (8.7)	**<0.001 (27.5)**
6-10 years	284 (40)	202 (71.1)	72 (25.4)	10 (3.5)	
>10 years	152 (21.4)	80 (52.6)	56 (36.8)	16 (10.6)	
**Address**					
Maekel	516 (72.7)	358 (69.4)	124 (24)	34 (6.6)	0.25 (2.7)
Outside Maekel	194 (27.3)	122 (62.9)	56 (28.9)	16 (8.2)	
**Disease stage**					
Early	191(26.9)	134 (70.2)	46 (24)	11 (5.8)	0.5 (1)
Advanced	519 (73.1)	346 (66.7)	134 (25.8)	39 (7.5)	
**TB- status**					
Symptomatic and under treatment	16 (2.4)	7 (43.8)	6 (37.5)	3 (18.8)	0.5 (5.9)
Not symptomatic	625 (97.6)	443 (70.9)	135 (21.6)	47 (7.5)	
**CD4 count, Median (IQR)**	274 (150-442)	265 (151-392.7)	307 (137-555)	340 (200-888)	**0.012[a]**
**CD4 percentage, Median (IQR)**	12 (7.4-17)	11.1 (7.1-16.5	13.6 (9-21)	14.3 (7.5-21.9)	**0.007[a]**
**Hemoglobin, mean(±SD)**	11.8(±13.5)	12.5 (±17.2)	10.6 (±2.1)	11 (±1.6)	**0.060[a]**
Anemia	264 (45.7)	155 (58.7)	89 (33.7)	20 (7.6)	0.3 (2.2)
No Anemia	313 (54.3)	197 (62.9)	88 (28.2)	28 (8.9)	
**WAZ, median (IQR)**	-2.6 (-3.6- -1.7)	-2.5 (-3.5- -1.7)	-2.8 (-3.8- -1.7)	-2.9 (-3.8-1.6)	0.263[a]
WAZ=>-2	141 (31.9)	98 (69.5)	32 (22.7)	11 (7.8)	0.7 (0.5)
WAZ <-2	302 (68.1)	202 (66.9)	78 (25.8)	22 (7.3)	

**HAZ, median (IQR)**	-2.75 (-3.8 - -1.7)	-2.6 (-3.7 -- -1.5)	-2.9 (-4 - -1.8)	-3(-3.9 - -1.8)	0.063[a]
HAZ=>-2	223 (32.7)	161 (72.2)	49 (22)	13 (5.8)	0.1 (4.3)
HAZ <-2	461 (67.3)	296 (64.2)	129 (28)	36 (7.8)	
**BAZ, median (IQR)**	-1.58 (-2.8--0.69)	-1.5 (-2.9--0.5)	-1.6 (-2.7--1)	-1.7 (-3--0.6)	0.385[a]
BAZ=>-2	378 (60.4)	257 (68)	97 (25.7)	24 (6.3)	
BAZ <-2	247 (29.6)	162 (65.6)	64 (25.9)	21 (8.5)	0.5 (1)
**cART backbone**					
AZT+3TC	508 (71.7)	347 (68.3)	148 (29.1)	13 (2.6)	
Other backbones	201 (28.3)	133 (66.2)	31 (15.4)	37 (18.4)	**<0.001 (62)**
**NNRTI**					
EFV	366 (51.6)	262 (71.6)	90 (24.6)	14 (3.8)	**<0.001 (13)**
NVP	340 (48.4)	216 (63.5)	88 (25.8)	36 (10.6)	
**Current cART regimen**					
First line cART	633 (89.2)	429 (67.8)	157 (24.8)	47 (7.4)	0.3 (1.9)
Second line cART	77 (10.8)	51 (66.2)	23 (29.9)	3 (3.9)	
**Adherence**					
Suboptimal Adherence	91 (12.3)	46 (50.5)	39 (42.9)	6 (6.6)	**<0.001 (17)**
No record	619 (87.2)	434 (70)	141 (22.8)	44 (7.2)	
**cART change**					
cART changed at least once	516 (75)	**340 (65.9)**	148 (28.7)	28 (5.4)	**<0.001 (1.2)**
No cART change	172 (25)	120 (69.8)	30 (17.4)	22 (12.8)	
Duration of follow up in years, Median (IQR)	8 (4-11)	10 (7-12)	6 (4-8)	2 (1-3.25)	**<0.001[a]**

Abbreviations: cART; combined antiretroviral therapy, NNRTI; non-nucleoside reverse transcriptase inhibitor, WAZ; weight for age standard deviations, HAZ; height for age standard deviations, and BAZ; Body mass index for age standard deviations

Definitions: **Other back bones** refer to Tenofovir disoproxil fumarate (TDF) + Emtricitabine (FTC), Abacavir (ABC) + 3TC or Stavudine (D4T) + 3TC. **Anemia** was defined as hemoglobin (Hg) level is < 11 g/dl for children <5 years old, <11.5 g/dl for children 5–11.9 years old, and <12 g/dl for children >12 years old.

Superscript: a; Mann-Whitney u test.

Comparisons of proportions were performed by using the $\chi 2$ test, medians by using Mann-Whitney tests (a).

Sociodemographic characteristics of CLHIV

The median (IQR) age at enrollment to the clinic was 6 years (IQR: 3 -9 years). The median (IQR) of age from 2005 – 2010; >2010-2015, > 2016-2020 were 5 years (IQR: 3-8 years); 7 years (IQR: 4-10 years); and 5 years (IQR: 6.25- 12.75 ears), respectively. In terms of sex, the number of males and females was relatively similar (374 (52.7%) vs. 336 (47.3%)). Majority of participants were from Maekel Zone (72.7%).

Clinical and disease-related characteristics of CLHIV at baseline

Assessment of clinic-level factors indicated that the median CD4 count and percentage at treatment initiation were 274 cells/μl (IQR, 150-442 cells/μl) and 12% (7.4-17%), respectively. In the relevant time frames, the median (IQR) values were 265 cells/μl (151-392.7) cells/μl in 2005 – 2010; 307 cells/μl (137-555) cells/μl in >2010-2015; and 340 cells/μl (200-888) cells/μl in 2016-2020, (p value = 0.012).

Confidential: For Review Only

Similarly, the median (IQR) for CD4 percentages in 2005-2010; >2010-2015; and 2016-2020 were 11.1% (7.1-16.5%); 13.6% (9-21%) and 14.3% (7.5-21.9%), respectively (p-value = 0.007). Overall, and in the following order, 519(73.1%); 264 (45.7%); 16(2.4%) had advanced (STAGE III or IV) disease, anemia and TB before initiation of cART, respectively. The proportion of children with WAZ z-score <-2 SD (underweight), HAZ z-score <-2 SD (stunted), and BAZ z-score <-2 SD (undernourished) were 302 (68.1%);461 (67.3%) and 247 (29.6%), respectively. Notably, and despite the difference in enrollment in the successive time frames, no improvements were observed for these growth parameters (anemia, WAZ, HAZ, and BAZ) (Table 1).

Treatment-related characteristics and outcomes

The preferred cART backbone was AZT+3TC (71.7%). Suboptimal adherence was recorded in 12.3% of patients while the majority (72%) of participants had a history of cART change at least once.

Prevalence of retention and attrition

The overall attrition (death + LTFU), deaths, and LTFU are presented in figure 2. According to this analysis, the overall death rate and LTFU in person years were 2.7 [95% CI 2.1-3.5] /100 PY and 1.2 [95% CI 1-1.5] /100 PY. Attrition at (0 to ≤6 months, >6 to ≤12 months, >12 to ≤24 months, >24 to ≤36 months, >36 to ≤48 months, and >48 to ≤60 months of follow-up were 3.6%, 2%, 2.9%, 1.3%, 1.4% and 1.2%, respectively.

Figure 2. Frequency of attrition, deaths, and LTFU at specified time points within the first 60 months after initiation of cART

Factors Associated with retention and attrition

Out of 710 CLHIV included in the study, attrition occurred in 172 (24.2% [95% CI 20-26.3]) patients. The crude incidence and overall survival duration were 3.2 events per 100- person-years (PY) (95% CI

Confidential: For Review Only

2.7-3.7) and 14 years (95% CI, 13.4-14.8). Duration of follow-up in years for attrition and retention were 4.5 years (IQR: 1-9 years) and 9 years (IQR: 6-11 years), respectively. In terms of proportions, attrition rates were higher in males (104(27.8%) vs 68(20.2%) in females; residence outside Maekel (57(29.4%) vs 115(22.3%); advanced HIV (WHO stage III and IV) 149(28.7) vs 23(12%) for early presentation; presence of anemia (72 (27.5%) vs 54 (17.3%) in no anemia; WAZ z-score <-2 (underweight) 83(27.5%) vs 27(19.1%) in WAZ z-score >-2; HAZ z-score <-2 (stunting) 122 (26.5%) vs 42(18.8%) in HAZ z-score > -2; BAZ score <-2 (Under nutrition) (73(29.6%) vs 76(20.1%) in BAZ z-score >-2; AZT+3TC, Else cART backbone 71(35.3%) vs 101(19.9%) in AZT + 3TC; No record of cART change or switching(77(44.8%) vs 92(17.8%). (Table 2)

Table 2. Characteristics of the study participants stratified by survival outcome in the ONPRH cART treatment center, Asmara, Eritrea (2005–2020).

Cohort Characteristics	Total n (%)	Attrition n (%)	Retention n (%)	P-value ($\chi 2$)
**Gender**				
Male	374 (52.7)	104 (27.8)	270 (72.2)	**0.01 (5.5)**
Female	336 (47.3)	68 (20.2)	268 (79.8)	
**Age on enrollment in years, median (IQR)**	6 (3-9)	6 (3-10)	6 (3-9)	0.38[a]
<=5 years	274 (38.6)	62 (22.6)	212 (77.4)	0.09 (4.7)
6-10 years	284 (40)	63 (22.2)	221 (77.8)	
>10 years	152 (21.4)	47 (30.9)	105 (69.1)	
**Enrollment Year, Median (IQR)**				0.24[a]
2005-2010	265 (37.3)	111 (23.1)	369 (76.9)	0.23 (2.9)
2011-2015	289 (40.7)	44 (24.4)	136 (75.6)	
2016-2020	156 (22)	17 (34)	33 (66)	
**Address**				
Maekel	516 (72.7)	115 (22.3)	401 (77.7)	**0.049 (3.8)**
Outside Maekel	194 (27.3)	57 (29.4)	137 (70.6)	
**Disease stage**				
Early	191(26.9)	23 (12)	168 (88)	**<0.001 (21)**
Advanced	519 (73.1)	149 (28.7)	370 (71.3)	
**TB- status**				
Symptomatic and under treatment	16 (2.4)	5 (31.2)	11 (68.8)	0.5 (0.4)
Not symptomatic	625 (97.6)	151 (24.2)	474 (75.8)	
**CD4 count, Median (IQR)**	274 (150-442)	216 (100-371)	287 (163-452)	**<0.001[a]**
**CD4 percentage, Median (IQR)**	12 (7.4-17)	9.9 (6-14.5)	12.5 (8.2-19)	**<0.001[a]**
**Hemoglobin, Mean (±SD)**	11.8(±13.5)	10.7 (9.2-11.8)	11.2 (9.8-12.3)	**<0.001[b]**
Anemia	264 (45.7)	72 (27.5)	192 (72.5)	**0.004 (8.4)**
No Anemia	313 (54.3)	54 (17.3)	259 (82.7)	
**WAZ, median (IQR)**	-2.6 (-3.6--1.7)	-3 (-4.3- -2)	-2.5 (-3.5- --1.6)	**0.001[a]**
WAZ=>-2	141 (31.9)	27 (19.1)	114 (80.9)	**0.05 (3.5)**
WAZ <-2	302 (68.1)	83 (27.5)	219 (72.5)	
**HAZ, median (IQR)**	-2.75 (-3.8--1.7)	-3.2 (-4- -1.9)	-2.5 (3.7- --1.5)	**0.001[a]**
HAZ=>-2	223 (32.7)	42 (18.8)	181 (81.2)	**0.028 (4.8)**
HAZ <-2	461 (67.3)	122 (26.5)	339 (73.5)	

Confidential: For Review Only

**BAZ, median (IQR)**	-1.58 (-2.8--0.69)	-1.9 (-3.6- -0.7)	-1.5 (-2.6- -0.6)	**0.012[a]**
BAZ=>-2	378 (60.4)	76 (20.1)	302 (79.9)	**0.007 (7.3)**
BAZ <-2	247 (29.6)	73 (29.6)	174 (70.4)	
**cART backbone**				
AZT+3TC	508 (71.7)	101 (19.9)	407 (80.1)	**<0.001 (18.6)**
Other backbones	201 (28.3)	71 (35.3)	130 (64.7)	
**NNRTI**				
EFV	366 (51.6)	91 (24.9)	275 (75.1)	0.67 (0.1)
NVP	340 (48.4)	80 (23.5)	260 (76.5)	
**Current cART regimen**				
First line cART	633 (89.2)	156 (24.6)	477 (75.4)	0.45 (0.5)
Second line cART	77 (10.8)	16 (20.8)	61 (79.2)	
**Adherence**				
Suboptimal Adherence	91 (12.3)	24 (26.4)	67 (73.6)	0.6 (0.2)
No record	619 (87.2)	148 (23.9)	471 (76.1)	
**cART change**	516 (75)	92 (17.8)	424 (82.2)	**<0.001 (50.5)**
No cART change	172 (25)	77 (44.8)	95 (55.2)	
Duration of follow up in years, Median (IQR)	8 (4-11)	4.5 (1-9)	9 (6-11)	**<0.001[a]**

Abbreviations: cART; combined antiretroviral therapy, NNRTI; non-nucleoside reverse transcriptase inhibitor, WAZ; weight for age standard deviations, HAZ; height for age standard deviations, and BAZ; Body mass index for age standard deviations
Definitions: **Other back bones** refer to Tenofovir disoproxil fumarate (TDF) + Emtricitabine (FTC), Abacavir (ABC) + 3TC or Stavudine (D4T) + 3TC. **Anemia** was defined as hemoglobin (Hg) level is < 11 g/dl for children <5 years old, <11.5 g/dl for children 5–11.9 years old, and <12 g/dl for children >12 years old.
Superscript: a; Mann-Whitney u test. b; Independent sample t-test
Comparisons of proportions were performed by using the $\chi2$ test, medians by using Mann-Whitney tests (a) and means using independent sample t-test.

Kaplan-Meier analysis for Attrition incidence

Kaplan-Meier survival curves with log rank tests were constructed to compare the cumulative incidence of attrition by gender, address, cohort year, AHD, initial cART backbone, and record of cART change (See Figure 3 and Table 3). Overall, being male was associated with shorter survival (13 (95% CI, 2-14 years) vs 13.8 (95% CI, 13–14 years)) (log rank test, p = 0.008) (figure 2a). Significantly shorter mean survival duration was observed with increasing age at baseline: <=5 years: 15.1 (14.2-16) years' vs 6-10 years: 11.7(11.1-12.2 years) and >10 years: 8.4(7.43-12.2 years) years (log rank test p < 0.001). Children who reside outside Maekel were also found to have a significantly shorter duration of survival in the clinic mean (11 years (95% CI, 10-11.7 years)) compared to those from Maekel (14.5 years (95% CI, 13.8-15.4 years)) (log rank test p = 0.002 (Figure 2b). Late enrollment calendar years were also associated with shorter mean survival duration (2005-2009: 14.8 years (95% CI, 14–15.5 years) vs 2010-2014: 7.5 years (95% CI, 7-8 years) vs 2015-2020: 3.1 years (95% CI, 2.7-3.5 years) (log rank test p = 0.008 (figure 2c). Children with AHD at presentation had a significantly shorter mean survival time in

Confidential: For Review Only

ONPRH (13 years (95% CI 12.3-14)) compared to 15 years (95% CI, 14.2-15.7 years) for those without. The survival time for AZT+3TC, else group 11.3 years (95%CI, 9.8–12.8 years) was significantly shorter compared to the AZT+3TC group 14 years (95%CI, 13.4–14.5 years) (log rank test $p < 0.001$). (Figure 2d). Moreover; children with a record of cART change had significantly longer mean survival time in the clinic 13 years (95% CI 13-14 years) as compared (7.5 years (95% CI, 6.6-8.4 years) (log rank test $p < 0.001$). Others associated with shorter survival were: anemia at ART initiation (Hemoglobin < 8 g/dl) ($p = 0.032$), WAZ score <-2 12 years (11.4-12.8 years); HAZ score <-2 (12.7 years (12-13.4 years); BAZ score <-2 (12 years (11-13 years).

Figure 3. Kaplan-Meier cumulative incidence of attrition unadjusted curves for children followed in ONPRH from 2005- 2020

Confidential: For Review Only

Table 3. Crude incidence, rate of attrition, and Kaplan-Meier survival estimates of children followed in the ONPRH cART treatment center, Asmara, Eritrea (2005–2020).

Cohort Characteristics	Incidence of attrition per 100 person years (95% CI)	Mean survival duration in years, 95% CI	P-value (log-rank)
**Total**	3.2 (2.7-3.7)	14.1 (13.4-14.8)	
**Gender**			
Male	3.8 (31.-4.6)	13.2 (2.1-14.3)	**0.008 (6.9)**
Female	2.5 (2-3.2)	13.8 (13.1-14.4)	
**Age in years at enrollment**			
<=5 years	2.5 (1.9-3.2)	15.1 (14.2-16)	**<0.001 (36.8)**
6-10 years	2.8 (2.2-3.6)	11.7 (11.1-12.2)	
>10 years	6.7 (5-8.9)	8.4 (7.43-9)	
**Address**			
Maekel	2.7 (2.3-3.3)	14.6 (13.8-15.4)	**0.002 (9.8)**
Outside Maekel	4.6 (3.5-5.9)	10.9 (10-11.7)	
**Disease Stage at presentation**			
Early	1.4 (0.9-2.1)	15 (14.2-15.7)	**<0.001 (22)**
Advanced	3.9 (3.3-4.6)	13.2 (12.3-14)	
**TB-Status**			
Symptomatic and under treatment	3.1 (2.6-3.7)	9.9 (6.9-12.8)	0.2 (1.5)
Not symptomatic	6.5 (2.7-15.8)	14 (13.3-14.8)	
**Hemoglobin**			
Anemia	2.2 (1.7-2.9)	11.5 (11-12)	**0.004 (8.3)**
Normal	3.7 (2.9-4.7)	12.8 (12-13)	
**WAZ**			
WAZ=>-2	2.1 (1.48-3.1)	13 (12.4-13.7)	**0.014 (6)**
WAZ <-2	3.5 (2.8-4.4)	12 (11.4-12.8)	
**HAZ**			
HAZ=>-2	2.3 (1.7-3.2)	14.9 (13.7-16)	**0.019 (5.4)**
HAZ <-2	3.5 (3-4.2)	12.7 (12-13.4)	
**BAZ**			
BAZ=>-2	2.4 (1.9-3)	15 (14-15.9)	**<0.001 (12.4)**
BAZ <-2	4.4 (3.5-5.5)	12 (11-13)	
**Initial cART backbone**			
AZT+3TC	2.3 (1.9-2.8)	13.9 (13.4-14.5)	**<0.001 (54.5)**
Other backbones	7.1 (5.6-9)	11.3 (9.8-12.8)	
**NNRTI**			
EFV	3.2 (2.6-3.9)	13 (12.3-13.7)	0.9 (0.015)
NVP	3 (2.5-3.9)	14.2 (13.3-15.2)	
**Current cART regimen**			
First line cART	3.34 (2.8-3.9)	12.9 (12.3-13.4)	0.15 (2)
Second line cART	2.2 (1.3-3.7)	15.7 (14.3-17)	
**Adherence**			
Suboptimal Adherence	2.9 (1.9-4.3)	12.6 (11.8-13.4)	0.68 (0.16)
No record	3.2 (2.7-3.7)	14.3 (13.6-15)	
**cART Change**			
cART change	1 (0.84-1.3)	13.6 (13-14)	**<0.001 (136)**
No cART change	2 (1.7-2.5)	7.5 (6.6-8.4)	

Abbreviations: cART; combined antiretroviral therapy, NNRTI; non-nucleoside reverse transcriptase inhibitor, WAZ; weight for age standard deviations, HAZ; height for age standard deviations, and BAZ; Body mass index for age standard deviations

Definitions: **Other back bones** refer to Tenofovir disoproxil fumarate (TDF) + Emtricitabine (FTC), Abacavir (ABC) + 3TC or Stavudine (D4T) + 3TC. **Anemia** was defined as hemoglobin (Hg) level is < 11 g/dl for children <5 years old, <11.5 g/dl for children 5–11.9 years old, and <12 g/dl for children >12 years old.

Comparisons of survival duration were performed by using the Log-rank test and its p-value.

Independent predictors of attrition rate in children enrolled in ONPRH cART treatment and follow-up clinic

The hazard ratios (unadjusted and adjusted) for attrition in 701 patients and associated p-values for difference in attrition across specific cohort characteristics are given in Table 4. In the univariate Cox proportional hazards model, increased risk of attrition was associated with being male (UHR=1.4, 95% CI 1–2, p=0.01); Residence outside Zoba Maekel (UHR=1.6, 95% CI 1.2-2.2, p=0.002); WHO BAZ score <-2 (UHR=1.8, 95% CI 1.3-2.5, p=0.001); WHO HAZ score (UHR=1.5, 95% CI 1-2.1, p=0.02); presence of anemia (UHR=1.7, 95% CI 1.7 1.2-2.4, p=0.005); advanced disease (WHO III or IV) at presentation (UHR=2.9, 95% CI 1.8-4.3, p<0.001); Initiation on AZT+3TC or other cART backbones (UHR=3, 95% CI 2.2-4.2, p<0.001). In contrast, reduced risk of attrition was associated with cART changes (UHR=0.16, 95% CI 0.12-0.2, p<0.001).

In the adjusted Cox proportional hazards model, independent predictors of attrition were male gender (AHR=1.6, 95% CI 1–2.4, p=0.01), Residence outside Zoba Maekel (AHR=1.5, 95% CI 1–2.3, p=0.02); Later enrollment years (2010-2015: AHR=3.2, 95% CI 1.9-5.3, p<0.001) (>2015: UHR=6.1, 95% CI 3-12.2, p<0.001); BAZ < -2 (AHR= 1.4, 95% CI 0.9-2.1, p=0.05); advanced HIV disease (WHO III or IV) at enrollment (AHR= 2.2, 95% CI 1.2-3.9, p=0.004); Initiation of AZT+3TC or other cART backbones (UHR=2, 95% CI 1.2-3.2, p=0.005). In contrast, a reduced likelihood of attrition was observed in children with a record of cART changes (UHR=0.2, 95% CI 0.15-0.4, p<0.001). Table 4.

https://mc.manuscriptcentral.com/bmjpo

Confidential: For Review Only

Table 4, Cox proportional hazards of attrition among CLHIV and adolescents at ONPRH cART treatment center, Asmara, Eritrea (2005–2020)

Cohort Characteristics	Unadjusted HR (95%CI)	p-value	Adjusted HR (95%CI)	p-value
**Gender**				
Female	1 (*Ref.*)	**0.01**	1 (*Ref.*)	**0.01**
Male	1.4 (1-2)		1.6 (1-2.4)	
**Age at enrollment**				
<= 5	1 (*Ref.*)		1 (*Ref.*)	
6-10	1.3 (0.9-1.8)		1.1 (0.7-1.6)	
>10	3.3 (2.2-5)		1.5 (0.9-2.5)	
**Address**				
Maekel	1 (*Ref.*)	**0.002**	1 (*Ref.*)	**0.02**
Outside Maekel	1.6 (1.2-2.2)		1.5 (1-2.3)	
**Year of Enrollment**				
< 2010	1 (*Ref.*)		1 (*Ref.*)	
2010-2015	1.9 (1.3-2.7)		3.2 (1.9-5.3)	**<0.001**
>2015	4.2 (2.5-7.2)		6.1 (3-12.2)	**<0.001**
**BMI for age, z-score**				
**>= 2**	1 (*Ref.*)	**0.001**	1 (*Ref.*)	**0.03**
**< -2**	1.8 (1.3-2.5)		1.4 (0.9-2.1)	
**Height for age, z-score**				
**>= 2**	1 (*Ref.*)	**0.02**		
**< -2**	1.5 (1-2.1)			
**Hemoglobin**				
Normal	1 (*Ref.*)	**0.005**		
Anemia	1.7 (1.2-2.4)			
**Advanced disease at presentation**				
No	1 (*Ref.*)	**<0.001**	1 (*Ref.*)	**0.004**
Yes	2.9 (1.8-4.3)		2.2 (1.2-3.9)	
**TB status**				
Not symptomatic	1 (*Ref.*)	0.23		
Took Anti-TB	1.8 (0.8-5)			
**cART Backbone**				
AZT+3TC	1 (*Ref.*)	**<0.001**	1 (*Ref.*)	**0.005**
Other backbones	3 (2.2-4.2)		2 (1.2-3.2)	
**NNRTI**				
EFV	1 (*Ref.*)	0.9		
NVP	0.95 (0.7-1.2)			
**cART changes**				
No	1 (*Ref.*)	**<0.001**	1 (*Ref.*)	**<0.001**
Yes	0.16 (0.12-0.2)		0.2 (0.15-0.4)	
**Current cART regimen**				
**First line cART**	1 (*Ref.*)	0.16		
**Second line cART**	0.68 (0.4-1.1)			
**Suboptimal adherence**				
No	1 (*Ref.*)	0.2		
Yes	1.1 (0.7-1.7)			

Abbreviations: cART; combined antiretroviral therapy, NNRTI; non-nucleoside reverse transcriptase inhibitor, WAZ; weight for age standard deviations, HAZ; height for age standard deviations, and BAZ; Body mass index for age standard deviations
Definitions: **Other back bones** refer to Tenofovir disoproxil fumarate (TDF) + Emtricitabine (FTC), Abacavir (ABC) + 3TC or Stavudine (D4T) + 3TC. **Anemia** was defined as hemoglobin (Hg) level is < 11 g/dl for children <5 years old, <11.5 g/dl for children 5–11.9 years old, and <12 g/dl for children >12 years old.

Confidential: For Review Only

Discussion

One of the most striking results uncovered in this study was the steep decline in enrollment – 2005-2010 (480(67.6%); 2011-2015 (180(25.4%); 2016-2020 (50(7.0%). Multiple interpretations can be invoked to explain the observed results. First, the utilization of PMTCT services has progressively improved in recent years [8]. In addition, linkage to care (awareness and uptake of PMTCT services and strict adherence to a "Test and Start" treatment paradigm or Option B+[14]) is prompt. Therefore, it's our belief that the number of unrolled infants is marginal. Actually, the low enrollment numbers in 2016 to 2020 may simply be a reflection of better organization and/or delivery of services in PMTCT programs in Zoba Maekel and possible near elimination of mother-to-child transmission (MTCT). An alternative explanation of the observed outcome may be connected to the dramatic reduction in HIV prevalence in the general population - ~ 2.4% in 2003 to 0.36% (~14 000) at the end of 2019 (Antenatal Care 2019 Preliminary Report). Whatever the case, we can cautiously conclude that the data highlights the potential success of HIV programs in Asmara, Eritrea.

Positives aside, our data highlighted specific concerns: attrition in the first 24 months following cART initiation was largely due to premature death; late presentation is frequent; the median age at cART initiation has increased; and attrition rate was constant in the 2005-2010; 2011-2015; 2016-2020 period. The fact that the lower threshold for child and infant cART eligibility in 2011, guidelines have failed to lower the median age at initiation was an unexpected [15 16]. A plausible explanation of this phenomenon relates to the possibility that a reduction in MTCT has substantially reduced the number of infants who are eligible for cART. At the same time, changes in treatment guidelines, and other factors (references from treatment centers); has encouraged the enrollment of older children into care.

https://mc.manuscriptcentral.com/bmjpo

These explanations, while plausible; are entirely speculative. Therefore, a solution to this conundrum requires data on multiple domains in the HIV care cascade – children/infants diagnosed with HIV in PMCTs, hospitals/clinics, or VCTs across Asmara within the study period; the proportion linked to HIV care and initiated on cART; and the proportion retained in HIV care programs. Data on the proportion of patients who were eligible for cART (particularly adolescents living with HIV/AIDS (ALWH) based on previous guidelines; but were not placed on treatment is equally important. Unfortunately, this information is hard to access owing to a variety of complex reasons - lack of linked data across HIV points of service in Eritrea; poor patient tracking; and possible inconsistencies in appointment schedules. Therefore, at the very least, our findings provide further support for the need for linked databases across HIV/AIDS care points within Eritrea. The importance of enhanced outreach and intensive case-finding for LTFU; as standard components of HIV care programs in Eritrea is equally evident.

Further, attrition rate among those receiving care were lower when compared to similar studies from SSA[6]. However, our finding of high attrition and mortality rates in the first 6 months of treatment has been reported by multiple investigators [6 17 19]. In the multicounty treated Asia Pediatric HIV Observational Database; mortality decreased from 10.2 (95% CI: 7.5-13.7) deaths/100 PY in the first 3 months to 4.2 (95% CI, 2.6-6.8) deaths/100 PY) in the 4th and 6th months [18]. The high mortality in the first 6 months of enrollment in care has largely been attributed to late presentation – WHO stage III or IV; chronic diarrhea and weight for height z-score, <-2 (severe wasting), among others. Observational studies suggest that the first 6 months after treatment initiation is the most vulnerable period for HIV positive children in SSA [6]. Studies repeatedly cite the need for context-specific mitigation (Short messaging services (SMS)-based messaging/reminder; improved linkage of child and maternal HIV/AIDS services; intensive case management, among others)[6] directed at maximization of retention at this stage.

Confidential: For Review Only

Furthermore, analysis of incidence of attrition demonstrated a rate of 3.2 (95% CI 2.7-3.7)/100 PY. This incidence estimate is lower when compared to studies conducted in Ethiopia (8.36[95% CI: 7.12−9.80]/100 PY) [18]; Zimbabwe (11.8, [95% CI:11.0–12.7]/100 PY) [19]; Nigeria (10.8/100 PY) [20] and Kenya (23.1/100 PY) [21]. In contrast, the incidence of attrition was higher than what was reported elsewhere [22 23 24]. The variation in incidence between countries in SSA is generally attributed to variations in access and quality of healthcare services; health seeking behaviors between populations [25]; heterogeneity in methodology and definition of outcomes, among others. Finally, we have to highlight the possibility that the LTFUs registered in our study may actually be attributable in large part to undocumented mortality and not undocumented transfers or disengagement from care. Therefore, the possibility that our study underestimated mortality should not be overlooked.

In many respects, the predictors of attrition identified in this study are largely similar to those uncovered in other studies in the region. For instance, evidence from multiple studies in the region have indicated that WHO BAZ<-2 (under-nutrition) and BMI for age z-score <-2 are baseline hazards for cART attrition[9 11 19]. Although the presence of anemia at baseline was associated with an increased hazard of attrition (1.7: 1.2-2.4), the association was attenuated in the adjusted multivariable model. This suggests the presence of confounders. Nevertheless, the conjunction between these parameters should not be ignored. For instance, data suggest that the co-presence of severe malnutrition (SAM)+HIV is associated with increased morbidity and mortality in affected infants [26 27]. Others have argued that HIV+SAM represents a distinct disease stratum/ phenotype with worse prognosis and impaired immune recovery [28]. Importantly, the clinical management of this class of patients is extremely challenging [13]. Highlighting this point, some investigators have suggested that rapid initiation (within 1 week) of cART in children with SAM or WHO stage III or IV disease may be counterproductive and that treatment in these patients should be delayed until

https://mc.manuscriptcentral.com/bmjpo

the patient is stabilized [27]. Based on these considerations, the observed connection between advanced HIV disease (III or IV) at enrollment and attrition rate (aHR=2.2, 95%CI: 1.2-3.9) was predictable. [17]. Indeed, reports from Zimbabwe [19], Nigeria [20], and Myanmar [16] have demonstrated that late presentation remains a challenge in the region.

Additional factors, unique to this setting, which were associated with attrition, included male gender, residence outside Asmara, enrollment in later years and late presentation. In general, data on the relationship between sex and attrition is mixed, with most studies reporting a null relationship while others skew towards either sex [9 29 30]. We believe that these inconsistencies and paradoxical results in cohorts across SSA mirrors the disparate context-specific influences at play in the region. On the other hand, the observed relationship between attrition and residence outside Asmara has bigger implication for practice. First, experts have noted that HIV-related discrimination and internalized shame may constrain access to healthcare in ALWH [31]. For context, ridicule from friends/peers in particular; may force guardians/parents to enroll patients in distant facilities – residence outside Zoba Maekel. This practice can compromise linkage and retention in care by imposing unnecessary out-of-pocket costs (transport) [32-34]. Fear of victimization by others can also prompt delays or failure by parents/guardians to disclose perinatally acquired HIV status [35] – a phenomenon which can promote late presentation. In many ways, the highlighted connection between stigma, attrition, enrollment in later years and late presentation is a common finding in attrition studies on ALWH [13 19 22]. All told, we have to concede that HIV programs in Eritrea are ill-equipped to deal with the complex needs of ALWH. Multiple studies have demonstrated that ALHIV recruitment and retention in care is undermined by lack of psychosocial support, lack of health personnel trained on ALHIV–related issues, barriers to care uptake, and limited support during transition to adult care [36]. By and large, implementation of comprehensive interventions targeted at these barriers will require robust research.

https://mc.manuscriptcentral.com/bmjpo

Confidential: For Review Only

Lastly, in the multivariable proportional hazard's regression, the adjusted hazards of attrition were highest in children without a record of cART changes or on other cART backbones - ABC/3TC; d4T+3TC or TDF+FTC. A casual analysis of these associations will reveal some subtle connections. Note that retention in care may correlate with a higher likelihood of cART changes/switch to other backbones. In contrast, late presentation is associated with higher likelihood of mortality/attrition within the first 6 months of enrollment [37]. This can in turn undercut the prospects of cART switches particularly in settings with a limited range of cART option. If valid, then the latter explanation highlights a major systematic weakness in cART clinics across Eritrea – the limited diversity of cART options for children CLHIV and ALHIV.

Strength and limitations of the study

A major strength of this study is the use of routinely collected information from the largest pediatric cART clinic in Eritrea. Therefore, the results can provide a useful gauge of real-world program successes and weaknesses. Regardless, our evaluation highlighted a number of limitations. First, retrospective designs suffer from multiple constrains – missing data, among others. Missing or unstructured collection of information ($CD4^+$ cell counts or viral load data) and lack of data on causes of death and outcomes in children characterized as LTFU are particular examples. In the absence of tracing information, mortality estimate in this cohort are unclear.

Conclusion

One of the most striking results uncovered in this study was the steep decline in enrollment. This, outcome highlights the possible impact of effective PMTCT services in the country. Further, a Cox proportional hazards modeling suggested that the relatively low attrition rates reported in this study was associated with sex, residence outside Zoba Maekel, later enrollment years, WHO BAZ<-2, advanced disease at enrollment, and nature of cART backbone. In addition, multiple concerns were

https://mc.manuscriptcentral.com/bmjpo
Confidential: For Review Only

highlighted – late presentation; high mortality in the first 24 months after initiation of cART; and a constant attrition rate. From a programmatic point of view, our findings provide further support for the need for linked databases across the HIV/AIDS care cascades within Eritrea. The importance of enhanced outreach and intensive case-finding/tracing for LTFU; as standard components of HIV/AIDS care programs in Eritrea is equally evident. Finally, putting comprehensive clinical and programmatic interventions in place will require good data. Unfortunately, large data gaps were evident in this program. Therefore, research should be prioritized.

Declaration

Ethical approval and consent to participate

Ethical approval for this study was obtained from the Eritrean Ministry of Health (MOH) Research Ethical Committee. Information on the maintenance of data confidentiality and integrity was also provided. As the study is based on patients' clinical card records, consent for data access was waived by the ethical committee in place of the patients. This study conforms to the principles outlined in the Declaration of Helsinki.

Consent for publication

Consent for publication is also waived by the ethical committee in place of patients as the data is secondary.

Confidential: For Review Only

Availability of data and materials

The datasets supporting the conclusions of this article are available from the corresponding author on reasonable request.

Conflict of Interest

The authors have no conflict of interest to declare in this study.

Financial support

The study hasn't received any funding.

Authors' contributions

Conceptualization: Samuel Tekle Mengistu

Data curation: Samuel Tekle Mengistu, and Ghirmay Ghebrekidan,

Formal analysis: Samuel Tekle Mengistu and Oliver Okoth Achila

Investigation: Samuel Tekle Mengistu, Mohammed Elfatih Hamida and Oliver Okoth Achila.

Methodology: Samuel Tekle Mengistu, Ghirmay Ghebrekidan, and Aron Rezene

Project administration: Samuel Tekle Mengistu, Mahmud Mohammed Idris, Tsegereda Gebrehiwot Tikue, Mohammed Elfatih Hamida, and Oliver Okoth Achila

Writing – original draft: Samuel Tekle Mengistu, and Oliver Achila.

Writing – review& editing: Samuel Tekle Mengistu, Ghirmay Ghebrekidan Ghebremeskel, Oliver Achila, Mahmud Mohammed Idris, Tsegereda Gebrehiwot Tikue, Mohammed Elfatih Hamida, and Oliver Okoth Achila.

Confidential: For Review Only

Acknowledgment

We are grateful to National Communicable Disease Control Division, Eritrean Ministry of Health, and ART Health Management Information System (HMIS) developers and technicians. Our sincere appreciation goes to Dr. Araia Berhane, Dr. Fanus Yemane, Dr. Simon Tesfay, Dr. Yonathan Tesfalidet, and Mr. Thomas Asfaha.

Confidential: For Review Only

Reference

1. Global H. AIDS statistics–2019 fact sheet. UNAIDS. *URL: http://www unaids org/sites/default/files/media_asset/UNAIDS_FactSheet_ru pdf* 2020

2. Levi J, Raymond A, Pozniak A, et al. Can the UNAIDS 90-90-90 target be achieved? A systematic analysis of national HIV treatment cascades. *BMJ global health* 2016;1(2):e000010.

3. Bain LE, Nkoke C, Noubiap JJN. UNAIDS 90–90–90 targets to end the AIDS epidemic by 2020 are not realistic: comment on "Can the UNAIDS 90–90–90 target be achieved? A systematic analysis of national HIV treatment cascades". *BMJ global health* 2017;2(2):e000227.

4. Ng'eno B, Mwangi A. Burden of HIV infection among children aged 18 months to 14 years in Kenya: results from a nationally representative population-based cross-sectional survey. *Journal of acquired immune deficiency syndromes (1999)* 2014;66(Suppl 1):S82.

5. Desmonde S, Tanser F, Vreeman R, et al. Access to antiretroviral therapy in HIV-infected children aged 0–19 years in the International Epidemiology Databases to Evaluate AIDS (IeDEA) Global Cohort Consortium, 2004–2015: a prospective cohort study. *PLoS medicine* 2018;15(5):e1002565.

6. Carlucci JG, Liu Y, Clouse K, et al. Attrition of HIV-positive children from HIV services in low-and middle-income countries: a systematic review and meta-analysis. *AIDS (London, England)* 2019;33(15):2375.

7. Phelps BR, Ahmed S, Amzel A, et al. Linkage, initiation and retention of children in the antiretroviral therapy cascade: an overview. *AIDS (London, England)* 2013;27(0 2):S207.

8. Division CDC. ERITREAN ANTIRETROVIRALTHERAPY GUIDELINES. Asmara, Eritrea: Ministry of Health, 2017.

Confidential: For Review Only

9. Zanoni BC, Phungula T, Zanoni HM, et al. Risk factors associated with increased mortality among HIV infected children initiating antiretroviral therapy (ART) in South Africa. *PLoS One* 2011;6(7):e22706. doi: 10.1371/journal.pone.0022706 [published Online First: 2011/08/11]

10. Fenner L, Brinkhof MW, Keiser O, et al. Early mortality and loss to follow-up in HIV-infected children starting antiretroviral therapy in Southern Africa. *Journal of acquired immune deficiency syndromes (1999)* 2010;54(5):524.

11. Brophy JC, Hawkes MT, Mwinjiwa E, et al. Survival Outcomes in a Pediatric Antiretroviral Treatment Cohort in Southern Malawi. *PLoS One* 2016;11(11):e0165772. doi: 10.1371/journal.pone.0165772 [published Online First: 2016/11/05]

12. Htun T, Kyaw K, Aung T, et al. Attrition during pre-ART and ART time periods among adolescents enrolled in Integrated HIV Care Programme in Myanmar, 2005–2017. *Epidemiology & Infection* 2019;147

13. Organization WH. Guidelines for managing advanced HIV disease and rapid initiation of antiretroviral therapy, July 2017. 2017

14. Rosen S, Fox MP, Larson BA, et al. Accelerating the uptake and timing of antiretroviral therapy initiation in sub-Saharan Africa: an operations research agenda. *PLoS medicine* 2016;13(8):e1002106.

15. Organization WH. Antiretroviral therapy of HIV infection in infants and children: towards universal access: recommendations for a public health approach-2010 revision: World Health Organization 2010.

16. Minn AC, Kyaw NTT, Aung TK, et al. Attrition among HIV positive children enrolled under integrated HIV care programme in Myanmar: 12 years cohort analysis. *Global health action* 2018;11(1):1510593.

Confidential: For Review Only

17. Mutanga JN, Mutembo S, Ezeamama AE, et al. Long-term survival outcomes of HIV infected children receiving antiretroviral therapy: an observational study from Zambia (2003–2015). *BMC public health* 2019;19(1):1-12.

18. Demeke Dejen DJ, Yeshanew F, Fentaw Z, et al. Attrition and Its Predictors Among Adults Receiving First-Line Antiretroviral Therapy in Woldia Town Public Health Facilities, Northeast Ethiopia: A Retrospective Cohort Study. *HIV/AIDS (Auckland, NZ)* 2021;13:445.

19. Makurumidze R, Mutasa-Apollo T, Decroo T, et al. Retention and predictors of attrition among patients who started antiretroviral therapy in Zimbabwe's national antiretroviral therapy programme between 2012 and 2015. *PloS one* 2020;15(1):e0222309.

20. Onubogu CU, Ugochukwu EF. A 17 year experience of attrition from care among HIV infected children in Nnewi South-East Nigeria. *BMC Infectious Diseases* 2021;21(1):1-11.

21. Hassan AS, Mwaringa SM, Ndirangu KK, et al. Incidence and predictors of attrition from antiretroviral care among adults in a rural HIV clinic in Coastal Kenya: a retrospective cohort study. *BMC Public Health* 2015;15(1):1-9.

22. Decroo T, Koole O, Remartinez D, et al. Four-year retention and risk factors for attrition among members of community ART groups in T ete, M ozambique. *Tropical Medicine & International Health* 2014;19(5):514-21.

23. Teeraananchai S, Kerr SJ, Puthanakit T, et al. Attrition and mortality of children receiving antiretroviral treatment through the universal coverage health program in Thailand. *The Journal of pediatrics* 2017;188:210-16. e1.

24. Cohen S, Smit C, van Rossum AM, et al. Long-term response to combination antiretroviral therapy in HIV-infected children in the Netherlands registered from 1996 to 2012. *Aids* 2013;27(16):2567-75.

Confidential: For Review Only

25. Bimer KB, Sebsibe GT, Desta KW, et al. Incidence and predictors of attrition among children attending antiretroviral follow-up in public hospitals, Southern Ethiopia, 2020: a retrospective study. *BMJ paediatrics open* 2021;5(1)

26. Desai SN, Landay AL. HIV and aging: role of the microbiome. *Current Opinion in HIV and AIDS* 2018;13(1):22-27.

27. Frigati L, Archary M, Rabie H, et al. Priorities for decreasing morbidity and mortality in children with advanced HIV disease. *Clinical Infectious Diseases* 2018;66(suppl_2):S147-S51.

28. Fouda GG, De Paris K, Levy O, et al. Immunological mechanisms of inducing HIV immunity in infants. *Vaccine* 2020;38(3):411-15.

29. Mugglin C, Wandeler G, Estill J, et al. Retention in care of HIV-infected children from HIV test to start of antiretroviral therapy: systematic review. *PLoS One* 2013;8(2):e56446. doi: 10.1371/journal.pone.0056446 [published Online First: 2013/02/26]

30. Apondi E, Humphrey JM, Sang E, et al. Trends Over Time for Adolescents Enrolling in HIV Care in Kenya, Tanzania, and Uganda From 2001-2014. *J Acquir Immune Defic Syndr* 2018;79(2):164-72. doi: 10.1097/qai.0000000000001796 [published Online First: 2018/07/10]

31. Pantelic M, Casale M, Cluver L, et al. Multiple forms of discrimination and internalized stigma compromise retention in HIV care among adolescents: findings from a South African cohort. *Journal of the International AIDS Society* 2020;23(5):e25488.

32. Lankowski AJ, Siedner MJ, Bangsberg DR, et al. Impact of geographic and transportation-related barriers on HIV outcomes in sub-Saharan Africa: a systematic review. *AIDS and Behavior* 2014;18(7):1199-223.

Confidential: For Review Only

33. Carlos S, Burgueño E, Ndarabu A, et al. Predictors of retention in the prospective HIV prevention OKAPI cohort in Kinshasa. *Scientific Reports* 2021;11(1):1-10.

34. Geng EH, Bangsberg DR, Musinguzi N, et al. Understanding reasons for and outcomes of patients lost to follow-up in antiretroviral therapy programs in Africa through a sampling-based approach. *Journal of acquired immune deficiency syndromes (1999)* 2010;53(3):405.

35. Krauss BJ, Letteney S, Okoro CN. Why tell children: A synthesis of the global literature on reasons for disclosing or not disclosing an HIV diagnosis to children 12 and under. *Frontiers in Public Health* 2016;4:181.

36. Dahourou DL, Gautier-Lafaye C, Teasdale CA, et al. Transition from paediatric to adult care of adolescents living with HIV in sub-Saharan Africa: challenges, youth-friendly models, and outcomes. *Journal of the International AIDS Society* 2017;20:21528.

37. Bigna JJ, Plottel CS, Koulla-Shiro S. Challenges in initiating antiretroviral therapy for all HIV-infected people regardless of CD4 cell count. *Infect Dis Poverty* 2016;5(1):85. doi: 10.1186/s40249-016-0179-9 [published Online First: 2016/09/07]

Confidential: For Review Only

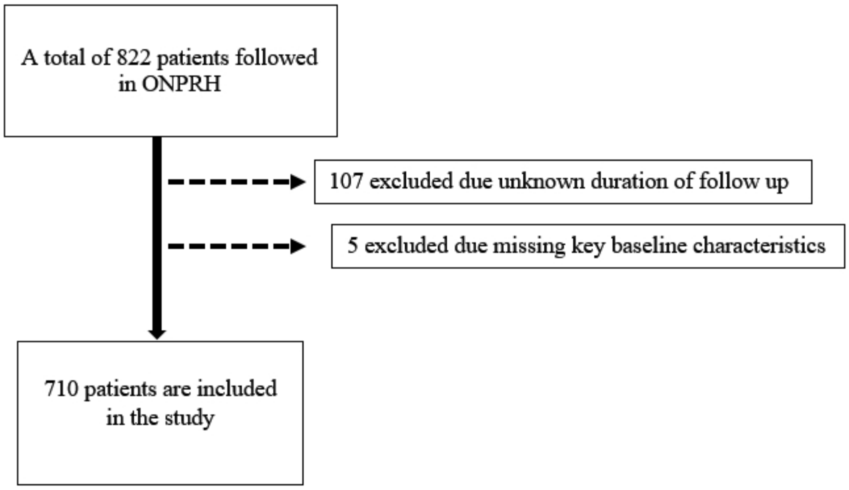

508x258mm (38 x 38 DPI)

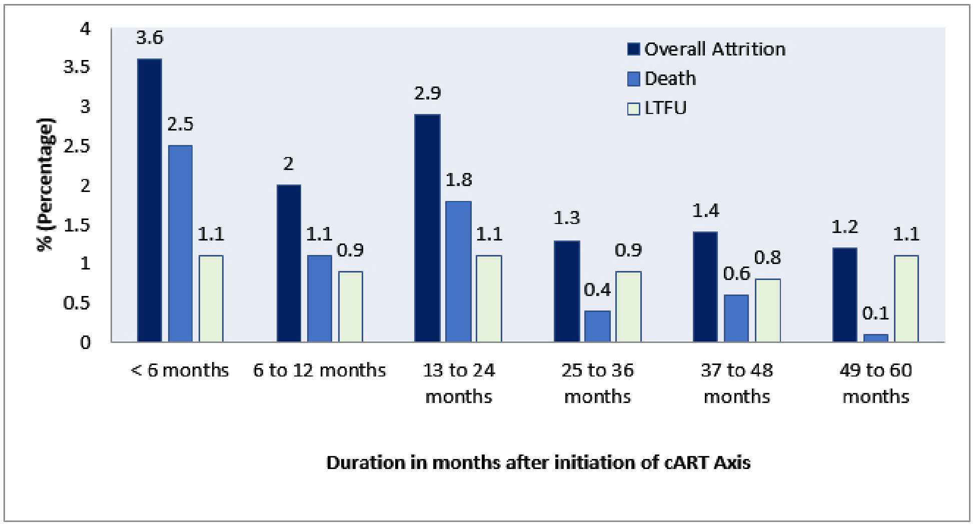

455x249mm (38 x 38 DPI)

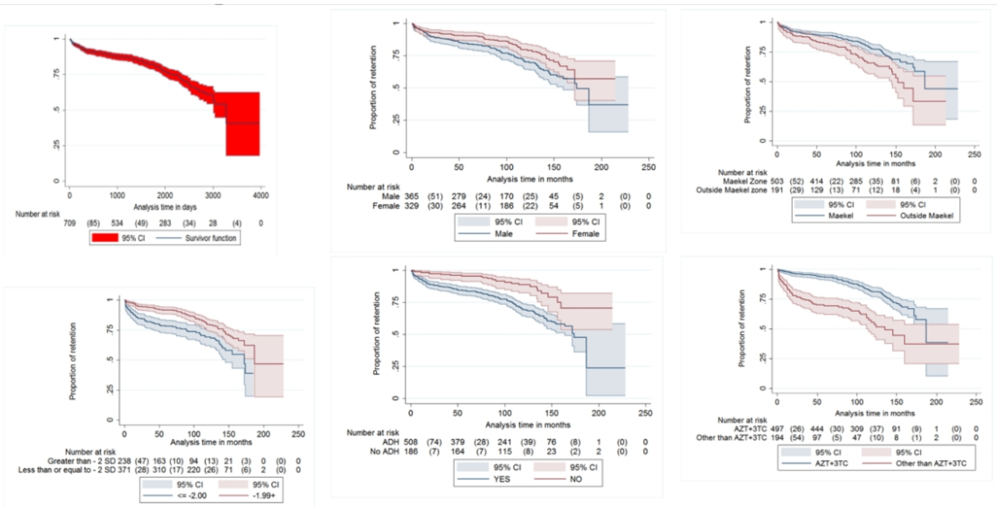

784x394mm (38 x 38 DPI)

BMJ Paediatrics Open

Attrition and associated factors among children living with HIV at a tertiary hospital in Eritrea: A retrospective cohort analysis

Journal:	*BMJ Paediatrics Open*
Manuscript ID	bmjpo-2022-001414.R2
Article Type:	Original research
Date Submitted by the Author:	12-Jun-2022
Complete List of Authors:	Mengistu, Samuel; Nakfa Hospital, General Medicine; Orota School of Medicine and Dentistry, Medicine<br>Ghebremeskel, Ghirmay; Nakfa Hospital, General Medicine<br>Rezene, Aron; Edaga Hamus Hospital, Ministry of health Maekel branch, Asmara, Eritrea<br>Idris, Mahmud ; Department of pediatrics and child health, Orotta College of Medicine and Health Sciences, Asmara, Eritrea<br>Tikue, Tsegereda; Department of pediatrics and child health, Orotta College of Medicine and Health Sciences, Asmara, Eritrea<br>Hamida, Mohammed ; Unit of Medical Microbiology, Orotta College of Medicine and Health Sciences (OCMHS).<br>Achila, Oliver ; Unit of Clinical Laboratory Science, Orotta College of Medicine and Health Sciences (OCMHS).
Keywords:	Virology, HIV, Mortality, Epidemiology

SCHOLARONE™
Manuscripts

BMJ

I, the Submitting Author has the right to grant and does grant on behalf of all authors of the Work (as defined in the below author licence), an exclusive licence and/or a non-exclusive licence for contributions from authors who are: i) UK Crown employees; ii) where BMJ has agreed a CC-BY licence shall apply, and/or iii) in accordance with the terms applicable for US Federal Government officers or employees acting as part of their official duties; on a worldwide, perpetual, irrevocable, royalty-free basis to BMJ Publishing Group Ltd ("BMJ") its licensees and where the relevant Journal is co-owned by BMJ to the co-owners of the Journal, to publish the Work in this journal and any other BMJ products and to exploit all rights, as set out in our licence.

The Submitting Author accepts and understands that any supply made under these terms is made by BMJ to the Submitting Author unless you are acting as an employee on behalf of your employer or a postgraduate student of an affiliated institution which is paying any applicable article publishing charge ("APC") for Open Access articles. Where the Submitting Author wishes to make the Work available on an Open Access basis (and intends to pay the relevant APC), the terms of reuse of such Open Access shall be governed by a Creative Commons licence – details of these licences and which Creative Commons licence will apply to this Work are set out in our licence referred to above.

Other than as permitted in any relevant BMJ Author's Self Archiving Policies, I confirm this Work has not been accepted for publication elsewhere, is not being considered for publication elsewhere and does not duplicate material already published. I confirm all authors consent to publication of this Work and authorise the granting of this licence.

Confidential: For Review Only

What's known about the subject?

Attrition and associated factors among children living with HIV at a tertiary hospital in Eritrea: A retrospective cohort analysis

Samuel Tekle Mengistu[1*], Ghirmay Ghebrekidan Ghebremeskel[2], Aron Rezene[3], Mahmud Mohammed Idris[4], Tsegereda Gebrehiwot Tikue[5], Mohammed Elfatih Hamida[6], Oliver Okoth Achila[7]

[1]**Samuel Tekle Mengistu**. Nakfa Hospital, Ministry of health Northern Red Sea branch, Nakfa, Eritrea. Email: teklesam7@gmail.com

[2]**Ghirmay Ghebrekidan Ghebremeskel**. Nakfa Hospital, Ministry of health Northern Red Sea branch, Nakfa, Eritrea. Email: GhirmayGGG@gmail.com

[3]**Aron Rezene**. Edaga Hamus Hospital, Ministry of health Maekel branch, Asmara, Eritrea Email: aronrezene19@gmail.com

[4]**Mahmud Mohammed Idris.** Department of pediatrics and child health, Orotta College of Medicine and Health Sciences, Asmara, Eritrea. Email: mahmudfil@yahoo.com

[5]**Tsegereda Gebrehiwot Tikue.** Department of pediatrics and child health, Orotta College of Medicine and Health Sciences, Asmara, Eritrea. Email: proftsigeghebrehiwet@gmail.com

[6]**Mohammed Elfatih Hamida.** Unit of Medical Microbiology, Orotta College of Medicine and Health Sciences (OCMHS). Email: mohelfatih77@gmail.com

[7]**Oliver Okoth Achila.** Unit of Clinical Laboratory Science, Orotta College of Medicine and Health Sciences (OCMHS). Email: oliverachila@gmail.com

*Corresponding author: **Samuel Tekle Mengistu**, email: teklesam7@gmail.com, Nakfa Hospital, Northern Red Sea Ministry of Health, Asmara, Eritrea.

Word count: 5,285

Confidential: For Review Only

Studies in low- and medium-income countries (LMICs) of SSA and Asia have demonstrated that retention of children living with HIV (CLHIV) in care is a major challenge. Multiple evaluations have suggested that in the context of the rapid scale-up of cART, reducing attrition (Lost to follow-up [LTFU] and mortality) rate is a key programmatic priority.

What the study adds?

- A quarter of the patients weren't retained in care.
- Attrition in the first two years after enrollment was largely due to death.
- Attrition was mostly associated with enrollment in recent years; malnutrition; advanced HIV disease (III or IV) at enrollment; initiation of backbones other than AZT+3TC; and residence outside Zoba Maekel.

How this study might affect research, practice or policy

- Our findings emphasis on the need of linked databases across the HIV/AIDS care cascade within Eritrea.
- It also highlights the importance of enhanced outreach and intensive case-finding/tracing for LTFU; as standard components of HIV/AIDS care programs in Eritrea are equally evident.

ABSTRACT

Confidential: For Review Only

Background: Reducing attrition in paediatric HIV-positive patients using combined antiretroviral therapy (cART) programs in sub-Saharan Africa (SSA) is a challenge. This study explored the rates and predictors of attrition in children started on cART in Asmara, Eritrea.

Methods: This was a retrospective cohort study using data from all paediatric patients on cART between 2005 and 2020, conducted at the Orotta National Referral and Teaching Hospital. Kaplan–Meier estimates of the likelihood of attrition and multivariate Cox proportional hazard models were used to assess the factors associated with attrition. All *p*-values were two-sided and $p<0.05$ was considered statistically significant.

Results: The study enrolled 710 participants with 374 males (52.7%) and 336 females (47.3%). After 5364 person-years follow-up, attrition occurred in 172 (24.2%) patients: 65 (9.2%) died and 107 (15.1%) were lost to follow-up (LTFU). The crude incidence rate of attrition was 3.2 events/100 person-years (PY), mortality rate was 2.7/100 PY, and LTFU was 1.2/100 PY. The independent predictors of attrition included male sex (AHR=1.6, 95% confidence interval, (CI): 1–2.4), residence outside Zoba Maekel (AHR=1.5, 95% CI: 1–2.3), later enrolment years (2010–2015: AHR=3.2, 95% CI: 1.9–5.3; >2015: AHR=6.1, 95% CI: 3–12.2), WHO BMI for age- Z-score (BAZ) <-2 (AHR=1.4, 95% CI: 0.9–2.1), advanced HIV disease (WHO III or IV) at enrolment (AHR=2.2, 95% CI: 1.2–3.9), and initiation of Zidovudine+Lamivudine (AZT+3TC) or other cART backbones (UHR=2, 95% CI: 1.2–3.2). In contrast, a reduced likelihood of attrition was observed in children with a record of cART changes (UHR=0.2, 95% CI: 0.15–0.4).

Conclusion: A low incidence of attrition was observed in this study. However, the high mortality rate in the first 24 months of treatment and late presentation are concerning. Therefore, data-driven interventions for improving program quality and outcomes should be prioritized.

Key words: *Risk Factors, Pediatrics, HIV/AIDS, Attrition, lost to follow-up, Eritrea*

INTRODUCTION

Confidential: For Review Only

Despite advancements in HIV management, HIV infection in children remains a major public health burden. A recent Joint United Nations Programme on HIV/AIDS (UNAIDS) report suggested that of the 37.7 million [30.2–45.1 million] people living with HIV (PLWH) in 2020; Children <14 years' account for ~1.8 million [1.3–2.2 million].[1] In the same period, 180,000 children were newly infected and 110,000 children died of HIV/AIDS. Importantly, a disproportionate number of these children were from the sub-Saharan Africa (SSA), where resources are limited, and the burden of infectious disease is high.

The prevailing situation undermines the aspirational UNAIDS 90-90-90 target to end the HIV/AIDS epidemic by 2030 (Project 2030).[1] Although combined antiretroviral therapy (cART) helps PLWH live long and healthy lives, and marked improvements have been registered in almost all principal landmarks in the HIV care cascade globally, empirical data from low- and medium-income countries (LMICs) in SSA and Asia suggest that children living with HIV (CLHIV), particularly young adolescents, still experience increasing HIV/AIDS-related mortality.[2] Multiple studies in LMIC settings also demonstrated that a large proportion of CLHIV continue to present to care centres with advanced HIV disease, and that retention is a major challenge.[2-4] Hence, emerging reports from the region point (sensu stricto) to significant problems in the fast-track UNAIDS 90-90-90s: diagnosis (early) - treat (promptly) - retain (to ensure viral suppression) paradigm, thereby putting targeted timelines off track.[1]

Responses to this problem call for precise rapid scale-up of cART and reduction of attrition (lost to follow-up [LTFU] and mortality) in LMIC HIV/AIDS programs. This suggestion is based from data showing that attrition is high among younger (children <14 years), approximately 14% at 12 months follow-up and 22% at 36 months.[5 6] Furthermore, some experts argue that reducing attrition rates is not only necessary for continued cART, but it also allows clinicians to evaluate the emergence of medication toxicity. It also gives them the opportunity to prevent, manage, or treat opportunistic infections (IOs), detect treatment failure, and initiate prompt switching of cART. Additional benefits include ancillary services such as social support and counselling. In this regard, studies on attrition and associated factors

Confidential: For Review Only

among CLHIV in jurisdictions across SSA should be a principal research concern in the foreseeable future.

Therefore, we aimed to describe the incidence of attrition and associated factors among CLHIV in Asmara, Eritrea, a country with one of the lowest HIV prevalence in SSA.[7] Data gathered from this study can be used as a benchmark for gauging successes and weaknesses of programs,[5] and to identify critical gaps and opportunities for context-specific interventions aimed at minimizing attrition and maximizing retention. We also explored the multiple temporal trends associated with program performance, mortality, and enrolment rates.

METHODOLOGY

Study design and setting

This retrospective cohort study was conducted in the paediatric HIV/AIDS follow-up clinic at the National Pediatric Referral Hospital (NPRH). The HIV/AIDS follow-up clinic in NPRH was commissioned in 2005, making it the first institution in Eritrea to offer cART for CLHIV. Before the decentralisation of services to other zones (2010), NPRH (in the Maekel zone) was the only institution in the country offering cART to CLHIV. In total, 822 children aged below 15 years received service and/or had been enrolled at the clinic since their inception.

Data collection and approach

We used the data collected from the database of the ONRTH paediatric cART and follow-up program (cART DHIS 2). The retrieved data were robustly reviewed and cross-referenced with patient clinical chart registries. The data retrieved from this repository were exported to Microsoft (MS) Excel. Baseline data collected included the date of birth/age at enrolment, sex, address, cART initiation date, initial cART regimen, presence of tuberculosis (TB), baseline WHO clinical stage, baseline CD4 count, weight, haemoglobin concentration, and complete blood count. Height is not available in electronic medical records and was thus retrieved from clinical chart registries. The WHO standard deviations for

Confidential: For Review Only

weight for age, height for age, and body mass index (BMI) were computed using the WHO standard deviation for emergency nutritional assessment software (www.nutrisurvey.net/ena/ena.html). The recruitment details are illustrated in Figure 1.

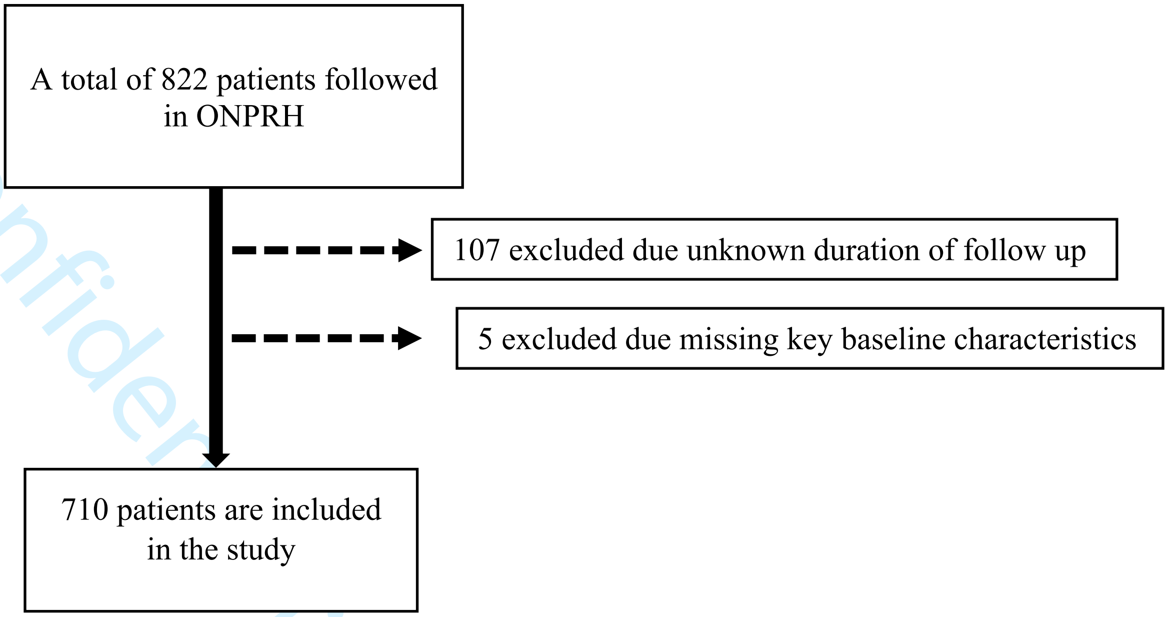

Figure 1: Flow chart for study participant recruitment.

Operational Definitions

- Death was defined as deaths occurring after enrolment in follow-up but before 31 December 2020.[8-10]
- Patients were considered as LTFU if they had missed scheduled visits for ≥ 3 consecutive months.[8]
- Attrition was defined as LTFU or death during follow-up in the clinic.[5 11]
- Retention: was defined as children who were alive and on follow-up in the clinic at the end of the study period and children who were transferred out at any time during the study period.[11]
- Advanced HIV disease was defined as a CD4 cell count <200 cells/mm^3 and/or WHO stage 3 or 4 events for patients >5 years of age.[12]

Confidential: For Review Only

- Late presentation was defined as patients enrolled with advanced WHO HIV stage (III/IV) and/or T-cell CD4+ count <200 cells/μL.
- Multiple measures were used to measure the nutritional status. These included the weight-for-age Z-score (WAZ), height-for-age (HAZ), and BMI-for-age Z-score (BAZ). For nutrition, BAZ underscores <-2 Standard deviation, SD, WAZ ≤-2 z-score, and/or HAZ ≤-2 z-score.
- Residence outside Zoba Maekel was defined children who reside outside Zoba Maekel, the administrative zone of Eritrea.
- Adherence was assessed at each follow-up visit as good, fair, and poor if a child missed <5 %, <10%, and >10% doses respectively of the expected monthly doses.

Data analysis

The retrieved data were exported to IBM SPSS 26.0 (SPSS Inc., Chicago, Illinois, USA.) and STATA version 12.0 (STATA Corporation, College Station, TX), where they were processed for analysis. Descriptive statistics for categorical variables were analyzed using the chi-square or Fisher's exact test and summarized using frequency and percentage. Depending on the data distribution, quantitative data were summarized using mean (±SD) or median (Interquartile range, IQR). Suitable parametric or nonparametric statistics were used to evaluate differences. Incidence of attrition was calculated per 100 person-years (PY), (95% confidence interval, [CI]). Furthermore, Kaplan–Meier curves describe and compare retention and survival at different follow-up intervals by sex, address, cohort year, adjusted hazard ratio (AHD), initial cART backbone, and record of cART change. The log-rank test was used to evaluate differences in the equality of survival functions. All LTFU and transfer-outs were censored on the date of their last visit. Estimates of hazard ratios for attrition, 95% CI, and quantification of associations were evaluated using Cox proportional hazards models. Log-log plots and plots of Schoenfeld residuals were used to evaluate the proportional hazards assumption. To adjust for possible confounders, multivariate Cox regression models were used. The final results are presented as adjusted

https://mc.manuscriptcentral.com/bmjpo

Confidential: For Review Only

hazard risk (AHR) with 95% CI. All p-values were two-tailed and p-values <0.05 were considered statistically significant.

Ethical consideration

Ethical approval was obtained from the Ministry of Health (MOH) Research Ethics and Protocol Review Committee (letter of reference:02/05/21). All data collected from the respondents' records were kept confidential. Consent to participate was not obtained from the patients or guardians because the study was based on anonymized patient records, which was waived by the ethical committee. All study procedures followed the recommendations of the Declaration of Helsinki Convention.

Patient and public involvement

Patients or the public WERE NOT involved in the design, conduct, reporting, or dissemination plans of our research.

RESULTS

From September 2005 to December 2020, 822 CLHIV were enrolled in the ONPRH paediatric cART treatment and follow-up program. After evaluating eligibility, 710 (86.3%) participants were included in the analysis. Stratification of enrolment with respect to specific time frames showed that 480(67.6%), 180(25.4%), and 50(7.0%) patients were enrolled to care between 2005–2010; 2011–2015; and 2016–2020, respectively (Table 1). Eligible participants were followed up for 5,364 person-years follow-up (PYFU). During this period, 65 (9.2%) died, 107 (15.1%) were LTFU, 153 (21.5%) were transferred out, and 385 (54.2%) were currently receiving treatment.

Table 1. Baseline characteristics of HIV-infected children and adolescents in the ONPRH cART treatment center, Asmara, Eritrea (2005–2020).

Patients' characteristics	Total N (%)	2005-2010	2011-2015	2016-2020	P-value ($\chi 2$)
**Number enrolled**	710	480(67.6)	180(25.4)	50(7.0)	
**Gender**					
Male	374 (52.7)	237 (63.4)	111 (29.7)	26 (7)	**0.01 (7.9)**
Female	336 (47.3)	243 (72.3)	69 (20.5)	24 (7.2)	
**Age on enrollment in years, median (IQR)**	6 (3-9)	5 (3-8)	7 (4-10)	5 (2-11)	**0.001[a]**
<=5 years	274 (38.6)	198 (72.3)	52 (19)	24 (8.7)	**<0.001 (27.5)**
6-10 years	284 (40)	202 (71.1)	72 (25.4)	10 (3.5)	

Confidential: For Review Only

>10 years	152 (21.4)	80 (52.6)	56 (36.8)	16 (10.6)	
**Address**					
Maekel	516 (72.7)	358 (69.4)	124 (24)	34 (6.6)	0.25 (2.7)
Outside Maekel	194 (27.3)	122 (62.9)	56 (28.9)	16 (8.2)	
**Disease stage**					
Early	191(26.9)	134 (70.2)	46 (24)	11 (5.8)	0.5 (1)
Advanced	519 (73.1)	346 (66.7)	134 (25.8)	39 (7.5)	
**TB- status**					
Symptomatic and under treatment	16 (2.4)	7 (43.8)	6 (37.5)	3 (18.8)	0.5 (5.9)
Not symptomatic	625 (97.6)	443 (70.9)	135 (21.6)	47 (7.5)	
**CD4 count, Median (IQR)**	274 (150-442)	265 (151-392.7)	307 (137-555)	340 (200-888)	**0.012[a]**
**CD4 percentage, Median (IQR)**	12 (7.4-17)	11.1 (7.1-16.5	13.6 (9-21)	14.3 (7.5-21.9)	**0.007[a]**
**Hemoglobin, mean(±SD)**	11.8(±13.5)	12.5 (±17.2)	10.6 (±2.1)	11 (±1.6)	**0.060[a]**
Anemia	264 (45.7)	155 (58.7)	89 (33.7)	20 (7.6)	0.3 (2.2)
No Anemia	313 (54.3)	197 (62.9)	88 (28.2)	28 (8.9)	
**WAZ, median (IQR)**	-2.6 (-3.6- -1.7)	-2.5 (-3.5- -1.7)	-2.8 (-3.8- -1.7)	-2.9 (-3.8-1.6)	0.263[a]
WAZ=>-2	141 (31.9)	98 (69.5)	32 (22.7)	11 (7.8)	0.7 (0.5)
WAZ <-2	302 (68.1)	202 (66.9)	78 (25.8)	22 (7.3)	
**HAZ, median (IQR)**	-2.75 (-3.8 - -1.7)	-2.6 (-3.7 -- -1.5)	-2.9 (-4 - -1.8)	-3(-3.9 - -1.8)	0.063[a]
HAZ=>-2	223 (32.7)	161 (72.2)	49 (22)	13 (5.8)	0.1 (4.3)
HAZ <-2	461 (67.3)	296 (64.2)	129 (28)	36 (7.8)	
**BAZ, median (IQR)**	-1.58 (-2.8--0.69)	-1.5 (-2.9--0.5)	-1.6 (-2.7--1)	-1.7 (-3--0.6)	0.385[a]
BAZ=>-2	378 (60.4)	257 (68)	97 (25.7)	24 (6.3)	
BAZ <-2	247 (29.6)	162 (65.6)	64 (25.9)	21 (8.5)	0.5 (1)
**cART backbone**					
AZT+3TC	508 (71.7)	347 (68.3)	148 (29.1)	13 (2.6)	
Other backbones	201 (28.3)	133 (66.2)	31 (15.4)	37 (18.4)	**<0.001 (62)**
**NNRTI**					
EFV	366 (51.6)	262 (71.6)	90 (24.6)	14 (3.8)	**<0.001 (13)**
NVP	340 (48.4)	216 (63.5)	88 (25.8)	36 (10.6)	
**Current cART regimen**					
First line cART	633 (89.2)	429 (67.8)	157 (24.8)	47 (7.4)	0.3 (1.9)
Second line cART	77 (10.8)	51 (66.2)	23 (29.9)	3 (3.9)	
**Adherence**					
Suboptimal Adherence	91 (12.3)	46 (50.5)	39 (42.9)	6 (6.6)	**<0.001 (17)**
No record	619 (87.2)	434 (70)	141 (22.8)	44 (7.2)	
**cART change**					
cART changed at least once	516 (75)	**340 (65.9)**	148 (28.7)	28 (5.4)	**<0.001 (1.2)**
No cART change	172 (25)	120 (69.8)	30 (17.4)	22 (12.8)	
Duration of follow up in years, Median (IQR)	8 (4-11)	10 (7-12)	6 (4-8)	2 (1-3.25)	**<0.001[a]**

Abbreviations: cART; combined antiretroviral therapy, NNRTI; non-nucleoside reverse transcriptase inhibitor, WAZ; weight for age standard deviations, HAZ; height for age standard deviations, and BAZ; Body mass index for age standard deviations

Definitions: **Other back bones** refer to Tenofovir disoproxil fumarate (TDF) + Emtricitabine (FTC), Abacavir (ABC) + 3TC or Stavudine (D4T) + 3TC. **Anemia** was defined as hemoglobin (Hg) level is < 11 g/dl for children <5 years old, <11.5 g/dl for children 5–11.9 years old, and <12 g/dl for children >12 years old.

Superscript: a; Mann-Whitney u test.

Comparisons of proportions were performed by using the χ2 test, medians by using Mann-Whitney tests (a).

Sociodemographic characteristics of CLHIV

The median (IQR) age at enrolment in the clinic was 6 years (IQR: 3–9 years). The median (IQR) ages from 2005–2010; 2010–2015, and 2016–2020 were 5 years (IQR: 3–8 years), 7 years (IQR: 4–10 years), and 5 years (IQR: 6.25–12.75 ears), respectively. In terms of sex, the numbers of males and females

Confidential: For Review Only

were similar (374 [52.7%] vs 336 [47.3%], respectively). Most of the participants were from the Maekel zone (72.7%).

Clinical and disease-related characteristics of CLHIV at baseline

Assessment of clinic-level factors showed that the median CD4 count and percentage at treatment initiation were 274 cells/µl (IQR, 150–442 cells/µl) and 12% (7.4–17%), respectively. In the relevant time frames, the median (IQR) values were 265 cells/µl (151–392.7) cells/µl in 2005–2010; 307 cells/µl (137–555) cells/µl in 2010–2015; and 340 cells/µl (200–888) cells/µl in 2016–2020, (Mann–Whitney test, p =0.012). Similarly, the median (IQR) for CD4 percentages in 2005–2010; 2010–2015; and 2016–2020 were 11.1% (7.1–16.5%), 13.6% (9–21%), and 14.3% (7.521.9%), respectively (Mann–Whitney test, p = 0.007). Overall, and in the following order, 519(73.1%); 264(45.7%); and 16(2.4%) patients had advanced (STAGE III or IV) disease, anemia, and TB before initiating cART, respectively. The proportion of children with WAZ z-score <-2 SD (underweight), HAZ z-score <-2 SD (stunted), and BAZ z-score <-2 SD (undernourished) were 302 (68.1%); 461 (67.3%), and 247 (29.6%), respectively. Despite differences in enrolment in the successive time frames, no improvements were observed in these growth parameters [anaemia, WAZ, HAZ, and BAZ] (Table 1).

Treatment-related characteristics and outcomes

The preferred cART backbone was AZT+3TC (71.7%). Suboptimal adherence was recorded in 12.3% of patients, while most (72%) participants had a history of cART change at least once.

Prevalence of retention and attrition

Overall attrition (death + LTFU), death, and LTFU are presented in Figure 2. Based on the results, the overall death rate and LTFU were 2.7 [95% CI: 2.1–3.5]/100 PY and 1.2 [95% CI: 1–1.5]/100 PY. Attrition at (0 to ≤6 months, >6 to ≤12 months, >12 to ≤24 months, >24 to ≤36 months, >36 to ≤48 months, and >48 to ≤60 months of follow-up were 3.6%, 2%, 2.9%, 1.3%, 1.4%, and 1.2%, respectively.

https://mc.manuscriptcentral.com/bmjpo

Confidential: For Review Only

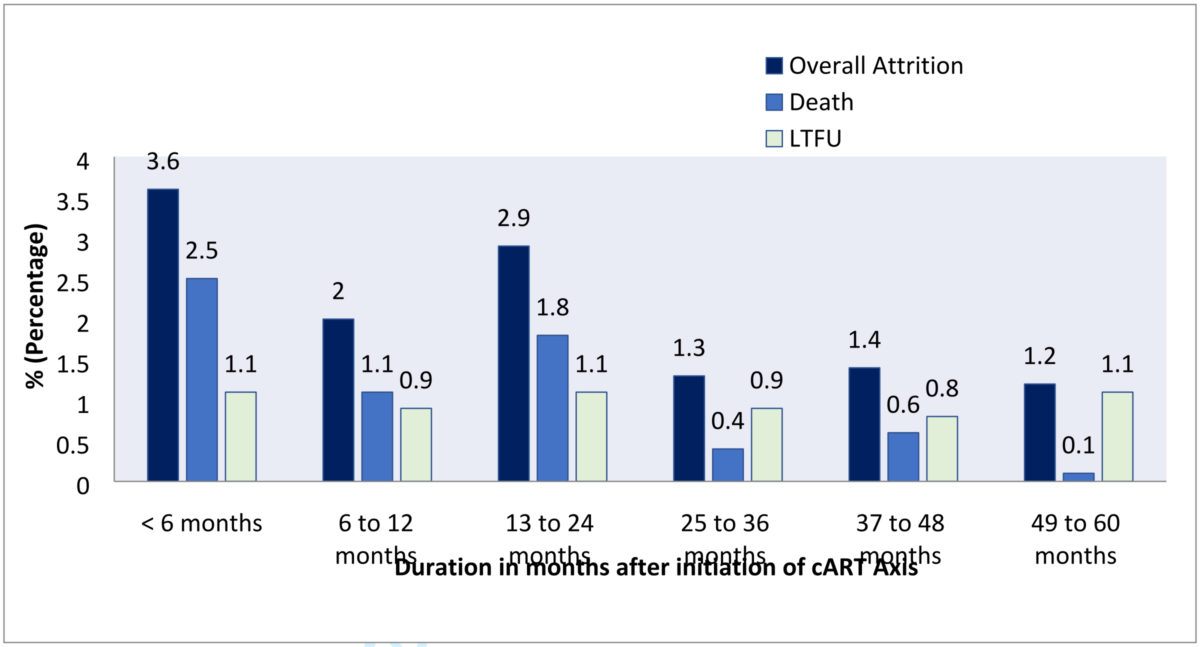

Figure 2. Frequency of attrition, deaths, and LTFU at specified time points within the first 60 months after initiation of cART

Factors Associated with retention and attrition

Out of 710 CLHIV included in the study, attrition occurred in 172 (24.2% [95% CI: 20–26.3]) patients. The crude incidence and overall survival duration were 3.2 events per 100 PY (95% CI: 2.7–3.7) and 14 years (95% CI: 13.4–14.8). Duration of follow-up in years for attrition and retention were 4.5 years (IQR: 1–9 years) and 9 years (IQR: 6–11 years), respectively. In terms of proportions, attrition rates were higher in males 104(27.8%) vs 68(20.2%) in females; residence outside Maekel 57[29.4%] vs 115[22.3%] in residence within Maekel; advanced HIV (WHO stage III and IV) 149(28.7) vs 23(12%) for early presentation; presence of anemia 72 [27.5%] vs 54 [17.3%] in no anemia; WAZ z-score <-2 (underweight) 83(27.5%) vs 27(19.1%) in WAZ z-score >-2; HAZ z-score <-2 (stunting) 122 (26.5%) vs 42(18.8%) in HAZ z-score > -2; BAZ score <-2 (Undernourished) 73[29.6%] vs 76[20.1%] in BAZ z-score >-2; cART other than AZT+3TC backbone 71(35.3%) vs 101(19.9%) in AZT + 3TC; No record of cART change or switching 77(44.8%) vs 92(17.8%) [Table 2].

Table 2. Characteristics of the study participants stratified by survival outcome in the ONPRH cART treatment center, Asmara, Eritrea (2005–2020).

Abbreviations: cART; combined antiretroviral therapy, NNRTI; non-nucleoside reverse transcriptase inhibitor, WAZ; weight for age standard deviations, HAZ; height for age standard deviations, and BAZ; Body mass index for age standard deviations

Cohort Characteristics	Total n (%)	Attrition n (%)	Retention n (%)	P-value ($\chi 2$)
**Gender**				
Male	374 (52.7)	104 (27.8)	270 (72.2)	**0.01 (5.5)**
Female	336 (47.3)	68 (20.2)	268 (79.8)	
**Age on enrollment in years, median (IQR)**	6 (3-9)	6 (3-10)	6 (3-9)	0.38[a]
<=5 years	274 (38.6)	62 (22.6)	212 (77.4)	0.09 (4.7)
6-10 years	284 (40)	63 (22.2)	221 (77.8)	
>10 years	152 (21.4)	47 (30.9)	105 (69.1)	
**Enrollment Year, Median (IQR)**				0.24[a]
2005-2010	265 (37.3)	111 (23.1)	369 (76.9)	0.23 (2.9)
2011-2015	289 (40.7)	44 (24.4)	136 (75.6)	
2016-2020	156 (22)	17 (34)	33 (66)	
**Address**				
Maekel	516 (72.7)	115 (22.3)	401 (77.7)	**0.049 (3.8)**
Outside Maekel	194 (27.3)	57 (29.4)	137 (70.6)	
**Disease stage**				
Early	191(26.9)	23 (12)	168 (88)	**<0.001 (21)**
Advanced	519 (73.1)	149 (28.7)	370 (71.3)	
**TB- status**				
Symptomatic and under treatment	16 (2.4)	5 (31.2)	11 (68.8)	0.5 (0.4)
Not symptomatic	625 (97.6)	151 (24.2)	474 (75.8)	
**CD4 count, Median (IQR)**	274 (150-442)	216 (100-371)	287 (163-452)	**<0.001[a]**
**CD4 percentage, Median (IQR)**	12 (7.4-17)	9.9 (6-14.5)	12.5 (8.2-19)	**<0.001[a]**
**Hemoglobin, Mean (±SD)**	11.8(±13.5)	10.7 (9.2-11.8)	11.2 (9.8-12.3)	**<0.001[b]**
Anemia	264 (45.7)	72 (27.5)	192 (72.5)	**0.004 (8.4)**
No Anemia	313 (54.3)	54 (17.3)	259 (82.7)	
**WAZ, median (IQR)**	-2.6 (-3.6--1.7)	-3 (-4.3- -2)	-2.5 (-3.5- --1.6)	**0.001[a]**
WAZ=>-2	141 (31.9)	27 (19.1)	114 (80.9)	**0.05 (3.5)**
WAZ <-2	302 (68.1)	83 (27.5)	219 (72.5)	
**HAZ, median (IQR)**	-2.75 (-3.8--1.7)	-3.2 (-4- -1.9)	-2.5 (3.7- --1.5)	**0.001[a]**
HAZ=>-2	223 (32.7)	42 (18.8)	181 (81.2)	**0.028 (4.8)**
HAZ <-2	461 (67.3)	122 (26.5)	339 (73.5)	
**BAZ, median (IQR)**	-1.58 (-2.8--0.69)	-1.9 (-3.6- -0.7)	-1.5 (-2.6- -0.6)	**0.012[a]**
BAZ=>-2	378 (60.4)	76 (20.1)	302 (79.9)	**0.007 (7.3)**
BAZ <-2	247 (29.6)	73 (29.6)	174 (70.4)	
**cART backbone**				
AZT+3TC	508 (71.7)	101 (19.9)	407 (80.1)	**<0.001 (18.6)**
Other backbones	201 (28.3)	71 (35.3)	130 (64.7)	
**NNRTI**				
EFV	366 (51.6)	91 (24.9)	275 (75.1)	0.67 (0.1)
NVP	340 (48.4)	80 (23.5)	260 (76.5)	
**Current cART regimen**				
First line cART	633 (89.2)	156 (24.6)	477 (75.4)	0.45 (0.5)
Second line cART	77 (10.8)	16 (20.8)	61 (79.2)	
**Adherence**				
Suboptimal Adherence	91 (12.3)	24 (26.4)	67 (73.6)	0.6 (0.2)
No record	619 (87.2)	148 (23.9)	471 (76.1)	
**cART change**	516 (75)	92 (17.8)	424 (82.2)	**<0.001 (50.5)**
No cART change	172 (25)	77 (44.8)	95 (55.2)	
Duration of follow up in years, Median (IQR)	8 (4-11)	4.5 (1-9)	9 (6-11)	**<0.001[a]**

Definitions: **Other back bones** refer to Tenofovir disoproxil fumarate (TDF) + Emtricitabine (FTC), Abacavir (ABC) + 3TC or Stavudine (D4T) + 3TC. **Anemia** was defined as hemoglobin (Hg) level is < 11 g/dl for children <5 years old, <11.5 g/dl for children 5–11.9 years old, and <12 g/dl for children >12 years old.

Superscript: a; Mann-Whitney u test. b; Independent sample t-test

Comparisons of proportions were performed by using the $\chi 2$ test, medians by using Mann-Whitney tests (a) and means using independent sample t-test.

Confidential: For Review Only

Kaplan–Meier analysis for Attrition incidence

Kaplan–Meier survival curves with log-rank tests were constructed to compare the cumulative incidence of attrition by sex, address, cohort year, AHD, initial cART backbone, and record of cART change (Figure 3 and Table 3). Overall, males had a shorter survival of 13 years (95% CI, 2–14 years) vs 13.8 years in females (95% CI: 13–14 years) [log rank test, $p = 0.008$] (Figure 2a). The mean survival duration was significantly shorter with increasing age at baseline: ≤5 years: 15.1(14.2–16) years vs 6–10 years: 11.7(11.1–12.2 years) and >10 years: 8.4(7.43–12.2 years) years (log rank test, $p <0.001$). Children who reside outside Maekel also have a significantly shorter mean survival of 11 years [95% CI: 10–11.7 years] in the clinic compared to those from Maekel with 14.5 years [95% CI: 13.8–15.4 years] (log rank test $p = 0.002$ [Figure 2b]). Late enrollment was also associated with shorter mean survival (2005–2009: 14.8 years [95% CI: 14–15.5 years] vs 2010–2014: 7.5 years [95% CI: 7–8 years] vs 2015–2020: 3.1 years [95% CI: 2.7–3.5 years] (log rank test $p = 0.008$) [Figure 2c]). Children with AHD at presentation had a significantly shorter mean survival time in ONPRH (13 years [95% CI: 12.3–14]) compared to those without (15 years [95% CI: 14.2–15.7 years]). The survival time for those taking treatment other than AZT+3TC (11.3 years [95% CI: 9.8–12.8 years]) was significantly shorter compared to the AZT+3TC group (14 years [95% CI: 13.4–14.5 years]) [log rank test $p < 0.001$] (Figure 2d). Moreover, children with a record of cART change had significantly longer mean survival time in the clinic (13 years [95% CI: 13–14 years]) as compared to those without (7.5 years [95% CI, 6.6–8.4 years]) [log rank test $p < 0.001$]. Other factors associated with shorter survival were: anemia at ART initiation (Haemoglobin <8 g/dl) [$p = 0.032$], WAZ score <-2 (12 years [11.4–12.8 years]); HAZ score <-2 (12.7 years [12–13.4 years]); BAZ score <-2 (12 years [11–13 years]).

Confidential: For Review Only

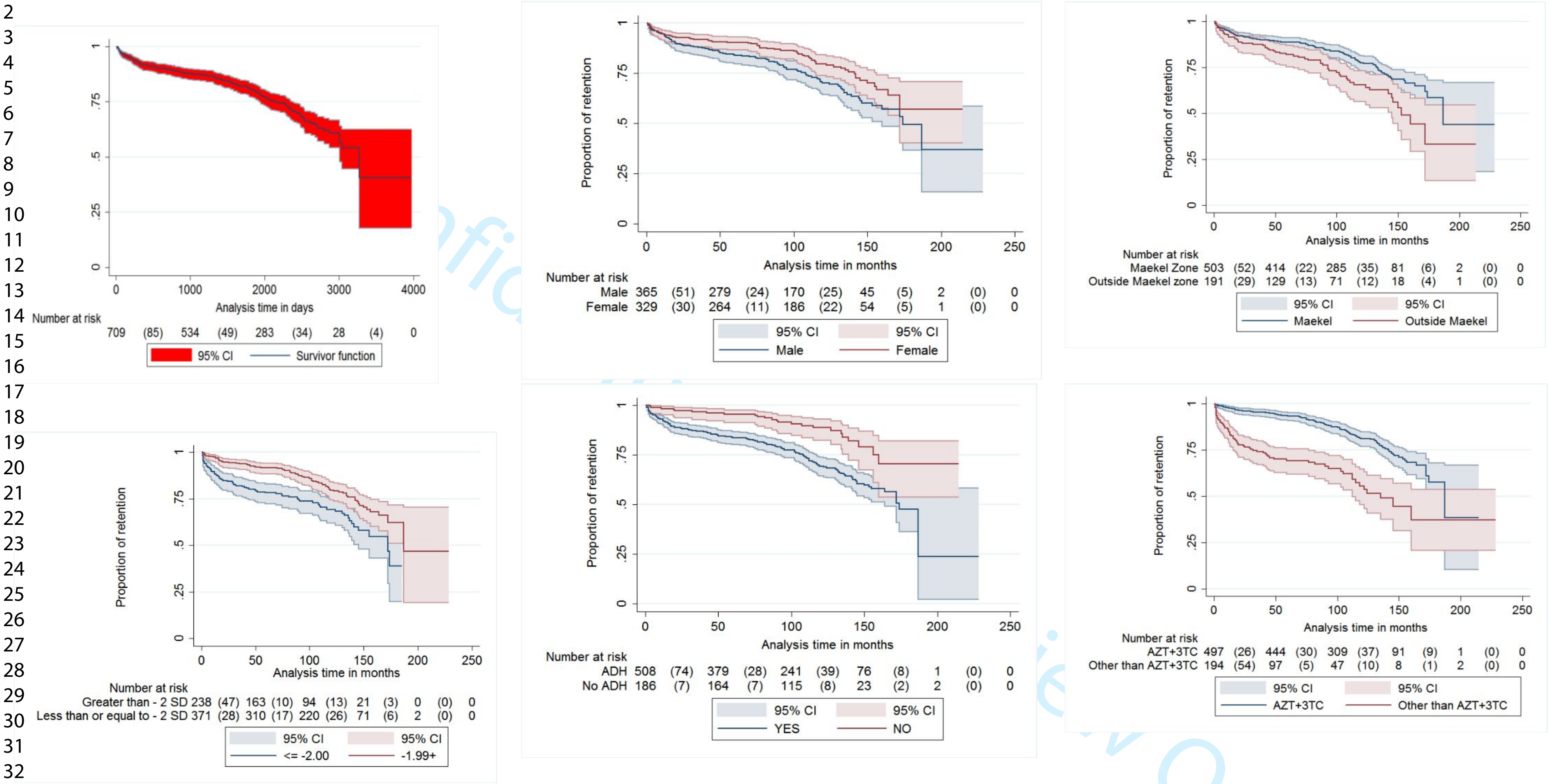

Figure 3. Kaplan-Meier cumulative incidence of attrition unadjusted curves for children followed in ONPRH from 2005- 2020

Table 3. Crude incidence, rate of attrition, and Kaplan-Meier survival estimates of children followed in the ONPRH cART treatment center, Asmara, Eritrea (2005–2020).

Cohort Characteristics	Incidence of attrition per 100 person years (95% CI)	Mean survival duration in years, 95% CI	P-value (log-rank)
**Total**	3.2 (2.7-3.7)	14.1 (13.4-14.8)	
**Gender**			
Male	3.8 (31.-4.6)	13.2 (2.1-14.3)	**0.008 (6.9)**
Female	2.5 (2-3.2)	13.8 (13.1-14.4)	
**Age in years at enrollment**			
<=5 years	2.5 (1.9-3.2)	15.1 (14.2-16)	
6-10 years	2.8 (2.2-3.6)	11.7 (11.1-12.2)	**<0.001 (36.8)**
>10 years	6.7 (5-8.9)	8.4 (7.43-9)	
**Address**			
Maekel	2.7 (2.3-3.3)	14.6 (13.8-15.4)	**0.002 (9.8)**
Outside Maekel	4.6 (3.5-5.9)	10.9 (10-11.7)	
**Disease Stage at presentation**			
Early	1.4 (0.9-2.1)	15 (14.2-15.7)	**<0.001 (22)**
Advanced	3.9 (3.3-4.6)	13.2 (12.3-14)	
**TB-Status**			
Symptomatic and under treatment	3.1 (2.6-3.7)	9.9 (6.9-12.8)	0.2 (1.5)
Not symptomatic	6.5 (2.7-15.8)	14 (13.3-14.8)	
**Hemoglobin**			
Anemia	2.2 (1.7-2.9)	11.5 (11-12)	**0.004 (8.3)**
Normal	3.7 (2.9-4.7)	12.8 (12-13)	
**WAZ**			
WAZ=>-2	2.1 (1.48-3.1)	13 (12.4-13.7)	**0.014 (6)**
WAZ <-2	3.5 (2.8-4.4)	12 (11.4-12.8)	
**HAZ**			
HAZ=>-2	2.3 (1.7-3.2)	14.9 (13.7-16)	**0.019 (5.4)**
HAZ <-2	3.5 (3-4.2)	12.7 (12-13.4)	
**BAZ**			
BAZ=>-2	2.4 (1.9-3)	15 (14-15.9)	**<0.001 (12.4)**
BAZ <-2	4.4 (3.5-5.5)	12 (11-13)	
**Initial cART backbone**			
AZT+3TC	2.3 (1.9-2.8)	13.9 (13.4-14.5)	**<0.001 (54.5)**
Other backbones	7.1 (5.6-9)	11.3 (9.8-12.8)	
**NNRTI**			
EFV	3.2 (2.6-3.9)	13 (12.3-13.7)	0.9 (0.015)
NVP	3 (2.5-3.9)	14.2 (13.3-15.2)	
**Current cART regimen**			
First line cART	3.34 (2.8-3.9)	12.9 (12.3-13.4)	0.15 (2)
Second line cART	2.2 (1.3-3.7)	15.7 (14.3-17)	
**Adherence**			
Suboptimal Adherence	2.9 (1.9-4.3)	12.6 (11.8-13.4)	0.68 (0.16)
No record	3.2 (2.7-3.7)	14.3 (13.6-15)	
**cART Change**			
cART change	1 (0.84-1.3)	13.6 (13-14)	**<0.001 (136)**
No cART change	2 (1.7-2.5)	7.5 (6.6-8.4)	

Abbreviations: cART; combined antiretroviral therapy, NNRTI; non-nucleoside reverse transcriptase inhibitor, WAZ; weight for age standard deviations, HAZ; height for age standard deviations, and BAZ; Body mass index for age standard deviations
Definitions: **Other back bones** refer to Tenofovir disoproxil fumarate (TDF) + Emtricitabine (FTC), Abacavir (ABC) + 3TC or Stavudine (D4T) + 3TC. **Anemia** was defined as hemoglobin (Hg) level is < 11 g/dl for children <5 years old, <11.5 g/dl for children 5–11.9 years old, and <12 g/dl for children >12 years old.
Comparisons of survival duration were performed by using the Log-rank test and its p-value.

Confidential: For Review Only

Independent predictors of attrition rate in children enrolled in ONPRH cART treatment and follow-up clinic

The hazard ratios (unadjusted and adjusted) for attrition in 701 patients and associated p-values for differences in attrition across specific cohort characteristics are shown in Table 4. In the univariate Cox proportional hazards model, increased risk of attrition was associated with the following: males (UHR=1.4, 95% CI: 1–2, p=0.01); residence outside Zoba Maekel (UHR=1.6, 95% CI: 1.2–2.2, p=0.002); WHO BAZ score <-2 (UHR=1.8, 95% CI: 1.3–2.5, p=0.001); WHO HAZ score (UHR=1.5, 95% CI: 1–2.1, p=0.02); presence of anaemia (UHR=1.7, 95% CI: 1.7 1.2–2.4, p=0.005); advanced disease (WHO III or IV) at presentation (UHR=2.9, 95% CI: 1.8–4.3, p<0.001); initiation of AZT+3TC or other cART backbones (UHR=3, 95% CI: 2.2–4.2, p<0.001). In contrast, reduced risk of attrition was associated with cART changes (UHR=0.16, 95% CI: 0.12–0.2, p<0.001).

In the adjusted Cox proportional hazards model, independent predictors of attrition were male sex (AHR=1.6, 95% CI: 1–2.4, p=0.01), residence outside Zoba Maekel (AHR=1.5, 95% CI: 1–2.3, p=0.02); later enrolment years (2010-2015: AHR=3.2, 95% CI: 1.9–5.3, p<0.001) (>2015: UHR=6.1, 95% CI: 3–12.2, p<0.001); BAZ < -2 (AHR=1.4, 95% CI: 0.9–2.1, p=0.05); advanced HIV disease (WHO III or IV) at enrolment (AHR=2.2, 95% CI: 1.2–3.9, p=0.004); initiation of AZT+3TC or other cART backbones (UHR=2, 95% CI: 1.2–3.2, p=0.005). In contrast, a reduced likelihood of attrition was observed in children with a record of cART changes (UHR=0.2, 95% CI: 0.15–0.4, p<0.001). Table 4.

https://mc.manuscriptcentral.com/bmjpo

Table 4. Cox proportional hazards of attrition among CLHIV and adolescents at ONPRH cART treatment center, Asmara, Eritrea (2005–2020)

Cohort Characteristics	Unadjusted HR (95%CI)	p-value	Adjusted HR (95%CI)	p-value
**Gender**				
Female	1 (*Ref.*)	**0.01**	1 (*Ref.*)	**0.01**
Male	1.4 (1-2)		1.6 (1-2.4)	
**Age at enrollment**				
<= 5	1 (*Ref.*)		1 (*Ref.*)	
6-10	1.3 (0.9-1.8)		1.1 (0.7-1.6)	
>10	3.3 (2.2-5)		1.5 (0.9-2.5)	
**Address**				
Maekel	1 (*Ref.*)	**0.002**	1 (*Ref.*)	**0.02**
Outside Maekel	1.6 (1.2-2.2)		1.5 (1-2.3)	
**Year of Enrollment**				
< 2010	1 (*Ref.*)		1 (*Ref.*)	
2010-2015	1.9 (1.3-2.7)		3.2 (1.9-5.3)	**<0.001**
>2015	4.2 (2.5-7.2)		6.1 (3-12.2)	**<0.001**
**BMI for age, z-score**				
**>= 2**	1 (*Ref.*)	**0.001**	1 (*Ref.*)	**0.03**
**< -2**	1.8 (1.3-2.5)		1.4 (0.9-2.1)	
**Height for age, z-score**				
**>= 2**	1 (*Ref.*)	**0.02**		
**< -2**	1.5 (1-2.1)			
**Hemoglobin**				
Normal	1 (*Ref.*)	**0.005**		
Anemia	1.7 (1.2-2.4)			
**Advanced disease at presentation**				
No	1 (*Ref.*)	**<0.001**	1 (*Ref.*)	**0.004**
Yes	2.9 (1.8-4.3)		2.2 (1.2-3.9)	
**TB status**				
Not symptomatic	1 (*Ref.*)	0.23		
Took Anti-TB	1.8 (0.8-5)			
**cART Backbone**				
AZT+3TC	1 (*Ref.*)	**<0.001**	1 (*Ref.*)	**0.005**
Other backbones	3 (2.2-4.2)		2 (1.2-3.2)	
**NNRTI**				
EFV	1 (*Ref.*)	0.9		
NVP	0.95 (0.7-1.2)			
**cART changes**				
No	1 (*Ref.*)	**<0.001**	1 (*Ref.*)	**<0.001**
Yes	0.16 (0.12-0.2)		0.2 (0.15-0.4)	
**Current cART regimen**				
**First line cART**	1 (*Ref.*)	0.16		
**Second line cART**	0.68 (0.4-1.1)			
**Suboptimal adherence**				
No	1 (*Ref.*)	0.2		
Yes	1.1 (0.7-1.7)			

Abbreviations: cART; combined antiretroviral therapy, NNRTI; non-nucleoside reverse transcriptase inhibitor, WAZ; weight for age standard deviations, HAZ; height for age standard deviations, and BAZ; Body mass index for age standard deviations

Definitions: **Other back bones** refer to Tenofovir disoproxil fumarate (TDF) + Emtricitabine (FTC), Abacavir (ABC) + 3TC or Stavudine (D4T) + 3TC. **Anemia** was defined as hemoglobin (Hg) level is < 11 g/dl for children <5 years old, <11.5 g/dl for children 5–11.9 years old, and <12 g/dl for children >12 years old.

Confidential: For Review Only

Confidential: For Review Only

DISCUSSION

Attrition rates among those receiving care were lower than those reported in similar studies in SSA.[6] However, our finding of high attrition and mortality rates in the first six months of treatment is consistent with multiple reports.[5 13 19] In the multicounty treated Asia Paediatric HIV Observational Database, mortality decreased from 10.2 (95% CI: 7.5–13.7) deaths/100 PY in the first 3 months to 4.2 (95% CI: 2.6–6.8) deaths/100 PY) in the fourth and sixth months.[18] The high mortality in the first six months of enrolment in care has largely been attributed to late presentation (WHO stage III or IV), chronic diarrhoea, and weight for height z-score <-2 (severe wasting). Observational studies suggest that the first six months after treatment initiation is the most vulnerable period for HIV-positive children in SSA.[5] Studies consistently cite the need for context-specific mitigation (short messaging service [SMS]-based messaging/reminder; improved linkage of child and maternal HIV/AIDS services; intensive case management),[5] directed at maximizing retention at this stage.

Furthermore, analysis of attrition incidence demonstrated a rate of 3.2 (95% CI: 2.7–3.7)/100 PY. This rate estimate is lower compared to studies conducted in Ethiopia (8.36[95% CI: 7.12–9.80]/100 PY),[14] Zimbabwe (11.8, [95% CI:11.0–12.7]/100 PY),[15] Nigeria (10.8/100 PY), [16] and Kenya (23.1/100 PY).[17] In contrast, the incidence of attrition in this study was higher compared to other studies.[18 19 20] Variations in incidence between countries in SSA is caused by differences in access to and quality of healthcare services, health-seeking behaviours between populations;[21] heterogeneity in methodology, and definition of outcomes. Finally, it is possible that the LTFUs registered in our study may be due to undocumented mortality instead of disengagement from care or undocumented transfers. Therefore, the mortality rate may have been underestimated.

In many respects, the predictors of attrition identified in this study are largely similar to other regional studies. For instance, evidence from multiple studies in the region indicate that WHO BAZ<-2 (undernutrition) and BMI for age z-score <-2 are baseline hazards for cART attrition.[8 10 15] Although the presence of anaemia at baseline was associated with an increased risk of attrition (1.7; 95% CI:1.2–2.4), the association was attenuated in the adjusted multivariable model. This suggests the presence of confounders. Nevertheless, the conjunction of these parameters should not be ignored. Several reports suggest that severe malnutrition (SAM) and HIV is associated with increased morbidity and mortality in affected infants.[22 23] Others have argued that HIV+SAM represents a distinct disease stratum/phenotype with a worse prognosis and impaired immune recovery [24]. Importantly, managing these patients is extremely challenging.[12] Highlighting this point, some investigators suggest that rapid initiation (within 1 week) of cART in children with SAM or WHO stage III or IV disease may be counterproductive, and that treatment in these patients should be delayed until the patient is stabilized.[23] Based on these considerations, the observed connection between advanced HIV disease (III or IV) at enrolment and attrition rate (aHR=2.2, 95% CI: 1.2–3.9) was predictable.[13] Reports from Zimbabwe,[15] Nigeria,[16] and Myanmar,[25] have demonstrated that late presentation remains a challenge in this region.

Additional factors unique to this setting that were associated with attrition included male sex, residence outside Asmara, enrolment in later years, and late presentation. In general, data on the relationship between sex and attrition are inconsistent, with most studies reporting a null relationship, while others skew toward either sex.[8 26 27] We believe that these inconsistencies and paradoxical results in cohorts across SSA mirror the disparate context-specific influences in the region. In contrast, the observed relationship between attrition and residence outside Asmara has greater practical implications. First, experts mention that HIV-related discrimination and internalized shame may constrain access to healthcare among adults living with HIV (ALWH).[28]

Confidential: For Review Only

For context, ridicule from friends/peers may force guardians/parents to enroll patients in distant facilities outside Zoba, Maekel. This practice can compromise linkage and retention in care by imposing unnecessary out-of-pocket costs (transport).[29-31] Fear of victimization can also prompt delays or failure by parents/guardians to disclose perinatally acquired HIV status,[32] - a phenomenon which can promote late presentation. In many ways, the highlighted connection between stigma, attrition, enrolment in later years, and late presentation is a common finding in attrition studies on ALWH.[13 19 22] We concede that HIV programs in Eritrea are ill-equipped to deal with the complex needs of ALWH. Multiple studies demonstrated that ALWH recruitment and retention in care is undermined by a lack of psychosocial support, lack of health personnel trained on ALWH –related issues, barriers to care uptake, and limited support during the transition to adult care.[33] Implementing comprehensive interventions targeting these barriers will require robust research.

Lastly, in the multivariable proportional hazard regression, the adjusted hazards of attrition were highest in children without cART changes or on other cART backbones (Tenofovir disoproxil fumarate (TDF) + Emtricitabine (FTC), Abacavir (ABC) + 3TC or Stavudine (D4T) + 3TC). A causal analysis of these associations revealed some subtle connections. Retention in care may correlate with a higher likelihood of cART changes/switching to other backgrounds. In contrast, late presentation is associated with a higher likelihood of mortality/attrition within the first six months of enrollment.[34] This can undercut the prospects of cART switches, particularly in settings with a limited range of cART options. If valid, then the latter explanation highlights a major systematic weakness in cART clinics across Eritrea. There is limited diversity of cART options for CLHIV and ALHIV.

https://mc.manuscriptcentral.com/bmjpo

Confidential: For Review Only

Strength and limitations of the study

A major strength of this study is the use of routinely collected information from the largest paediatric cART clinic in Eritrea. Therefore, the results can provide a useful gauge of real-world program successes and weaknesses. However, our study has limitations. Retrospective designs suffer from multiple constraints, including missing data. Missing or unstructured collection of information ($CD4^+$ cell counts or viral load data) and lack of data on the causes of death and outcomes in children characterized as LTFU are particular examples. In the absence of tracing information, mortality estimates in this cohort are unclear.

DECLARATIONS

Ethical approval and consent to participate

Ethical approval for this study was obtained from the Eritrean Ministry of Health (MOH) Research Ethics Committee. Information on maintaining data confidentiality and integrity was also provided. As the study is based on patients' clinical card records, consent for data access was waived by the ethical committee in place of the patients. This study conformed to the principles outlined in the Declaration of Helsinki.

Consent for publication

Consent for publication was waived by the ethical committee in place of patients, as the data were secondary.

Availability of data and materials

The datasets supporting the conclusions of this study are available from the corresponding author upon reasonable request.

Confidential: For Review Only

Conflict of Interest

The authors have no conflict of interest to declare in this study.

Financial support

The study hasn't received any funding.

Authors' contributions

Conceptualization: Samuel Tekle Mengistu

Data curation: Samuel Tekle Mengistu, and Ghirmay Ghebrekidan,

Formal analysis: Samuel Tekle Mengistu and Oliver Okoth Achila

Investigation: Samuel Tekle Mengistu, Mohammed Elfatih Hamida and Oliver Okoth Achila.

Methodology: Samuel Tekle Mengistu, Ghirmay Ghebrekidan, and Aron Rezene

Project administration: Samuel Tekle Mengistu, Mahmud Mohammed Idris, Tsegereda Gebrehiwot Tikue, Mohammed Elfatih Hamida, and Oliver Okoth Achila

Writing – original draft: Samuel Tekle Mengistu, and Oliver Achila.

Writing – review& editing: Samuel Tekle Mengistu, Ghirmay Ghebrekidan Ghebremeskel, Oliver Achila, Mahmud Mohammed Idris, Tsegereda Gebrehiwot Tikue, Mohammed Elfatih Hamida, and Oliver Okoth Achila.

Acknowledgment

We are grateful to the National Communicable Disease Control Division, Eritrean Ministry of Health, and ART Health Management Information System (HMIS) developers and technicians. Our sincere appreciation goes to Dr. Araia Berhane, Dr. Fanus Yemane, Dr. Simon Tesfay, Dr. Yonathan Tesfalidet and Mr. Thomas Asfaha.

Confidential: For Review Only

Reference

1. Levi J, Raymond A, Pozniak A, et al. Can the UNAIDS 90-90-90 target be achieved? A systematic analysis of national HIV treatment cascades. *BMJ global health* 2016;1(2):e000010.

2. Bain LE, Nkoke C, Noubiap JJN. UNAIDS 90–90–90 targets to end the AIDS epidemic by 2020 are not realistic: comment on "Can the UNAIDS 90–90–90 target be achieved? A systematic analysis of national HIV treatment cascades". *BMJ global health* 2017;2(2):e000227.

3. Ng'eno B, Mwangi A. Burden of HIV infection among children aged 18 months to 14 years in Kenya: results from a nationally representative population-based cross-sectional survey. *Journal of acquired immune deficiency syndromes (1999)* 2014;66(Suppl 1):S82.

4. Desmonde S, Tanser F, Vreeman R, et al. Access to antiretroviral therapy in HIV-infected children aged 0–19 years in the International Epidemiology Databases to Evaluate AIDS (IeDEA) Global Cohort Consortium, 2004–2015: a prospective cohort study. *PLoS medicine* 2018;15(5):e1002565.

5. Carlucci JG, Liu Y, Clouse K, et al. Attrition of HIV-positive children from HIV services in low-and middle-income countries: a systematic review and meta-analysis. *AIDS (London, England)* 2019;33(15):2375.

6. Phelps BR, Ahmed S, Amzel A, et al. Linkage, initiation and retention of children in the antiretroviral therapy cascade: an overview. *AIDS (London, England)* 2013;27(0 2):S207.

7. Division CDC. ERITREAN ANTIRETROVIRALTHERAPY GUIDELINES. Asmara, Eritrea: Ministry of Health, 2017.

Confidential: For Review Only

8. Zanoni BC, Phungula T, Zanoni HM, et al. Risk factors associated with increased mortality among HIV infected children initiating antiretroviral therapy (ART) in South Africa. *PloS one* 2011;6(7):e22706.

9. Fenner L, Brinkhof MW, Keiser O, et al. Early mortality and loss to follow-up in HIV-infected children starting antiretroviral therapy in Southern Africa. *Journal of acquired immune deficiency syndromes (1999)* 2010;54(5):524.

10. Brophy JC, Hawkes MT, Mwinjiwa E, et al. Survival Outcomes in a Pediatric Antiretroviral Treatment Cohort in Southern Malawi. *PLoS One* 2016;11(11):e0165772. doi: 10.1371/journal.pone.0165772 [published Online First: 2016/11/05]

11. Htun T, Kyaw K, Aung T, et al. Attrition during pre-ART and ART time periods among adolescents enrolled in Integrated HIV Care Programme in Myanmar, 2005–2017. *Epidemiology & Infection* 2019;147

12. Organization WH. Guidelines for managing advanced HIV disease and rapid initiation of antiretroviral therapy, July 2017. 2017

13. Mutanga JN, Mutembo S, Ezeamama AE, et al. Long-term survival outcomes of HIV infected children receiving antiretroviral therapy: an observational study from Zambia (2003–2015). *BMC public health* 2019;19(1):1-12.

14. Demeke Dejen DJ, Yeshanew F, Fentaw Z, et al. Attrition and Its Predictors Among Adults Receiving First-Line Antiretroviral Therapy in Woldia Town Public Health Facilities, Northeast Ethiopia: A Retrospective Cohort Study. *HIV/AIDS (Auckland, NZ)* 2021;13:445.

15. Makurumidze R, Mutasa-Apollo T, Decroo T, et al. Retention and predictors of attrition among patients who started antiretroviral therapy in Zimbabwe's national antiretroviral therapy programme between 2012 and 2015. *PloS one* 2020;15(1):e0222309.

Confidential: For Review Only

16. Onubogu CU, Ugochukwu EF. A 17 year experience of attrition from care among HIV infected children in Nnewi South-East Nigeria. *BMC Infectious Diseases* 2021;21(1):1-11.

17. Hassan AS, Mwaringa SM, Ndirangu KK, et al. Incidence and predictors of attrition from antiretroviral care among adults in a rural HIV clinic in Coastal Kenya: a retrospective cohort study. *BMC Public Health* 2015;15(1):1-9.

18. Decroo T, Koole O, Remartinez D, et al. Four-year retention and risk factors for attrition among members of community ART groups in T ete, M ozambique. *Tropical Medicine & International Health* 2014;19(5):514-21.

19. Teeraananchai S, Kerr SJ, Puthanakit T, et al. Attrition and mortality of children receiving antiretroviral treatment through the universal coverage health program in Thailand. *The Journal of pediatrics* 2017;188:210-16. e1.

20. Cohen S, Smit C, van Rossum AM, et al. Long-term response to combination antiretroviral therapy in HIV-infected children in the Netherlands registered from 1996 to 2012. *Aids* 2013;27(16):2567-75.

21. Bimer KB, Sebsibe GT, Desta KW, et al. Incidence and predictors of attrition among children attending antiretroviral follow-up in public hospitals, Southern Ethiopia, 2020: a retrospective study. *BMJ paediatrics open* 2021;5(1)

22. Desai SN, Landay AL. HIV and aging: role of the microbiome. *Current Opinion in HIV and AIDS* 2018;13(1):22-27.

23. Frigati L, Archary M, Rabie H, et al. Priorities for decreasing morbidity and mortality in children with advanced HIV disease. *Clinical Infectious Diseases* 2018;66(suppl_2):S147-S51.

24. Fouda GG, De Paris K, Levy O, et al. Immunological mechanisms of inducing HIV immunity in infants. *Vaccine* 2020;38(3):411-15.

Confidential: For Review Only

25. Minn AC, Kyaw NTT, Aung TK, et al. Attrition among HIV positive children enrolled under integrated HIV care programme in Myanmar: 12 years cohort analysis. *Global health action* 2018;11(1):1510593.

26. Mugglin C, Wandeler G, Estill J, et al. Retention in care of HIV-infected children from HIV test to start of antiretroviral therapy: systematic review. *PLoS One* 2013;8(2):e56446. doi: 10.1371/journal.pone.0056446 [published Online First: 2013/02/26]

27. Apondi E, Humphrey JM, Sang E, et al. Trends Over Time for Adolescents Enrolling in HIV Care in Kenya, Tanzania, and Uganda From 2001-2014. *J Acquir Immune Defic Syndr* 2018;79(2):164-72. doi: 10.1097/qai.0000000000001796 [published Online First: 2018/07/10]

28. Pantelic M, Casale M, Cluver L, et al. Multiple forms of discrimination and internalized stigma compromise retention in HIV care among adolescents: findings from a South African cohort. *Journal of the International AIDS Society* 2020;23(5):e25488.

29. Lankowski AJ, Siedner MJ, Bangsberg DR, et al. Impact of geographic and transportation-related barriers on HIV outcomes in sub-Saharan Africa: a systematic review. *AIDS and Behavior* 2014;18(7):1199-223.

30. Carlos S, Burgueño E, Ndarabu A, et al. Predictors of retention in the prospective HIV prevention OKAPI cohort in Kinshasa. *Scientific Reports* 2021;11(1):1-10.

31. Geng EH, Bangsberg DR, Musinguzi N, et al. Understanding reasons for and outcomes of patients lost to follow-up in antiretroviral therapy programs in Africa through a sampling-based approach. *Journal of acquired immune deficiency syndromes (1999)* 2010;53(3):405.

32. Krauss BJ, Letteney S, Okoro CN. Why tell children: A synthesis of the global literature on reasons for disclosing or not disclosing an HIV diagnosis to children 12 and under. *Frontiers in Public Health* 2016;4:181.

Confidential: For Review Only

33. Dahourou DL, Gautier-Lafaye C, Teasdale CA, et al. Transition from paediatric to adult care of adolescents living with HIV in sub-Saharan Africa: challenges, youth-friendly models, and outcomes. *Journal of the International AIDS Society* 2017;20:21528.

34. Bigna JJ, Plottel CS, Koulla-Shiro S. Challenges in initiating antiretroviral therapy for all HIV-infected people regardless of CD4 cell count. *Infect Dis Poverty* 2016;5(1):85. doi: 10.1186/s40249-016-0179-9 [published Online First: 2016/09/07]

Confidential: For Review Only

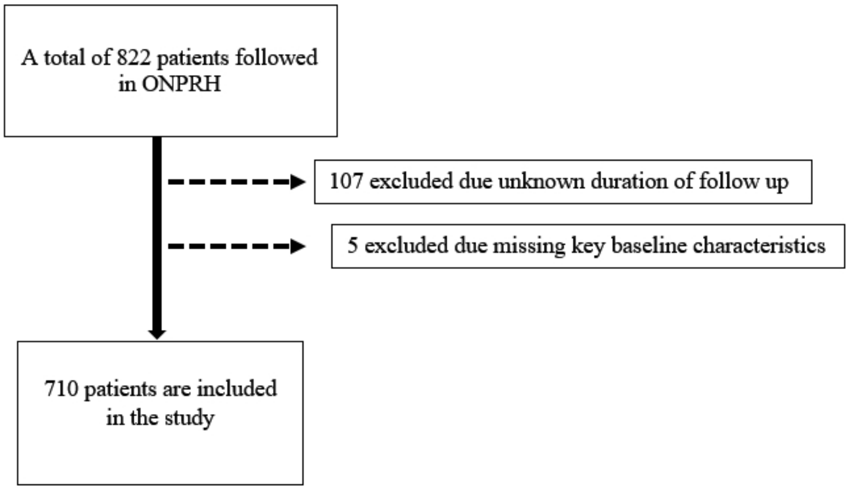

508x258mm (38 x 38 DPI)

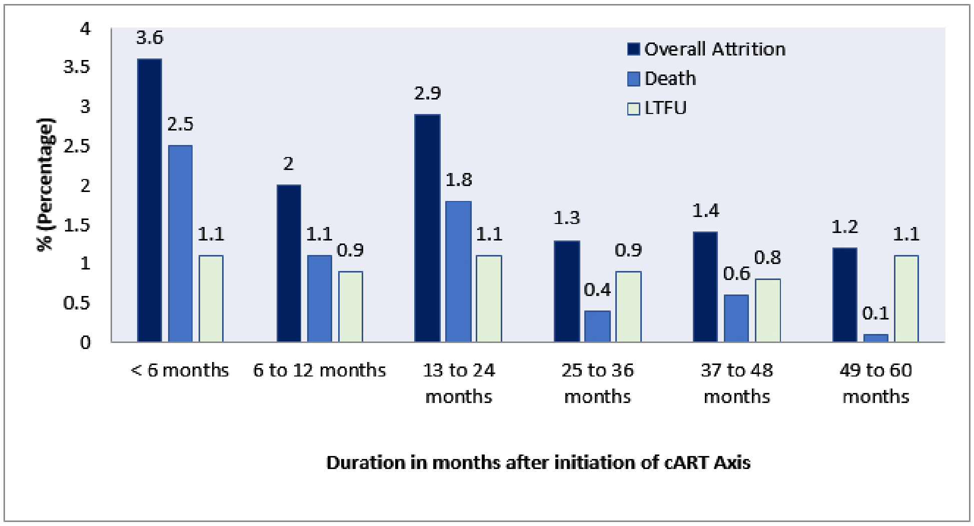

455x249mm (38 x 38 DPI)

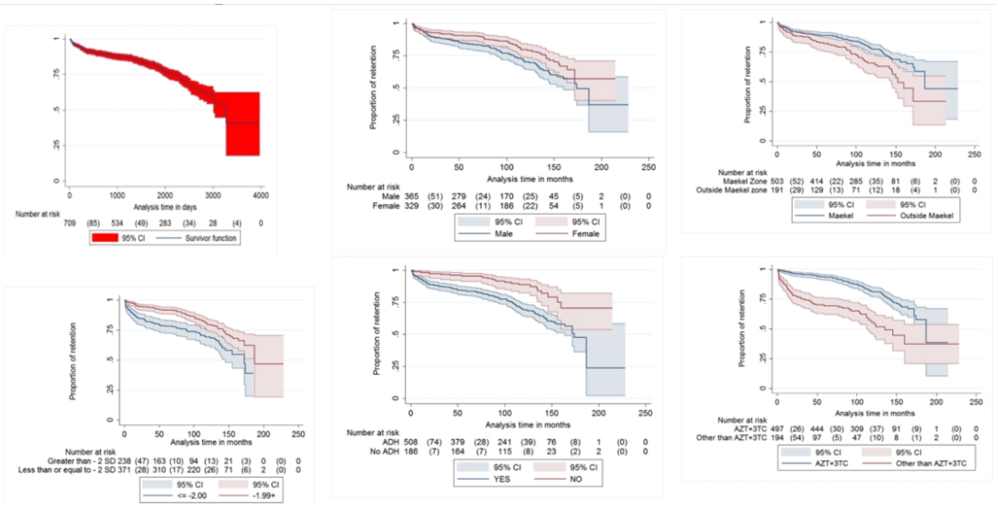

784x394mm (38 x 38 DPI)
